# Calix[6]arenes with halogen bond donor groups as selective and efficient anion transporters†

**DOI:** 10.1039/d2cc00847e

**Published:** 2022-05-06

**Authors:** Anurag Singh, Aaron Torres-Huerta, Tom Vanderlinden, Nathan Renier, Luis Martínez-Crespo, Nikolay Tumanov, Johan Wouters, Kristin Bartik, Ivan Jabin, Hennie Valkenier

**Affiliations:** Université libre de Bruxelles (ULB), Ecole polytechnique de Bruxelles, Engineering Molecular NanoSystems Avenue Franklin Roosevelt 50 1050 Brussels Belgium hennie.valkenier@ulb.be; Université libre de Bruxelles (ULB), Faculty of science, Laboratoire de Chimie Organique Avenue Franklin Roosevelt 50 1050 Brussels Belgium; Namur Institute of Structured Matter and Namur Research Institute for Life Sciences, Department of Chemistry, University of Namur 61 rue de Bruxelles B-5000 Namur Belgium

## Abstract

Here we present the anion binding and anion transport properties of a series of calix[6]arenes decorated on their small rim with either halogen bond or hydrogen bond donating groups. We show that the halogen bond donating iodotriazole groups enable highly selective transport of chloride and nitrate anions, without transport of protons or hydroxide, at rates similar to those observed with thiourea or squaramide groups.

## Abstract 2

A calix[6]arene with three preorganised halogen bond donating groups gives >100-fold selectivity for Cl^−^ uniport over HCl symport, in contrast to analogous compounds with strong hydrogen bond donating groups.

## Graphical abstract

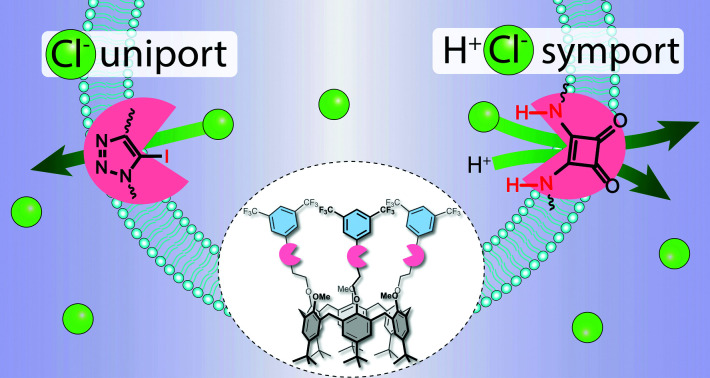

Synthetic ion transporters have potential for the treatment of channelopathies, such as cystic fibrosis, linked to deficient ion transport.^1,2^ Most anion transporters described in the literature rely on hydrogen bonding (HB) between relatively acidic hydrogen atoms and the anion.^3^ However, deprotonation of these acidic HB donors can lead to a net transport of protons along with the anions.^4,5^ Proton transport is linked to an increasing risk of toxicity,^6^ which is interesting in the context of anti-cancer activity, but undesirable for treatment of channelopathies. Selectivity for (i) anion transport without dissipation of pH gradients and (ii) certain anions over others represent major challenges in anion transport.^7^

A strategy to achieve selective transport of anions is the use of less acidic H-bond donors,^4,8,9^ or interactions based on Sigma holes.^10^ A Sigma hole is an electron deficient region on a bound halogen, chalcogen, pnictogen, or tetrel atom, which can interact with electron rich entities, such as anions.^11–13^ This interaction is referred to as halogen bond (XB) if it involves a halogen atom, which are typically less toxic than the other atoms which can bear a Sigma hole. The use of XBs in transport has been pioneered by Matile and co-workers.^14^ Various XB-based transporters have been studied^15–18^ and a 5-fold selectivity for transport of chloride over HCl transport by an XB donor was recently reported by Langton, Beer, and co-workers.^19^ However, published studies compare transporters with XB donors to those with an H atom in the place of the halogen atom, rather than comparing XB donors to powerful HB donating motifs, such as (thio)ureas and squaramides.^20–22^

Here we present a comparison between XB and HB donating compounds in terms of anion binding, transport activity, and selectivity, using receptors with (Fig. 1): iodotriazoles as XB donors (1a), regular triazoles as control (2), and squaramides or (thio)ureas as strong HB donors (3–5). Three of these groups were attached to the small rim of a calix[6]arene to effectively bind the anion and shield its charge from the apolar interior of the membrane. The value of this calix[6]arene-based design was demonstrated previously by the activity of tris-urea 4 and tris-thiourea 5 as anion receptors^23^ and carriers.^24^ The larger iodotriazole binding groups are also readily accommodated by the calix[6]arene platform.^25^ Electron-poor bis(CF_3_)phenyl rings were used to increase the binding strength of the various motifs.^20–24^ These groups and the calix[6]arene skeleton also ensure that the overall lipophilicity and lipophilic balance^26^ of the studied receptors is similar (see Table 1 for c log *P* values). Two additional calix[6]arenes bearing iodotriazole units with *p*NO_2_phenyl (1b) and phenyl groups (1c) were prepared to evaluate the effect of the electron withdrawing substituents on binding and transport by XB donors.

**Fig. 1:**
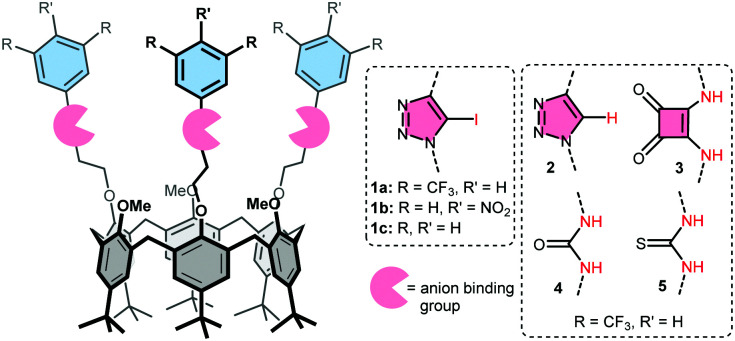
Structure of calix[6]arene-based transporters 1a–c, 2–5.

**Table 1:** Calculated log *P* values, affinity constants for Cl^−^, and anion transport results for compounds 1–5

Receptor	c log *P*^a^	*K*_a_ in acetone (M^−1^)	*K*_a_ in CHCl_3_ (M^−1^)	Transport rate [*I*] (s^−1^) c	Transport selectivity^d^
1a	34	>10^5^	7.7·10^2^	34	>100
1b	29	n.d.^b^	1.3·10^2^	< 1	—
1c	29	8.9·10^3^	<10	< 1	—
2	32	58	<10	< 1	—
3	29	8.7·10^2^	n.d.^b^	45	1
4	29	1.4·10^3^	1.8·10^4^	12	1
5	32	1.4·10^3^	2.9·10^4^	25	1

a Calculated using MarvinSketch 19.25.

b Not determined due the low solubility.

c Initial rate of Cl^−^/NO_3_^−^ antiport in the lucigenin assay divided by the transporter to lipid ratio.

d Selectivity of Cl^−^ uniport/HCl symport, based on results from the HPTS assay in NMDGHCl.

Iodotriazole-based receptors 1a-c were prepared in 70–80% isolated yield from the reported calix[6]arene tris-azide 6,^27^*via* a cycloaddition reaction with the corresponding iodoalkynes 8a-c, in the presence of copper(i) and tris(benzyltriazolyl-methyl)amine (TBTA) as catalyst in THF^18^ (Scheme 1, see ESI† for details^28^). Triazole-based receptor 2 was prepared from calix[6]arene tris-azide 6 in 97% yield *via* a cycloaddition with alkyne 9 in the presence of 2,6-lutidine and a catalytic amount of copper(i) in DCM.^27^ The squaramide containing receptor 3 was isolated in 45% yield from the reaction of calix[6]arene tris-amine 7^27^ and compound 10 in methanol : DCM (9 : 1), using DIPEA as base.^29^ Receptors 4 and 5 were prepared following previously reported procedures.^23,24^

**Scheme 1:**
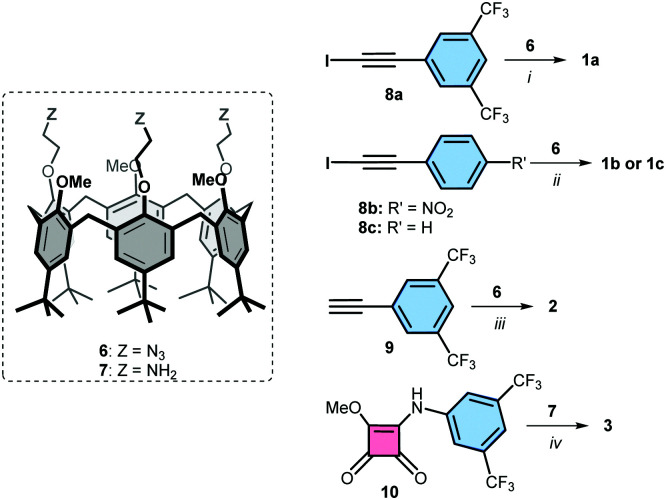
Synthesis of receptors 1-3 from precursor calix[6]arenes 6 and 7. Conditions: (i) CuI, TBTA, THF; (ii) [Cu(CH_3_CN)_6_]PF_6_, TBTA, THF; (iii) [Cu(CH_3_CN)_6_]PF_6_, 2,6-lutidine, DCM; (iv) DIPEA, MeOH, DCM.

The anion binding properties of receptors 1–5 were studied *via*^1^H-NMR titrations with tetrabutylammonium chloride (TBACl) in acetone-d_6_ or CDCl_3_. Binding constants were determined by fitting the shift of the signals of the protons closest to the binding site to a 1 : 1 model^30^ (see Table 1 and ESI†).^28^ For receptor 1a in acetone, the signals show a linear shift during the addition of a first eq. of TBACl, while no further changes are observed when adding up to 5 eq. TBACl, supporting a 1 : 1 binding mode in solution and indicating that the affinity is too high to be quantified. A *K*_a_ of 770 M^−1^ was found for 1a in chloroform and lower affinities were found for receptors 1b and 1c, which have fewer or no electron withdrawing groups on the aryl rings. Compound 2, which only has triazole groups, showed the weakest binding of Cl^−^. When comparing XB-based receptor 1a to HB-based receptors 3–5, which have the same aryl groups, we observe that, in acetone, 1a has an affinity which is at least two orders of magnitude higher than those of 3–5 (Table 1).^31^ This trend is inverted in CDCl_3_, in line with previous observations that XB donors show lower anion affinities in chloroform than in acetone.^32^ Titrations of 1a with various other anions revealed the following trend in affinities: Cl^−^ > Br^−^ ≫ H_2_PO_4_^−^ > NO_3_^−^, AcO^−^ (Table S1, ESI†), indicating selectivity for halides over oxoanions.

The molecular structure of receptor 1a determined by single-crystal X-ray diffraction (SCXRD, Fig. 2) shows that the methoxy groups of the calix[6]arene are oriented into the cavity and the arms oriented outward. The three XB donor iodotriazole groups coordinate one molecule of water by O⋯I interactions with a distance of 3.179(2) Å. The coordination sphere of the water molecule is completed by a second molecule of 1a to afford an octahedral geometry around the oxygen atom (Fig. 2c and Fig. S63, ESI†). This structural analysis shows that the three XB donor groups of 1a can indeed cooperate to act as an efficient receptor towards electronegative atoms, in agreement with the high affinity observed for chloride.

**Fig. 2:**
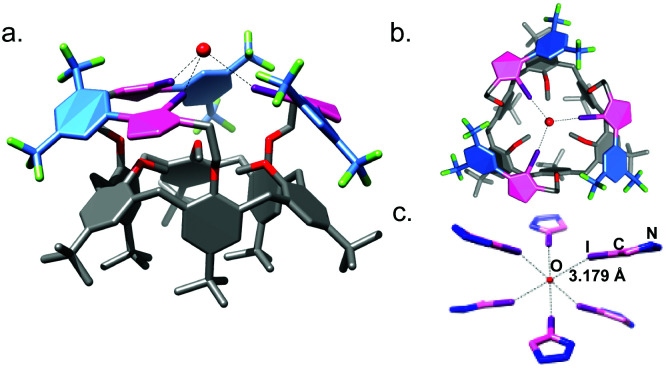
Molecular crystal structure of compound 1a, (a) side view and (b) top view. (c) O⋯I interactions between iodotriazole groups and the oxygen atom of a molecule of water. The iodotriazole groups are coloured in pink and bis(CF_3_)phenyl rings in blue and the hydrogen atoms and minor parts of the disorder are omitted for the sake of clarity.

Receptors 1–5 were studied as transmembrane transporters for Cl^−^ and NO_3_^−^ using the lucigenin assay.^20^ They were preincorporated in the lipid bilayer of large unilamellar vesicles (LUVs) that contained the chloride sensitive dye lucigenin in a NaNO_3_ solution. A NaCl pulse was added to create a chloride concentration gradient and the quenching of the fluorescence of lucigenin was monitored to assess Cl^−^ influx. The transport of Cl^−^ into the vesicles is compensated by the transport of NO_3_^−^ out of the vesicles, ensuring the charge balance (Cl^−^/NO_3_^−^ antiport). XB-based receptor 1a shows clear transport activity at concentrations down to 1 transporter per 25 000 lipid molecules (0.004 mol%, 16 nM), 1b showed poor activity (too low to quantify), and 1c and 2 were inactive at 1 : 1000 transporter to lipid ratio (Fig. 3a). The decrease in activity with a decreasing number of electron withdrawing substituents on the aryl group and the absence of transport by 2 confirm the role of the Sigma hole in anion transport.

**Fig. 3:**
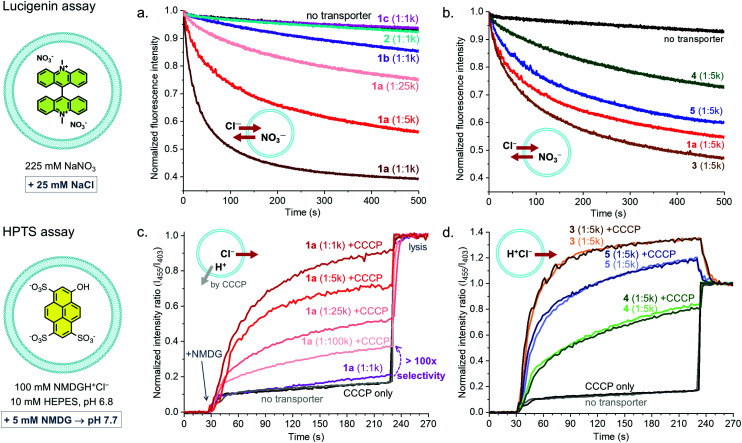
Anion transport studies by receptors 1-5 (preincorporated at the indicated transporter : lipid ratio) in LUVs of POPC/cholesterol (7 : 3 ratio). (a and b) Dissipation of a chloride gradient as monitored by the quenching of encapsulated lucigenin. (c and d) Dissipation of a pH gradient as monitored by changes in the fluorescence of HPTS; the protonophore CCCP (1 : 1000) was added prior to the start of the indicated experiments and the LUVs were lysed at 230 s.

When comparing XB-based receptor 1a to HB-based receptors 3–5, all with bisCF_3_phenyl groups, the highest transport rates were observed with tris-squaramide 3, followed by 1a and previously reported tris-thiourea 5, and the lowest rate was given by tris-urea 4 (Fig. 3b and Table 1). This demonstrates that receptors with XB donor groups can achieve similar transport activities as related receptors with powerful HB donor groups.

Having established the good activity of 1a, the transport selectivity of this compound in comparison to transporters 3-5 was studied. For this, the pH sensitive dye 8-hydroxypyrene-1,3,6-trisulfonic acid trisodium (HPTS) was encapsulated and a buffer solution with *N*-methyl-d-glucosamine hydrochloride (NMDGH^+^Cl^−^) and HEPES at pH 6.8 was used inside and outside the vesicles. Transport was initiated by addition of a base pulse (NMDG) giving an exterior pH of 7.7. In this assay, the dissipation of the pH gradient (by transport of H^+^ or OH^−^) can only be balanced by the transport of Cl^−^, as NMDGH^+^ is a membrane impermeable cation.^4^

Receptors 3–5 gave efficient transport, while no significant activity was observed with 1a even at a transporter:lipid ratio of 1 : 1000 (Fig. 3c and d). This shows that 1a is incapable of dissipating a pH gradient *via* either H^+^Cl^−^ symport or OH^−^/Cl^−^ antiport. Transport of HCl by 3–5 follows the same order as for Cl^−^/NO_3_^−^ antiport, with tris-squaramide 3 giving the highest rates, followed by tris-thiourea 5 and tris-urea 4. The experiments were also run in the presence of the H^+^ transporter carbonyl cyanide 3-chlorophenylhydrazone (CCCP). No impact was observed on the rates of transport by 3–5, indicating that transport of H^+^ is not rate limiting for these transporters with acidic H-bond donors. In contrast, the addition of CCCP clearly switched on the transport of 1a, even when present at concentrations as low as 1 : 100 000. This can be attributed to Cl^−^ uniport by 1a coupled to H^+^ transport by CCCP and shows that 1a has >100-fold selectivity for electrogenic Cl^−^ transport over H^+^Cl^−^ symport or OH^−^/Cl^−^ antiport. As 1a has no acidic protons, net transport of HCl by a mechanism involving deprotonation of a binding group upon anion release^4^ is indeed impossible. Furthermore, 1a has ∼20-fold higher selectivity compared to the recently reported transporter with two XB donor groups,^19^ which is likely to originate from the efficient encapsulation of Cl^−^ anions by the three XB donor groups on the calix[6]arene. These experiments demonstrate that transporter 1a has an unprecedented selectivity in combination with a good activity (EC_50_ of ∼0.007 mol%).

Similar experiments were performed, replacing Cl^−^ by NO_3_^−^ (NMDGH^+^NO_3_^−^ buffer), and selectivity of 1a for electrogenic NO_3_^−^ transport over H^+^NO_3_^−^ symport (or OH^−^/NO_3_^−^ antiport) was observed (Fig. S60, ESI†). The rate of NO_3_^−^ uniport by 1a was lower than that of Cl^−^ uniport. HB-based transporters 3–5 showed again hardly any selectivity. In contrast to 3–5, 1a was also found incapable of transporting OH^−^ or H^+^ in absence of transportable anions such as Cl^−^ and NO_3_^−^ (Fig. S61, ESI†). This remarkable selectivity of 1a was further highlighted using the lucigenin assay in NaHCO_3_ or NaOAc solutions to test for Cl^−^/HCO_3_^−^ and Cl^−^/AcO^−^ antiport (Fig. S57 and S58, ESI†). In both experiments clear activity was observed for transporter 3, but not for 1a. This shows that 1a is not only highly selective for Cl^−^ compared to HCl transport, but also for Cl^−^ compared to other anions. The anion transport selectivity of 1a follows the order: Cl^−^ > NO_3_^−^ ⋙ HCO_3_^−^, AcO^−^, OH^−^ and is correlated to its selectivity in anion binding.

In contrast, HB donors commonly show anion binding selectivities that correlate with the hydration energy or hydrogen bond accepting ability of the anions.^33,34^ As a result, HB-based receptors are very general transporters and only a slight selectivity of 5 for transport of NO_3_^−^ > Cl^−^ was observed.^35^ We note that, despite the poor selectivity, tris-squaramide 3 showed the highest transport activity of the series of compounds tested in all the transport assays used. This is in agreement with previous reports on squaramides with a single binding unit which outperformed ureas and thioureas,^21^ while attempts to achieve similar results in structures with two or three binding units often failed due to too strong binding or poor solubility of the squaramides.^29,36^

In conclusion, this study of transport activity and selectivity of lipophilic calix[6]arenes with different anion binding groups has revealed the remarkable selectivity of halogen bond donor 1a to transport Cl^−^ and NO_3_^−^, compared to the transport of other anions and to the dissipation of pH gradients. This high selectivity is accompanied by a transport activity on par with the analogous tris-thiourea 5 and close to tris-squaramide 3. This demonstrates that XB donor groups connected to a platform can achieve transport rates similar to those of powerful HB donors, while providing a superior selectivity for Cl^−^, which opens the way to therapeutic applications of XB donors for treatment of channelopathies such as cystic fibrosis, where Cl^−^ transport is required but dissipation of pH gradients could lead to undesired side effects.

We thank Koen Robeyns for his help with crystal structure refinement and Frank Meyer for discussions. The results reported here are part of a project that has received funding from the European Research Council (ERC) under the European Union's Horizon 2020 research and innovation programme (Grant agreement No. 802727). HV is a research associate of the Fonds de la Recherche Scientifique – FNRS. We thank the PC^2^ technological platform at the University of Namur for access to the single-crystal X-ray diffractometer.

## Conflicts of interest

There are no conflicts to declare.

## Supplementary Material

CC-058-D2CC00847E-s001

CC-058-D2CC00847E-s002
