# Supplementary material for: Calix[6]arenes with halogen bond donor groups as selective and efficient anion transporters
Source: Chem Commun (Camb). 2022 May 6;58(42):6255–8. doi: 10.1039/d2cc00847e (PMC9128489; doi:10.1039/d2cc00847e)
Supplement: CC-058-D2CC00847E-s001 [file CC-058-D2CC00847E-s001.pdf]

Electronic Supplementary Material (ESI) for ChemComm.
This journal is © The Royal Society of Chemistry 2022

Calix[6]arenes with halogen bond donor groups as selective and efficient anion transporters

Anurag Singh,[a] Aaron Torres Huerta,[a] Tom Vanderlinden,[a,b] Nathan Renier,[a] Luis Martínez-Crespo,[a] Nikolay Tumanov,[c] Johan Wouters,[c] Kristin Bartik,[a] Ivan Jabin,[b] Hennie Valkenier*[a]

a. *Université libre de Bruxelles (ULB), Engineering of Molecular NanoSystems, Ecole polytechnique de Bruxelles, Avenue F.D. Roosevelt 50, CP165/64, B-1050 Brussels, Belgium.*
b. *Université libre de Bruxelles (ULB), Laboratoire de Chimie Organique, Avenue F.D. Roosevelt 50, CP165/64, B-1050 Brussels, Belgium.*
c. *Namur Institute of Structured Matter and Namur Research Institute for Life Sciences, Department of Chemistry, University of Namur, 61 rue de Bruxelles, B-5000 Namur, Belgium.*

Table of contents

1. General experimental information 2
2. Synthesis 2
   Calix[6]arene **1a**: 3
   Calix[6]arene **1b**: 6
   Calix[6]arene **1c**: 9
   Calix[6]arene **2**: 12
   Calix[6]arene **3**: 15
3. Anion binding studies 18
   3.1 Binding studies in acetone 19
   3.2 Binding studies in chloroform 25
   3.3 Binding studies in DMSO 35
4. Transport studies in vesicles 36
   4.1 Lucigenin assay 36
   4.2 HPTS assay with a NMDG base pulse 38
   4.3 HPTS assay with a TBAOH base pulse 40
5. Single-crystal X-ray diffraction analysis 41

A dataset for this publication is available at Zenodo (https://doi.org/10.5281/zenodo.6010342) and contains:

- NMR spectra for the characterisation of compounds **1a**, **1b**, **1c**, **2**, and **3** (Mestrenova files)
- NMR spectra for the titration experiments with compounds **1-5** in different solvents (Mestrenova files)
- Concentrations of host and guests in the various titration experiments (Excel file)
- Transport data in the lucigenin assay (Excel file)
- Transport data in the HPTS assay (Excel file)

1. General experimental information

All reagents and solvents were obtained from Sigma Aldrich, Fluorochem, Alfa Aesar and VWR, and were used without further purification unless otherwise stated. 1-palmitoyl-2-oleoyl-*sn*-glycero-3-phosphocholine (POPC) and cholesterol were purchased from Sigma Aldrich and Acros, respectively. Lipid solutions of POPC and cholesterol were prepared using chloroform that had been deacidified by passage through a column containing basic alumina. POPC solutions were stored at -20 °C and cholesterol solutions were freshly prepared. All aqueous solutions were prepared using deionised water that had been passed through a Millipore filtration system. All liposomes were used within 4h after their preparation.

All NMR spectra for the characterization of compounds (^{1}H, ^{13}C, COSY, HSQC, and HMBC) were recorded on a Jeol JNM-ECZ400R/S3 spectrometer equipped with a 5 mm NM Royal probe at 298 K. ^{1}H NMR spectra for titrations were recorded on a Varian VNMRS 400 (9.4 T) spectrometer equipped with an OneNMR probe at 298 K. Solvent signals were used as reference for the chemical shifts. Chemical shifts are expressed in ppm and the coupling constants (J) are expressed in Hertz (Hz). The ^{1}H residual signals of the solvent were used as reference ($CDCl_3$ 7.26 ppm, DMSO-d_6 2.50 ppm, and acetone-d_6 2.05), as well as the ^{13}C signals of the solvent ($CDCl_3$ 77.16 ppm, DMSO-d_6 39.52 ppm, and acetone-d_6 206.26).

High-resolution mass spectra were measured on an Agilent QTOF 6520 by electron spray ionisation.

Fluorescence measurements were carried out on a FluoroMax-4 (Horiba) spectrofluorometer equipped with a water-thermostatted cell holder with stirring and an injection port.

2. Synthesis

Anhydrous THF was distilled over sodium and benzophenone. All the reactions were carried out under inert atmosphere with argon unless stated otherwise. For monitoring the progress of the reaction, thin layer chromatography was performed on silica gel 60 F_{254} coated aluminum sheets. The compounds were revealed using a UV light source of 254 nm. Whenever required, the purification was performed using column chromatography on silica gel (VWR chemicals, particle size 35-70 μm).

We obtained the compounds **6** and **7** according to the well-established synthetic protocol reported in the literature.[i] Compounds **8a-c**[ii] and **10**[iii] were synthesized following the reported procedures and were used in reactions with **6** or **7** to get the products **1a-c** and **3**.

[i] M. Ménand, I. Jabin, *Org. Lett.* **2009**, *11* (3), 673–676.

[ii] L. E. Bickerton, A. J. Sterling, P. D. Beer, F. Duarte, M. J. Langton, *Chem. Sci.* **2020**, *11* (18), 4722–4729.

[iii] S. J. Edwards, H. Valkenier,; N. Busschaert, P. A .Gale, A. P. Davis, *Angew. Chem. Int. Ed.* **2015**, *54* (15), 4592–4596.

Calix[6]arene **1a**:

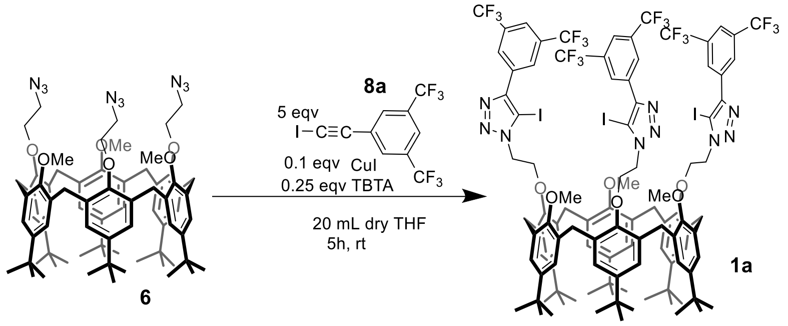

In a 25 mL round bottomed flask, CuI (7 mg, 32 µmol, 0.1 eqv) and tris(benzyltriazolylmethyl)amine (TBTA, 17 mg, 32 µmol, 0.1 eqv) were stirred in dry degassed THF (20mL). To this solution, 1-(iodoethynyl)-3,5-bis(trifluoromethyl)benzene (477 mg, 1.31 mmol, 4 eqv) and calix[6]arene trisazide **6** (400 mg, 327 µmol, 1 eqv) were added sequentially. This reaction mixture was stirred under argon atmosphere for 5 hours. After completion of the reaction, the solvent was removed under reduced pressure, and the residue was dissolved in DCM (20 mL). The organic mixture was washed with 25 % aqueous NH_4OH solution (2 x 15 mL) and dried over $MgSO_4$. The solvent was evaporated under reduced pressure to give the crude product, which was purified with column chromatography over silica gel with 1% MeOH/$CHCl_3$ as eluent to give **1a** as white solid (562 mg, 242 µmol, 74%). Rf: 0.40. (MeOH:$CHCl_3$ 1:99). Mp: 156 ºC. ^{1}H NMR (400 MHz, $CDCl_3$, 298 K) δ 0.71 (s, 27H, H_b), 1.35 (s, 27H, H_a), 2.06 (s, 9H, H_i), 3.16 (d, J = 15.1 Hz, 6H, $H_{d,eq}$), 4.01 (d, J = 15.0 Hz, 6H, $H_{d,ax}$), 4.36 (t, *J* = 5.5 Hz, 6H, H_e), 4.91 (t, *J* = 5.5 Hz, 6H, H_f), 6.58 (s, 6H, H_c), 7.17 (s, 6H, H_j), 7.92 (s, 3H, H_h), 8.30 (s, 6H, H_g).^{13}C NMR (101 MHz, $CDCl_3$, 298 K) δ 28.61 (C_d), 31.09 (C_b), 31.69 (C_a), 34.10 (C_p), 34.33 (C_m), 50.69 (C_f), 60.14 (C_i), 70.52 (C_e), 81.07 (C_u), 122.08 (C_h), 123.46 (q, *J* = 272 Hz, C_y), 123.69 (C_c), 128.09 (C_g), 128.31 (C_j), 131.90 (q, *J* = 34 Hz, C_x), 132.43 (C_o), 133.02 (C_w), 133.58 (C_s), 145.91 (C_n), 146.66 (C_q), 147.86 (C_v), 150.19 (C_t), 153.97 (C_r). FT-IR (cm^{-1}): 683, 897, 1009, 1132, 1184, 1278, 2362, 2959. HRMS (ESI) calculated for $C_{105}H_{108}F_{18}I_3N_9O_6Na^+$ [M+Na]$^+$: 2336.5152, found: 2336.5491.

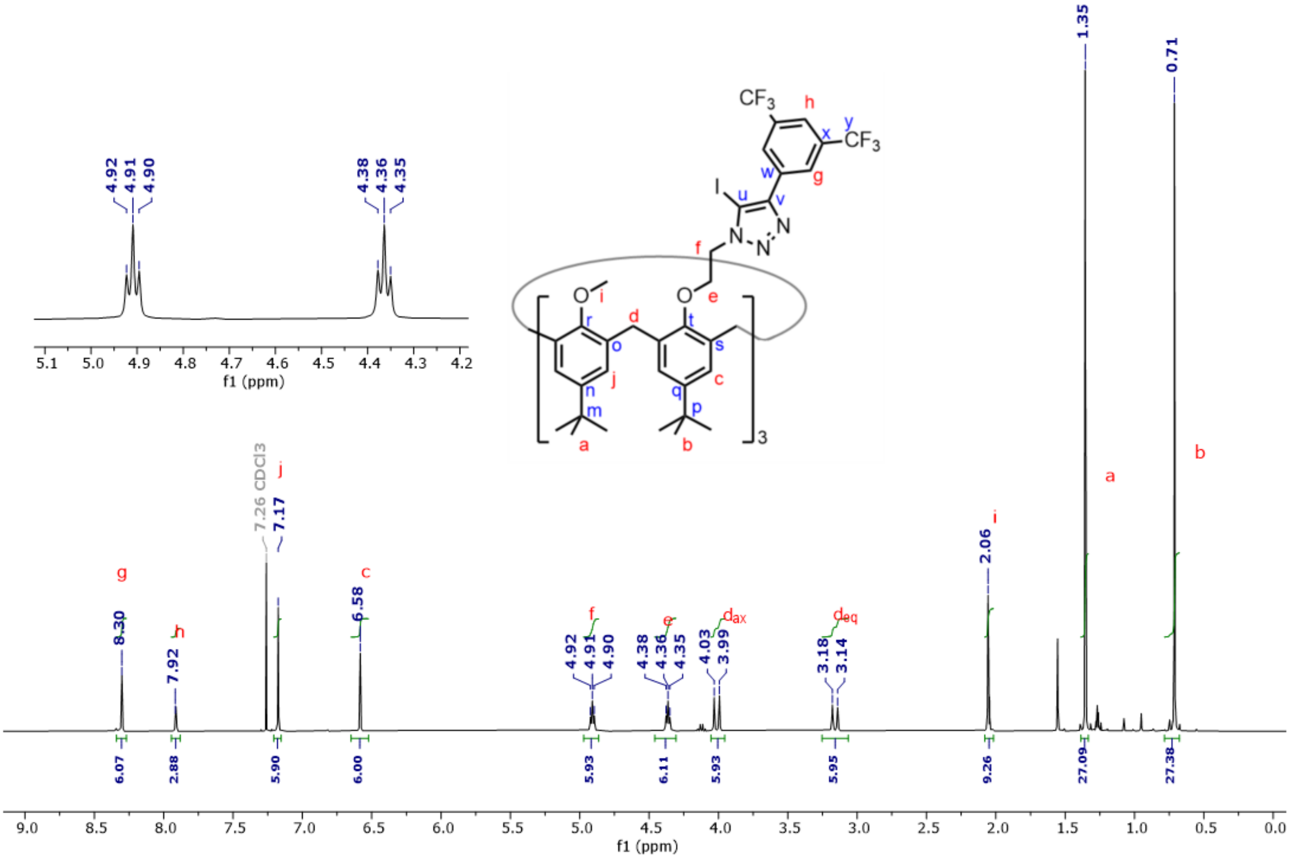

Figure S1 ^{1}H NMR spectrum (400 MHz, 298 K, $CDCl_3$) of calixarene **1a**

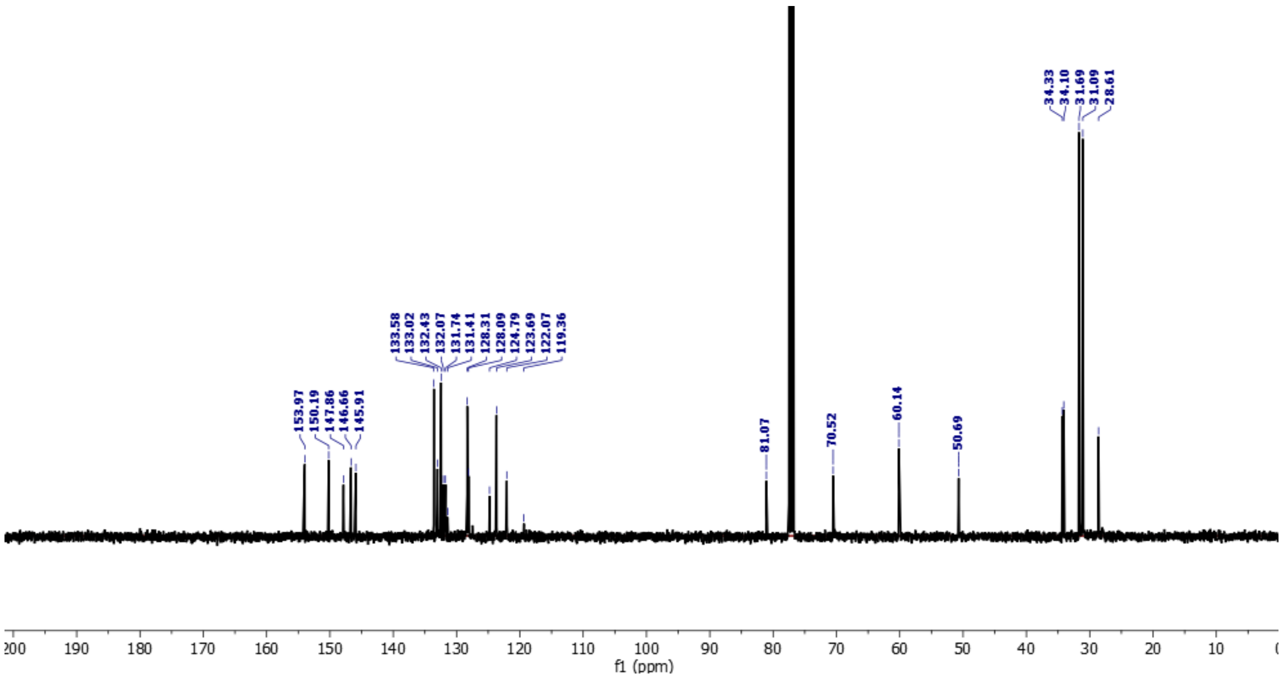

Figure S2 ^{13}C NMR spectrum (100 MHz, 298 K, $CDCl_3$) of calixarene **1a**

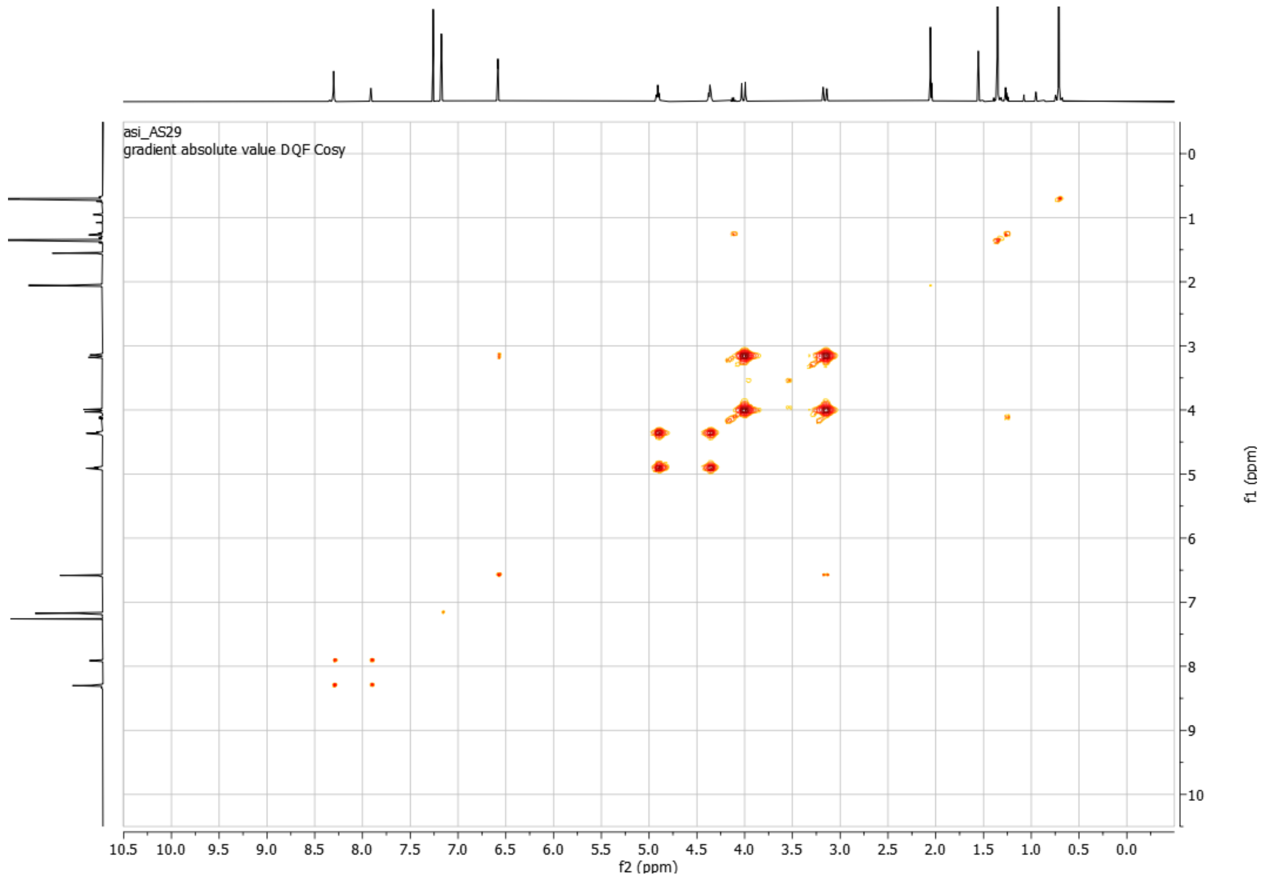

Figure S3 COSY spectrum (400 MHz, 298 K, $CDCl_3$) of calixarene **1a**

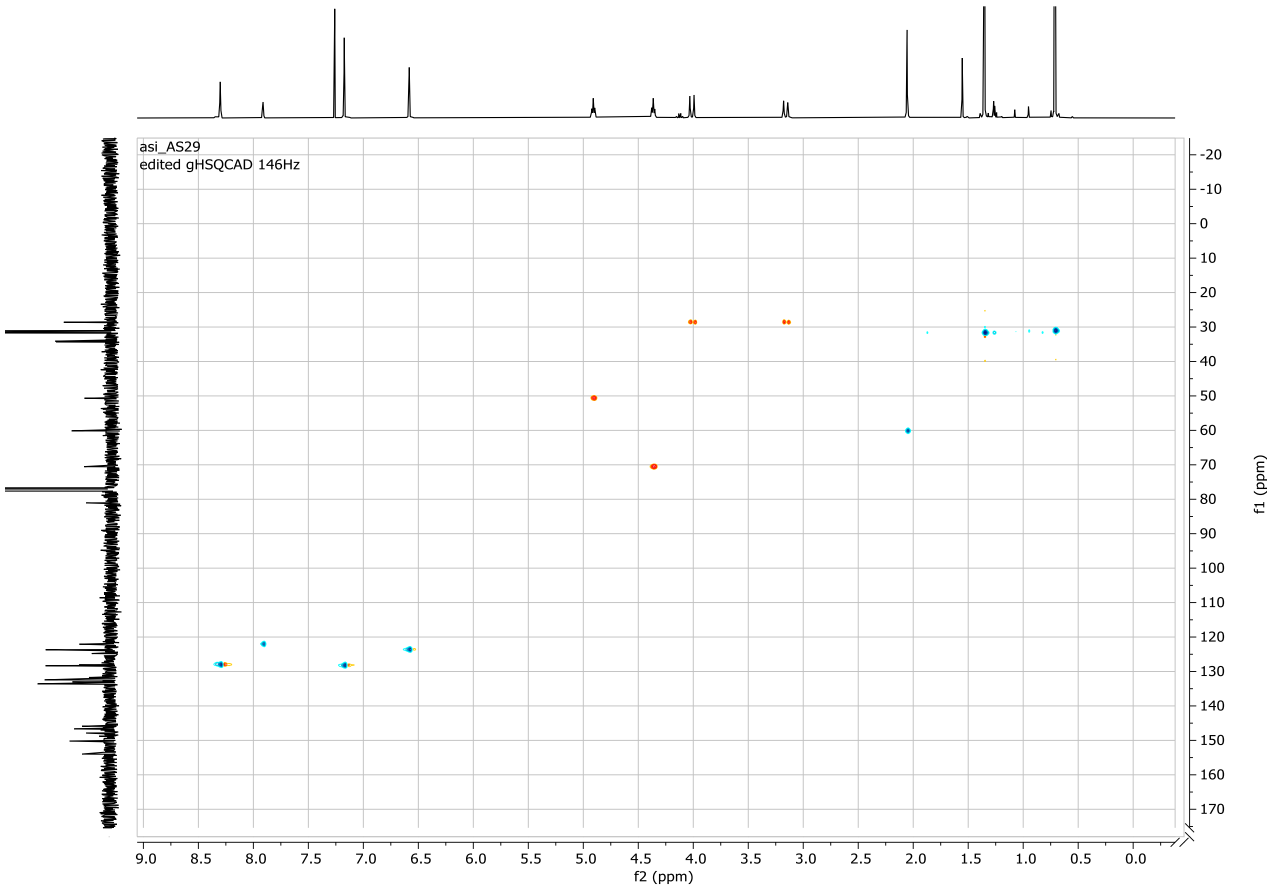

Figure S4 HSQC spectrum (400 MHz, 298 K, $CDCl_3$) of calixarene **1a**

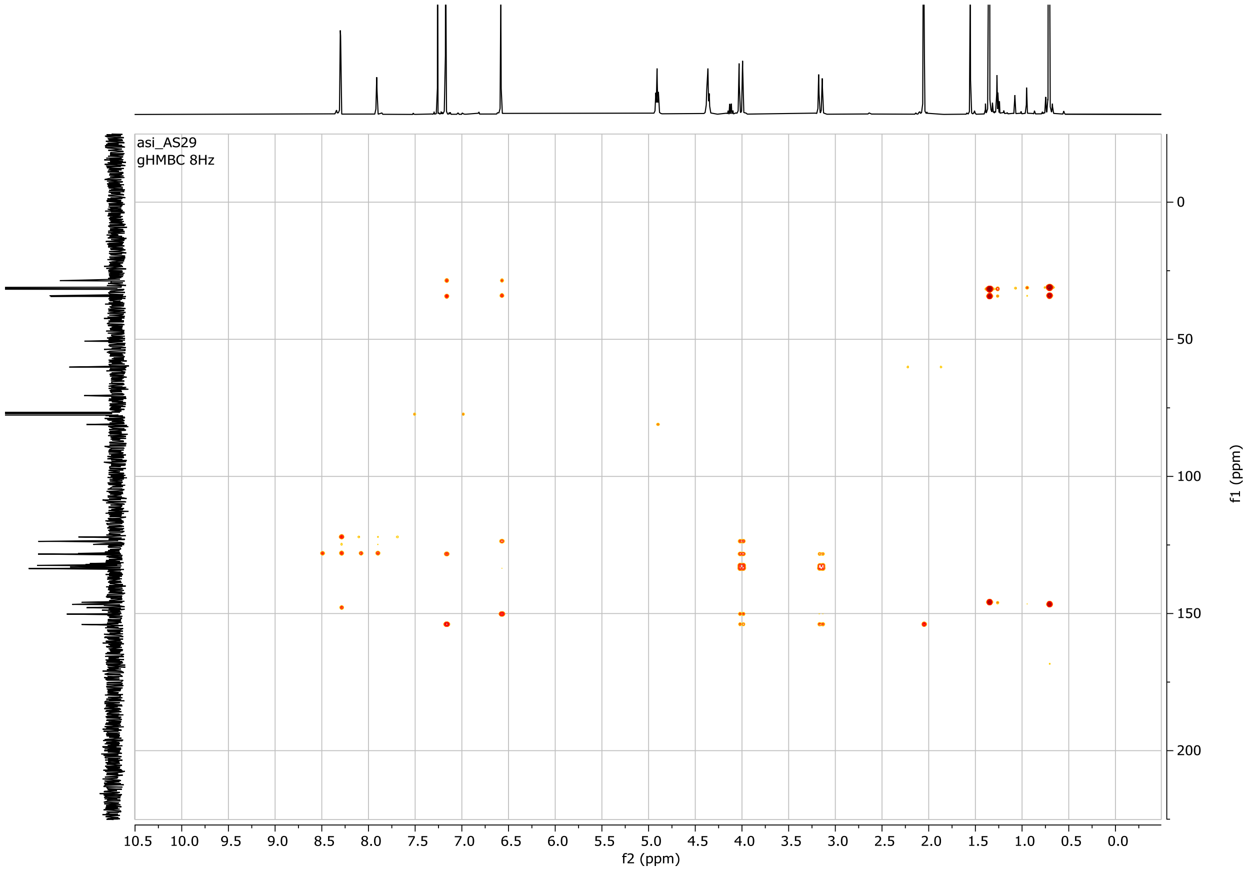

Figure S5 HMBC spectrum (400 MHz, 298 K, $CDCl_3$) of calixarene **1a**

Calix[6]arene **1b**:

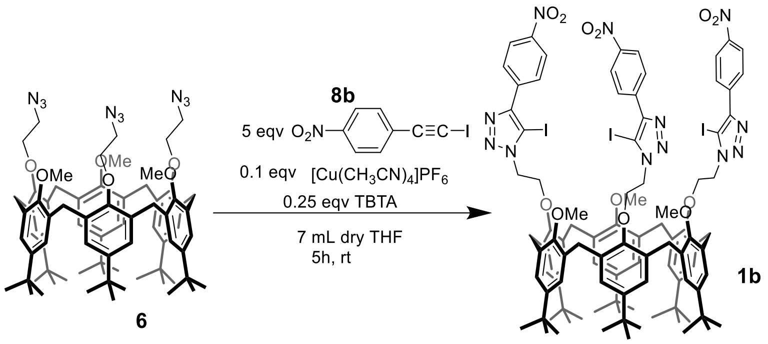

In a 25 mL round bottomed flask, $[Cu(CH_3CN)_4]PF_6$ (11 mg, 30.7 µmol, 0.25 eqv) and TBTA (16 mg, 30.7 µmol, 0.25 eqv) were stirred in dry and degassed THF (7 mL). To this solution, 1-(iodoethynyl)-4-nitrobenzene (167 mg, 613 µmol, 5 eqv) and calix[6]arene trisazide **6** (150 mg, 121 µmol, 1 eqv) were added sequentially. This reaction mixture was stirred under argon atmosphere for 5 hours. After completion of the reaction, the solvent was removed in vacuo, and the residue was dissolved in chloroform (20 mL). The organic mixture was washed with 10% aqueous NH_4OH solution (2 x 10 mL), followed by brine (20 mL) and then dried over $MgSO_4$. The solvent was evaporated under reduced pressure to give the crude product. The residue was purified by silica gel flash chromatography with 1% MeOH/DCM mixture as eluent to obtain **1b** as brownish orange solid (196 mg, 96 µmol, 78%). Rf: 0.28. (MeOH:CH_2Cl_2 1:99). Mp: 161 °C. ^{1}H NMR (400 MHz, $CDCl_3$, 298 K) δ 0.70 (s, 27H, H_b), 1.36 (s, 27H, H_a), 1.98 (s, 9H, H_i), 3.13 (d, J = 15.1 Hz, 6H, $H_{d,eq}$), 3.87 (d, J = 15.0 Hz, 6H, $H_{d,ax}$), 4.34 (t, J = 5.4 Hz, 6H, H_e), 4.98 (t, J = 5.4 Hz, 6H, H_f), 6.55 (s, 6H, H_c), 7.17 (s, 6H, H_j), 7.98 (d, J = 8.9 Hz, 6H, H_g), 8.31 (d, J = 8.9 Hz, 6H, H_k).^{13}C NMR (101 MHz, $CDCl_3$, 298 K) δ 28.57 (C_d), 31.06 (C_b), 31.74 (C_a), 34.09 (C_p), 34.37 (C_m), 50.64 (C_f), 60.29 (C_i), 71.10 (C_e), 82.03 (C_u), 123.71(C_c + C_k), 128.35 (C_j), 129.12 (C_g), 132.43 (C_o), 133.50 (C_s), 137.42 (C_w), 146.11 (C_n), 146.77 (C_q), 147.62 (C_x), 148.49 (C_v), 150.13 (C_t), 153.88(C_r). FT-IR (cm^{-1}): 683, 853, 1013, 1108, 1188, 1340, 1481, 1519, 2362, 2963. HRMS (ESI) Calculated for $C_{99}H_{112}I_3N_{12}O_{12}^+$ $[M+H]^+$: 2042.5685, found: 2042.5458.

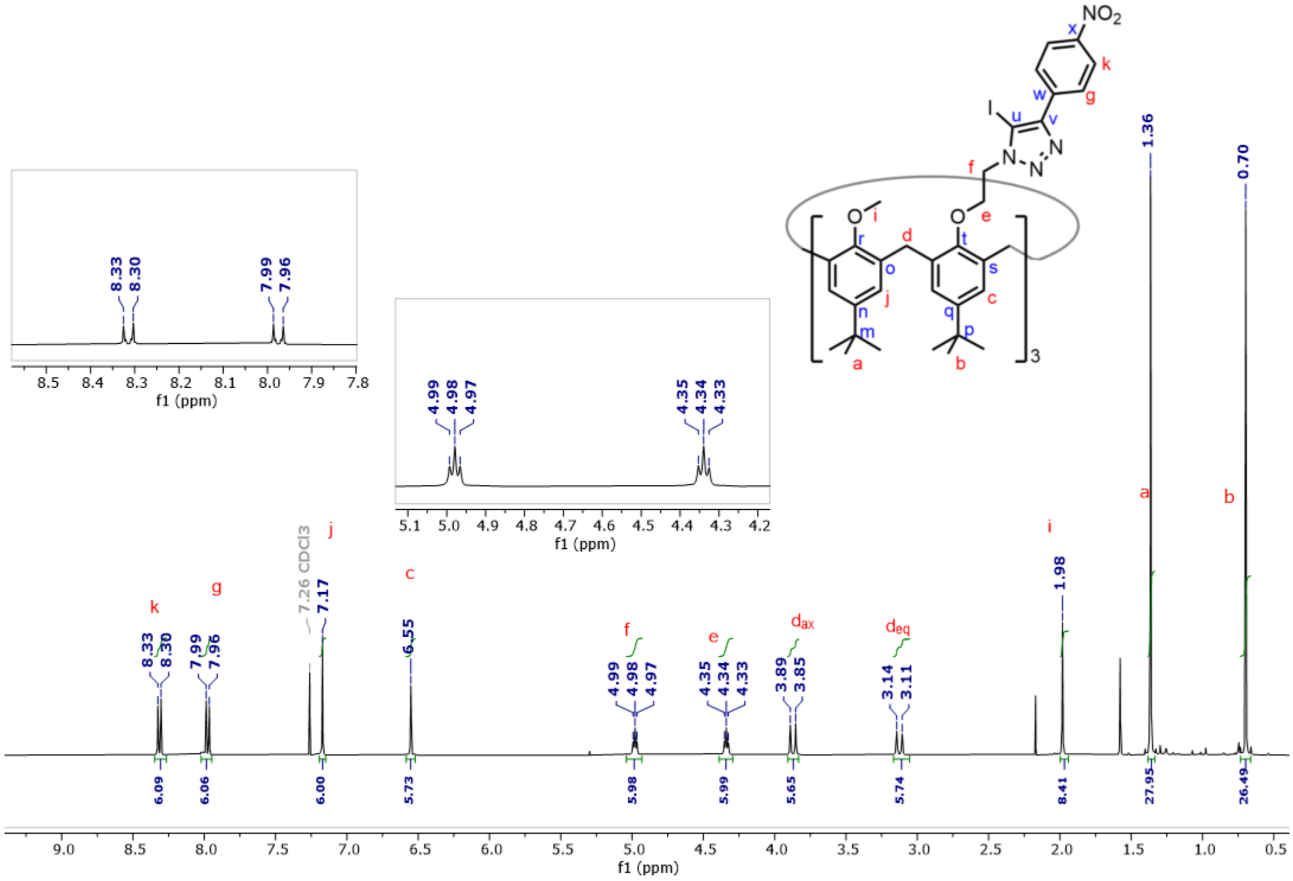

Figure S6 ^{1}H NMR spectrum (400 MHz, 298 K, $CDCl_3$) of calixarene **1b**

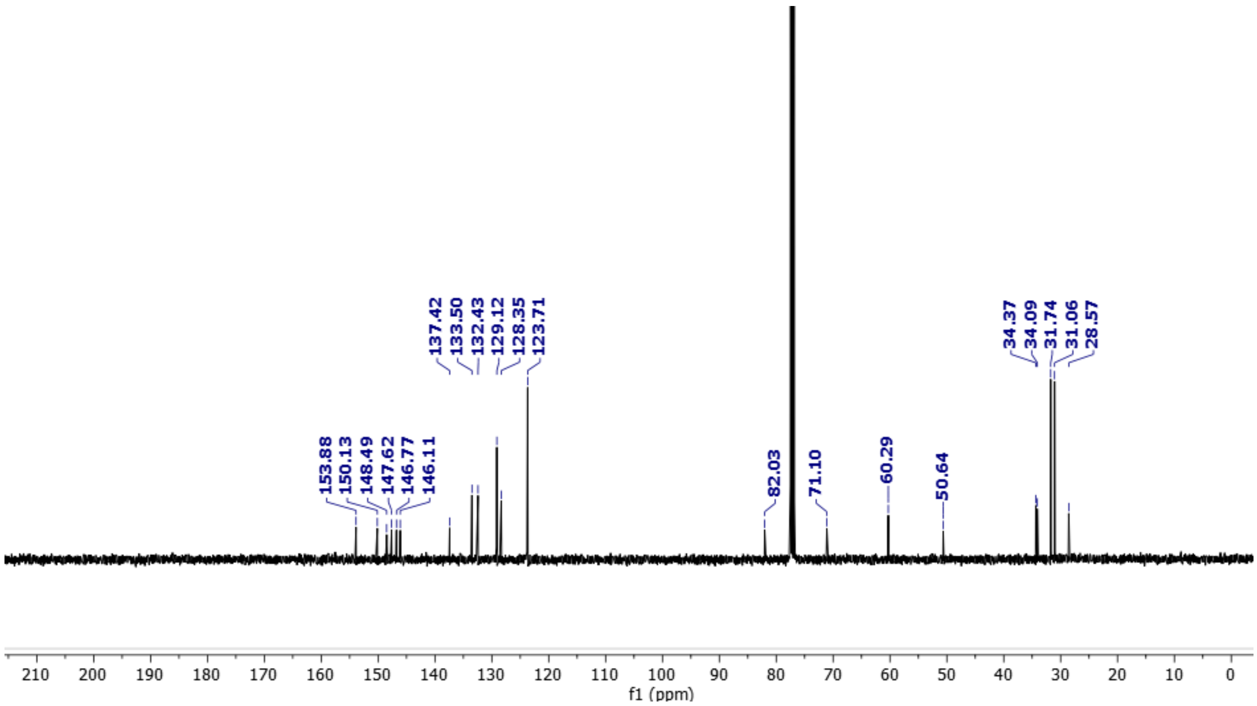

Figure S7 ^{13}C NMR spectrum (100 MHz, 298 K, $CDCl_3$) of calixarene **1b**

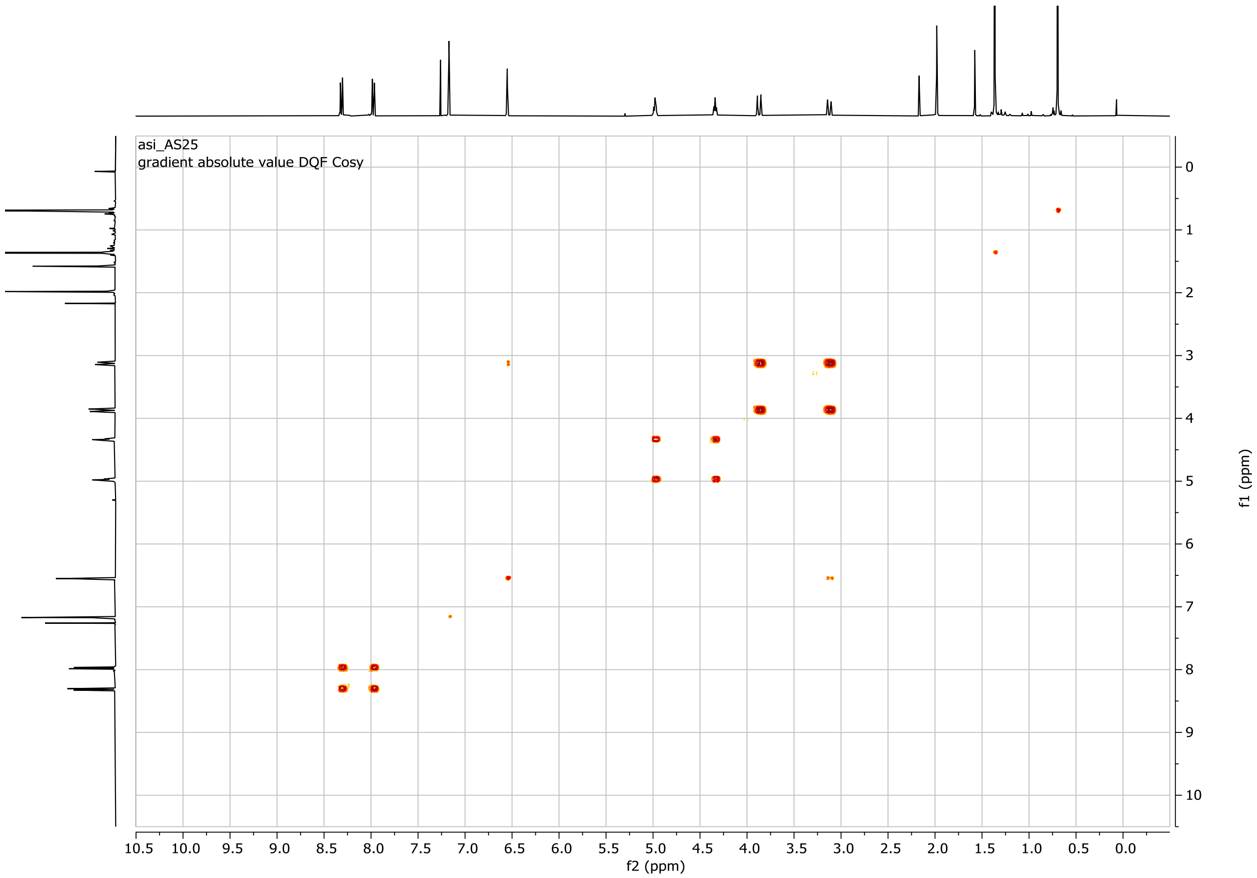

Figure S8 COSY spectrum (400 MHz, 298 K, $CDCl_3$) of calixarene **1b**

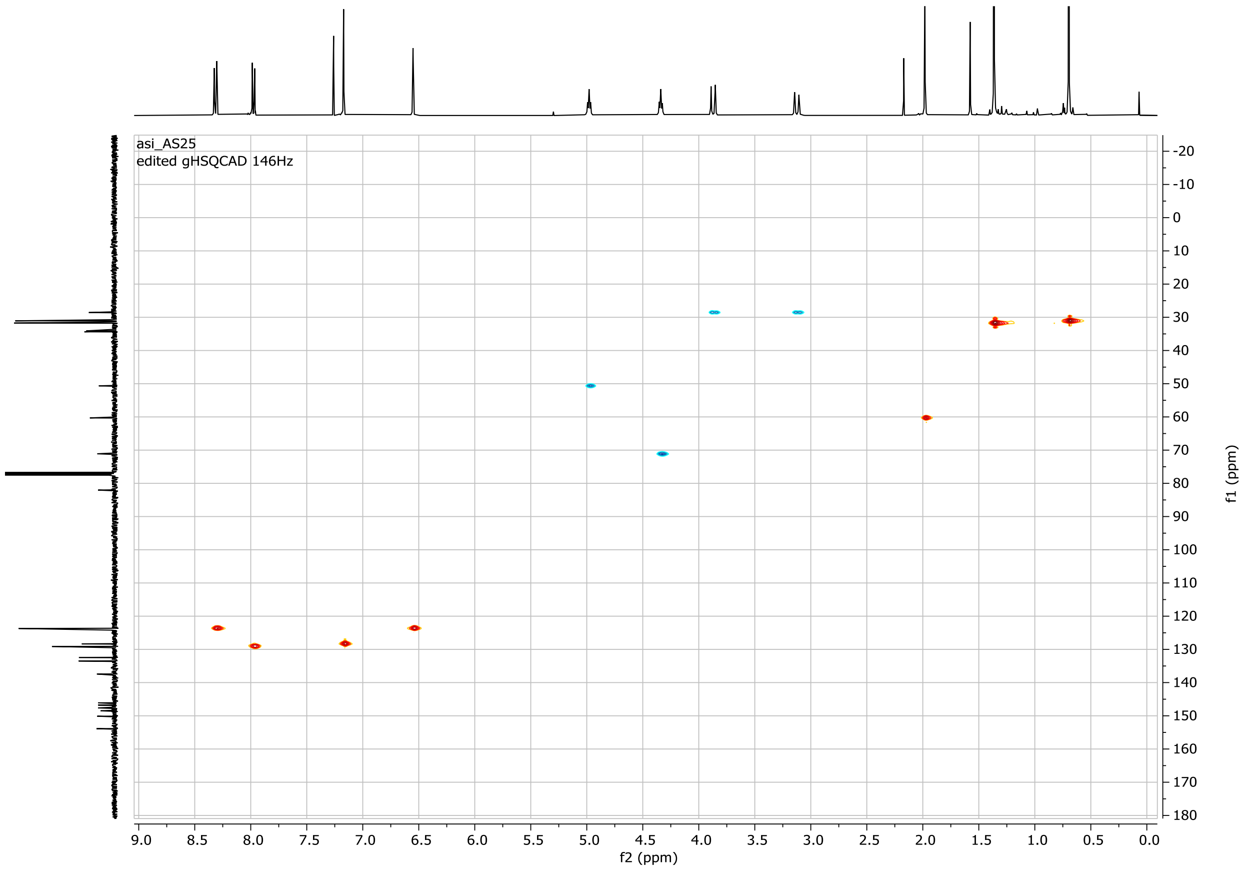

Figure S9 HSQC spectrum (400 MHz, 298 K, $CDCl_3$) of calixarene **1b**

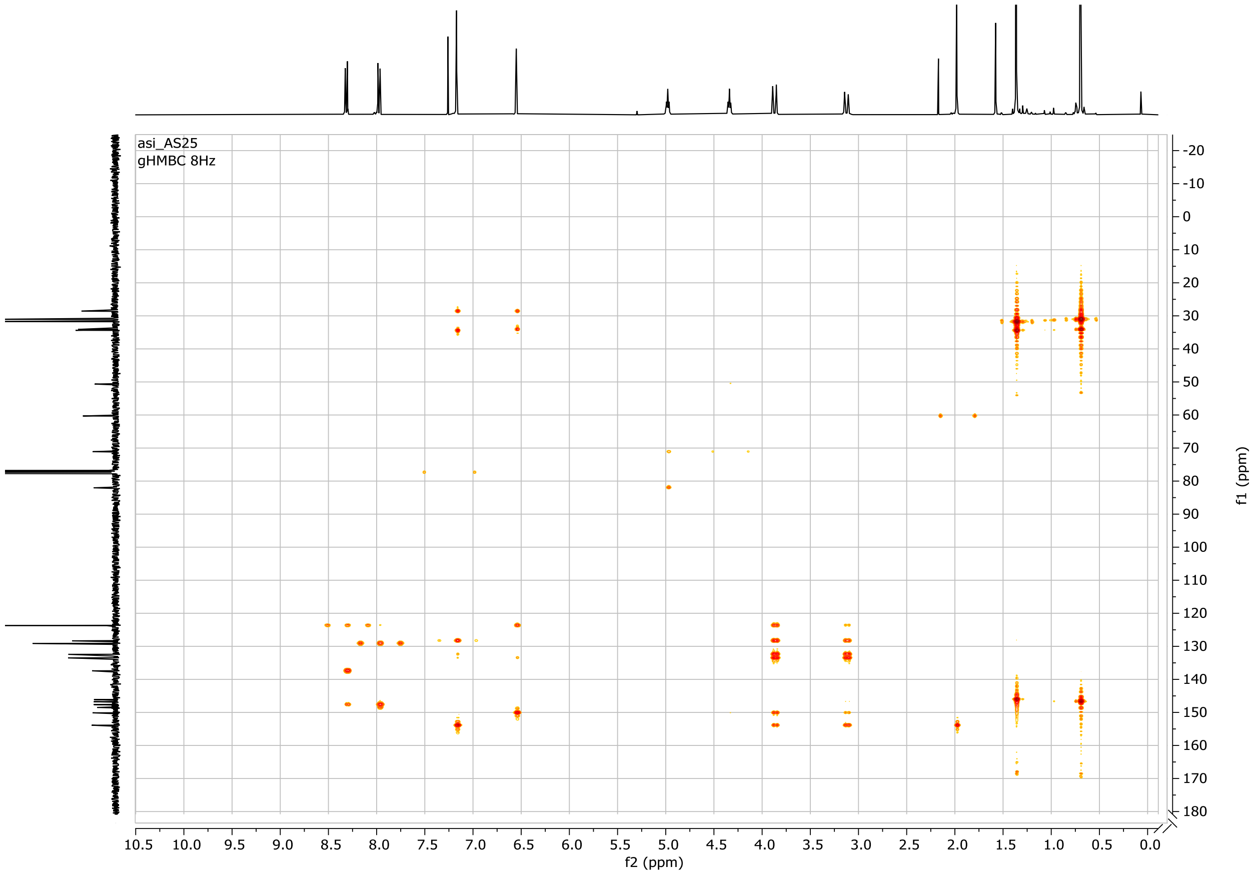

Figure S10 HMBC spectrum (400 MHz, 298 K, $CDCl_3$) of calixarene **1b**

Calix[6]arene **1c**:

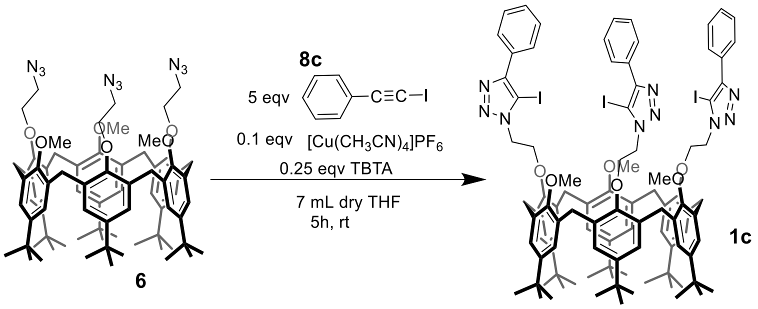

In a 25 mL round bottomed flask, $[Cu(CH_3CN)_4]PF_6$ (8 mg, 20.5 µmol, 0.25 eqv) and TBTA (12 mg, 20.5 µmol, 0.25 eqv) were stirred in dry degassed THF (7 mL). To this solution, 1-iodoethynylbenzene (100 mg, 410 µmol, 5 eqv) and calix[6]arene trisazide **6** (100 mg, 81 µmol, 1 eqv) were added sequentially. This reaction mixture was stirred under argon atmosphere for 5 hours. After completion of the reaction, the solvent was removed in vacuo, and the residue was dissolved in DCM (20 mL). The organic mixture was washed with 25 % aqueous NH_4OH solution (2 x 10 mL), followed by washing with brine (20 mL) and drying of the combined organic layers over $MgSO_4$. The solvent was evaporated to dryness to give the crude product, which was washed with pentane to remove most of the excess 1-iodoethynylbenzene present. Finally, this crude product was purified by column chromatography over silica gel with EtOAc/cyclohexane (20:80) as eluent to give **1c** as white solid (108 mg, 57 µmol, 70%). Mp: 195 °C. ^1H NMR (400 MHz, $CDCl_3$, 298 K) δ 0.73 (s, 27H, H_b), 1.37 (s, 27H, H_a), 2.08 (s, 9H, H_i), 3.20 (d, J = 15.2 Hz, 6H, $H_{d,eq}$), 4.10 (d, J = 15.1 Hz, 6H, $H_{d,ax}$), 4.38 (t, J = 5.7 Hz, 6H, H_e), 4.90 (t, J = 5.7 Hz, 6H, H_f), 6.60 (s, 6H, H_c), 7.18 (s, 6H, H_j), 7.38 – 7.49 (m, 9H, H_h + H_k), 7.76 (dd, J = 7.7, 1.9 Hz, 6H, H_g). ^{13}C NMR (101 MHz, $CDCl_3$, 298 K) δ 29.00 (C_d), 31.12 (C_b), 31.76 (C_a), 34.09 (C_p), 34.34 (C_m), 50.47 (C_f), 60.22 (C_i), 70.73 (C_e), 80.68 (C_u), 123.70 (C_c), 128.24 (C_j), 128.40 (C_h), 128.44 (C_k), 128.54 (C_g), 130.94 (C_w), 132.67 (C_o), 133.75 (C_s), 145.70 (C_n), 146.49 (C_q), 150.40 (C_t), 150.60 (C_v), 154.24 (C_r). FT-IR (cm^{-1}): 695, 768, 877, 1012, 1114, 1201, 1293, 1361, 1481, 2350, 2957. HRMS (ESI) Calculated for $C_{99}H_{114}I_3N_9O_6Na^+$ $[M+Na]^+$: 1929.5952, found: 1929.5922.

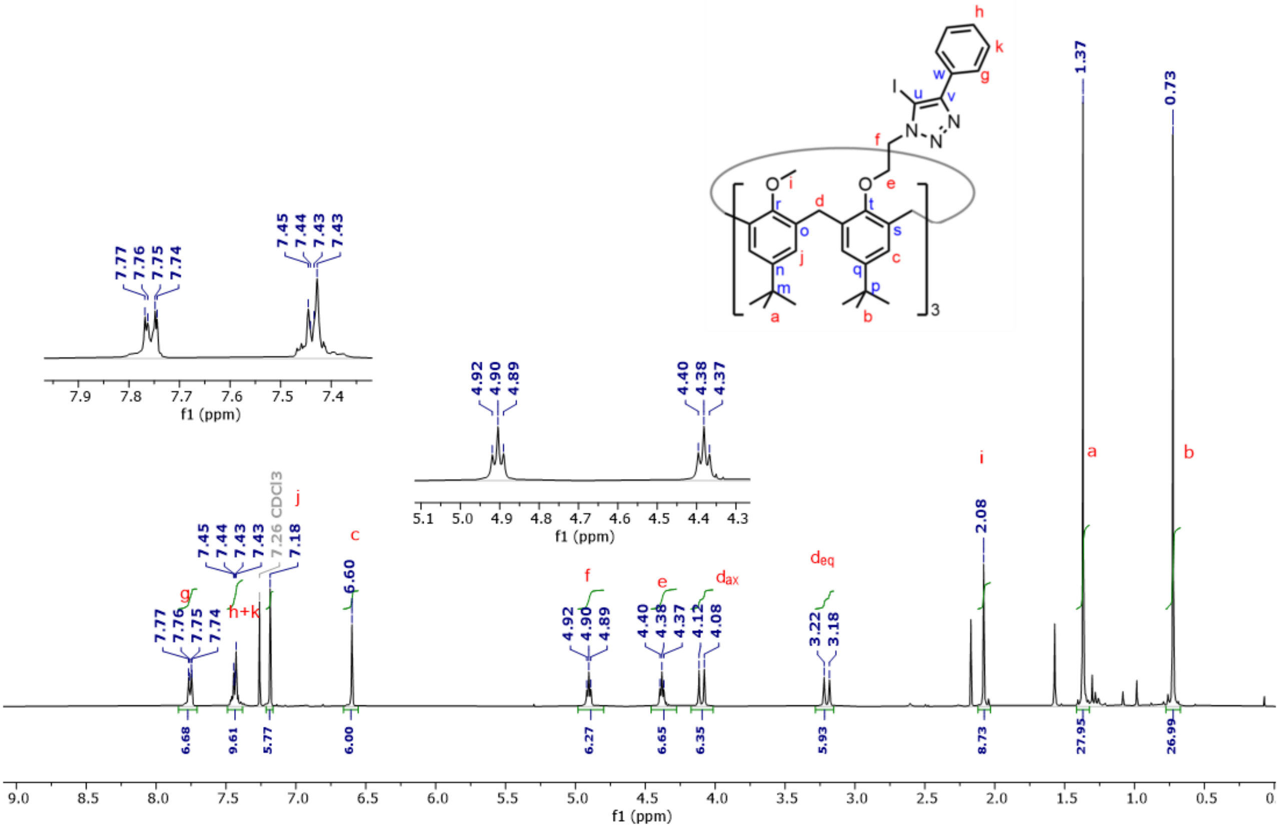

Figure S11 ^1H NMR spectrum (400 MHz, 298 K, $CDCl_3$) of calixarene **1c**

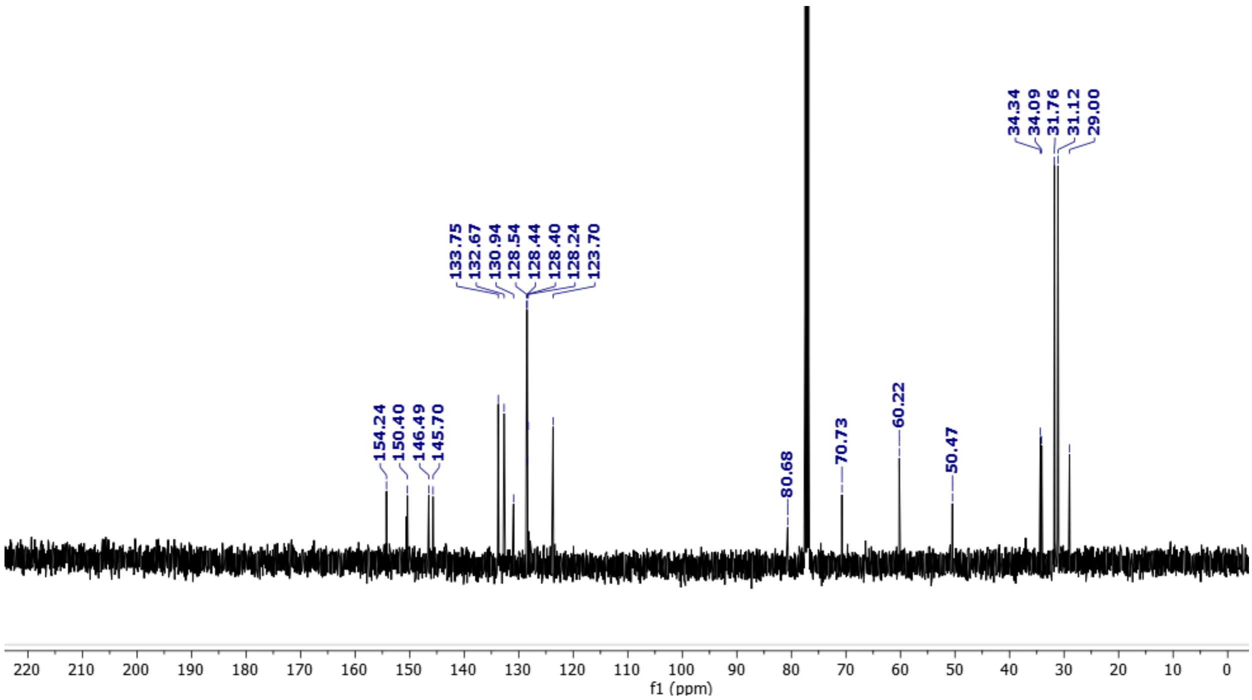

Figure S12 ^{13}C NMR spectrum (100 MHz, 298 K, $CDCl_3$) of calixarene **1c**

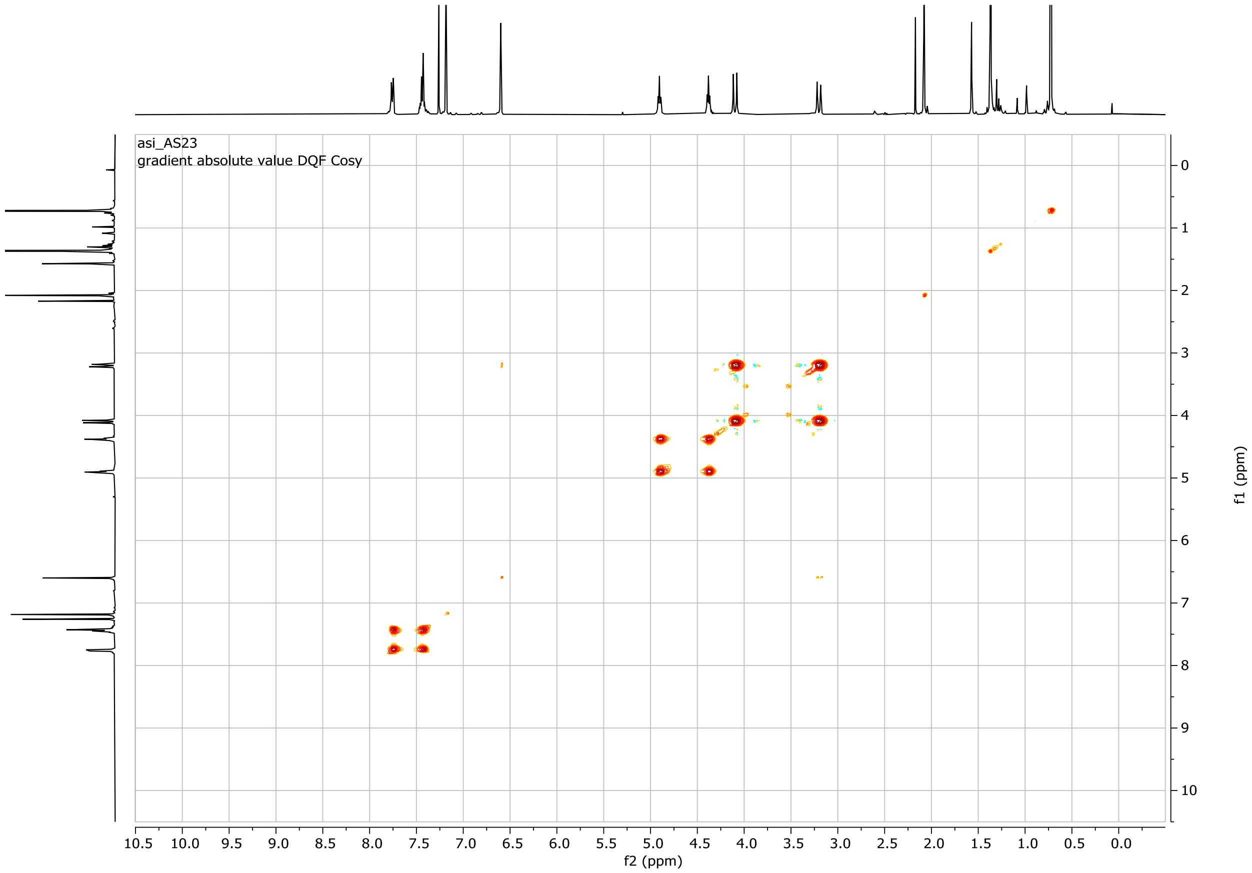

Figure S13 COSY spectrum (400 MHz, 298 K, $CDCl_3$) of calixarene **1c**

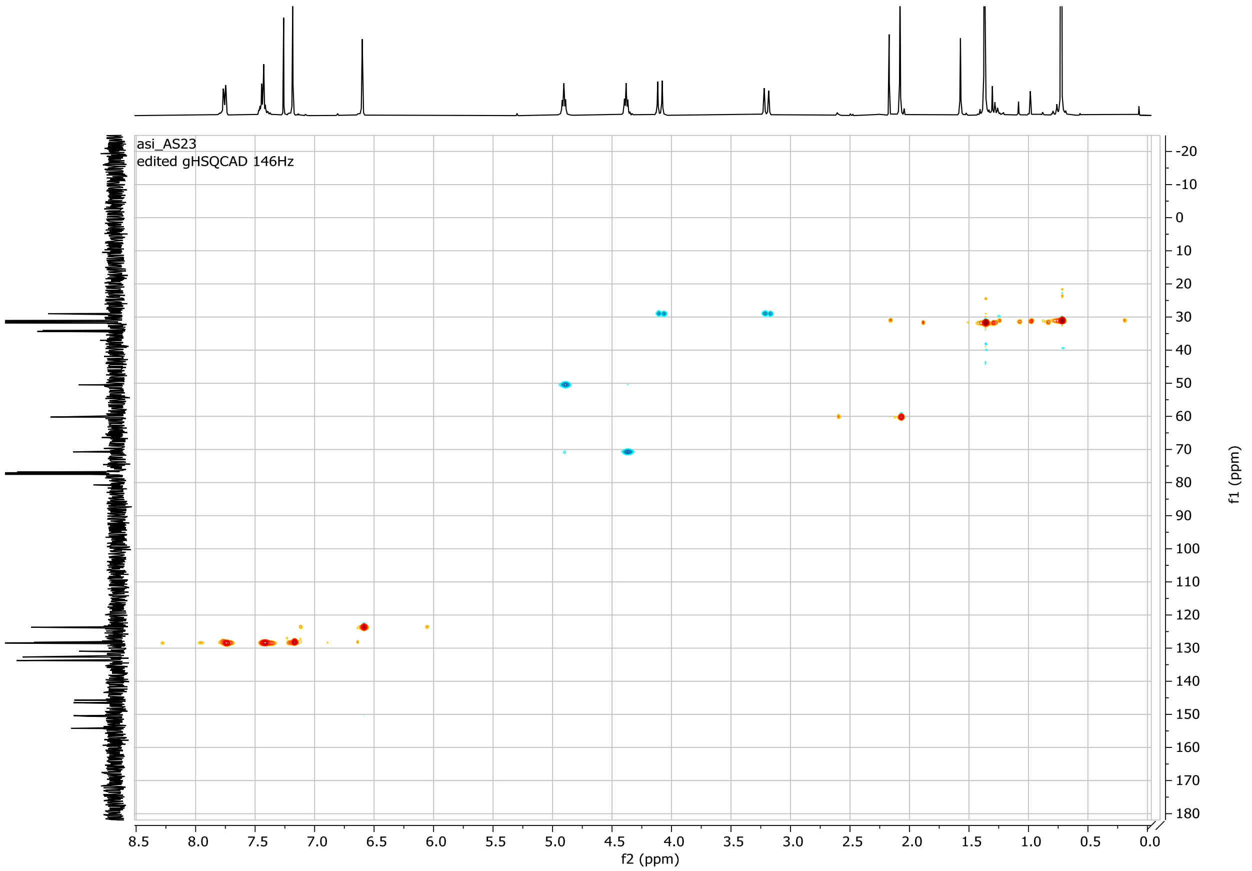

Figure S14 HSQC spectrum (400 MHz, 298 K, $CDCl_3$) of calixarene **1c**

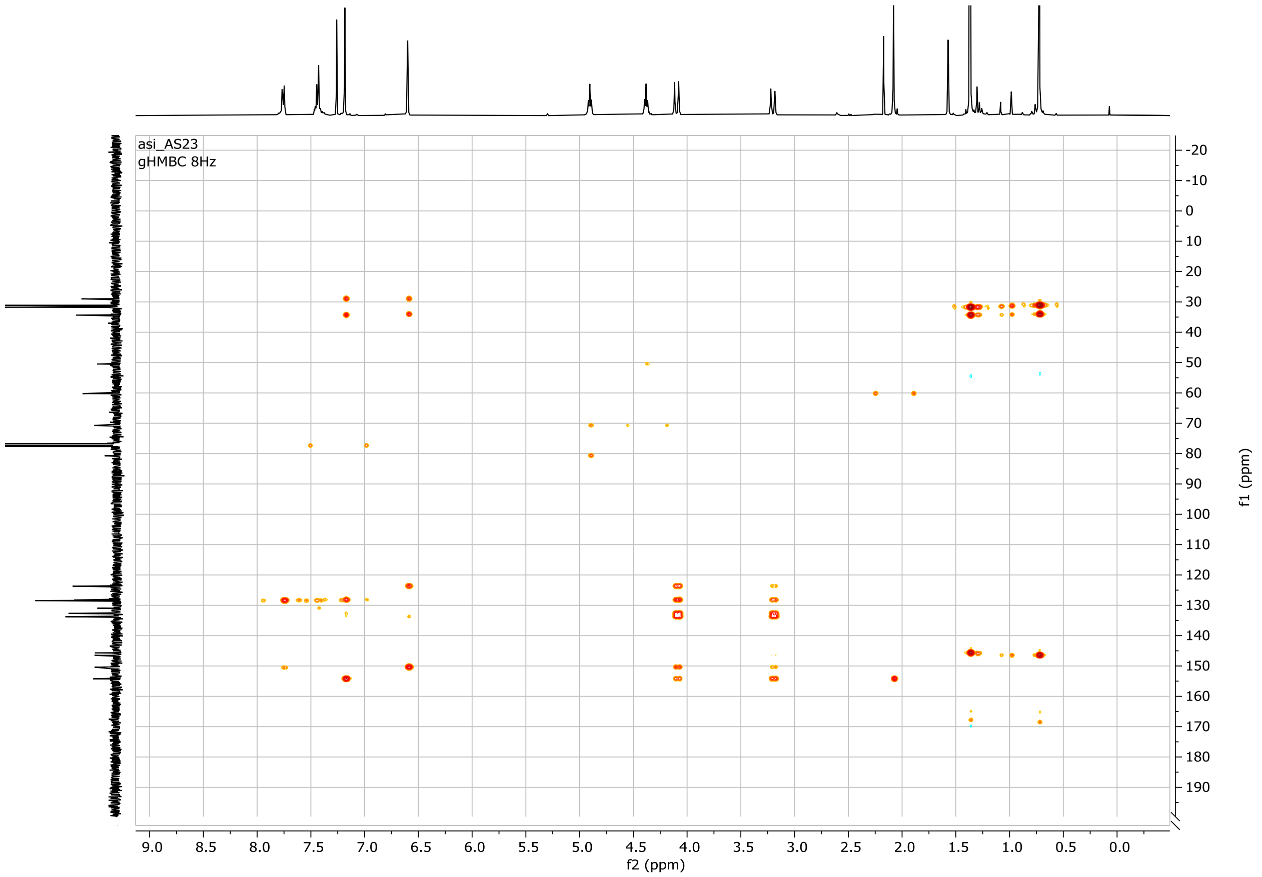

Figure S15 HMBC spectrum (400 MHz, 298 K, $CDCl_3$) of calixarene **1c**

Calix[6]arene **2**:

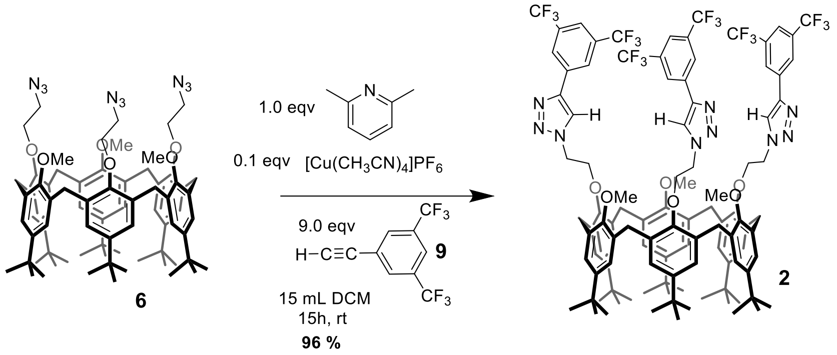

In a 25 mL round bottomed flask, to a solution of calix[6]arene trisazide **6** (300 mg, 245 µmol, 1 eqv) in DCM (15 mL) under Ar atmosphere, was added 3,5-bis(trifluoromethyl)phenyl acetylene (389 µL, 2.2 mmol, 9 eqv). To this solution 2,6-lutidine (28 µL, 245 µmol, 1 eqv) and $[Cu(CH_3CN)_4]PF_6$ (9 mg, 24 µmol, 0.1 eqv) were added sequentially and the reaction mixture was stirred at room temperature for 15 h. Subsequently, the reaction mixture was diluted with DCM (75 mL) and aqueous NaOH (1 M, 75 mL) was added. The layers were separated, and the aqueous layer was extracted twice with DCM (2×30 mL). The combined organic layers were washed with brine (40 mL), dried over $MgSO_4$, and evaporated to dryness. Finally, the crude solid was purified using column chromatography over silica gel with EtOAc/cyclohexane (20:80) as eluent to give **2** as white solid (460 mg, 238 µmol, 97%). Rf: 0.33. EtOAc/cyclohexane (20:80). Mp: 147 °C. ^{1}H NMR (400 MHz, $CDCl_3$, 298 K) δ 0.78 (s, 27H, H_b), 1.31 (s, 27H, H_a), 2.31 (s, 9H, H_i), 3.26 (d, J = 15.1 Hz, 6H, $H_{d,eq}$), 4.25 – 4.30 (m, 12H, $H_{d,ax}$ + H_e), 4.92 (t, J = 4.9 Hz, 6H, H_f), 6.63 (s, 6H, H_c), 7.14 (s, 6H, H_j), 7.78 (s, 3H, H_h), 8.26 (s, 6H, H_g), 8.42 (s, 3H, H_k). ^{13}C NMR (101 MHz, $CDCl_3$, 298 K) δ 29.19 (C_d), 31.21 (C_b), 31.63 (C_a), 34.15 (C_p), 34.32 (C_m), 51.01 (C_f), 60.24 (C_i), 70.91 (C_e), 121.48 (C_h), 122.77 (C_k), 123.15 (q, J = 272 Hz, C_y), 124.18 (C_c), 125.96 (C_g), 128.15 (C_j), 132.07 (q, J = 33 Hz, C_x), 132.54 (C_o), 133.22 (C_w), 133.47 (C_s), 145.58 (C_v), 146.29 (C_n), 146.95 (C_q), 150.77 (C_t), 154.07 (C_r). FT-IR (cm^{-1}): 684, 897, 1135, 1180, 1279, 1361, 1481, 2953. HRMS (ESI) Calculated for $C_{105}H_{111}F_{18}N_9O_6K^+$ $[M+K]^+$: 1975.8036, found: 1975.8007.

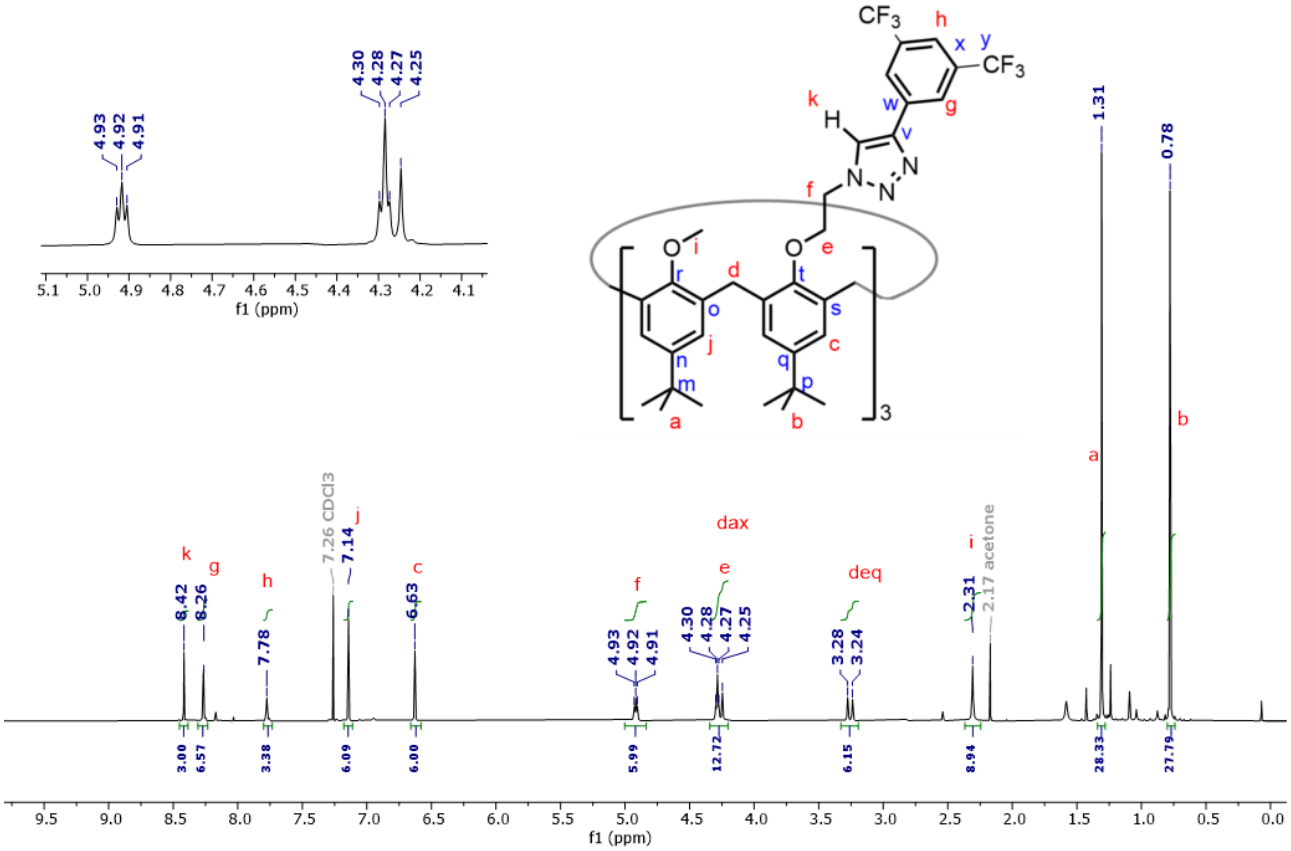

Figure S16 ^{1}H NMR spectrum (400 MHz, 298 K, $CDCl_3$) of calixarene **2**

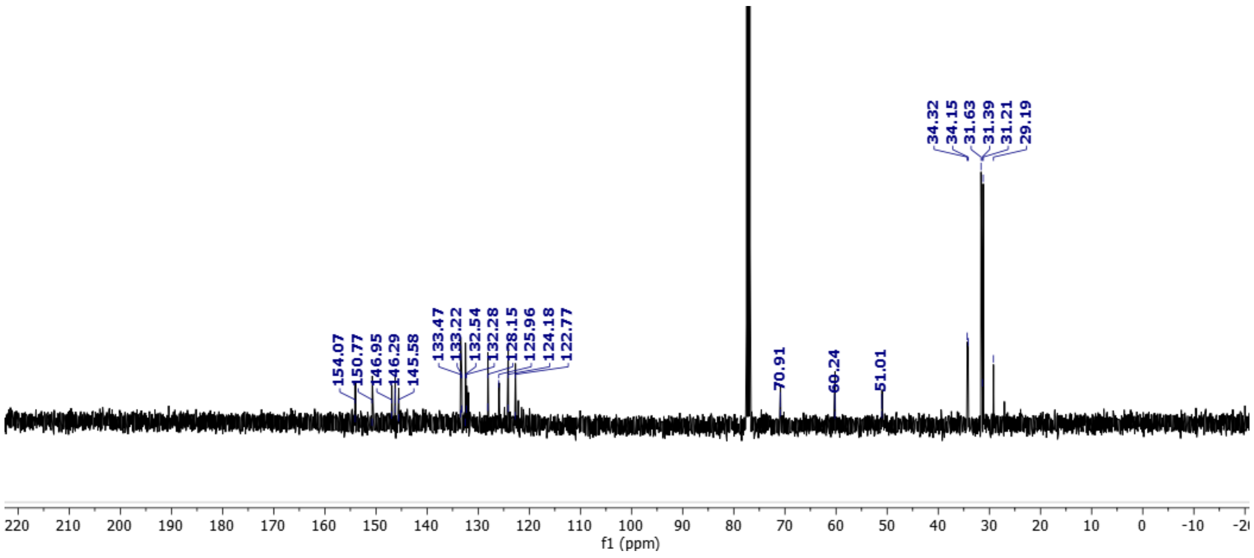

Figure S17 ^{13}C NMR spectrum (100 MHz, 298 K, $CDCl_3$) of calixarene **2**

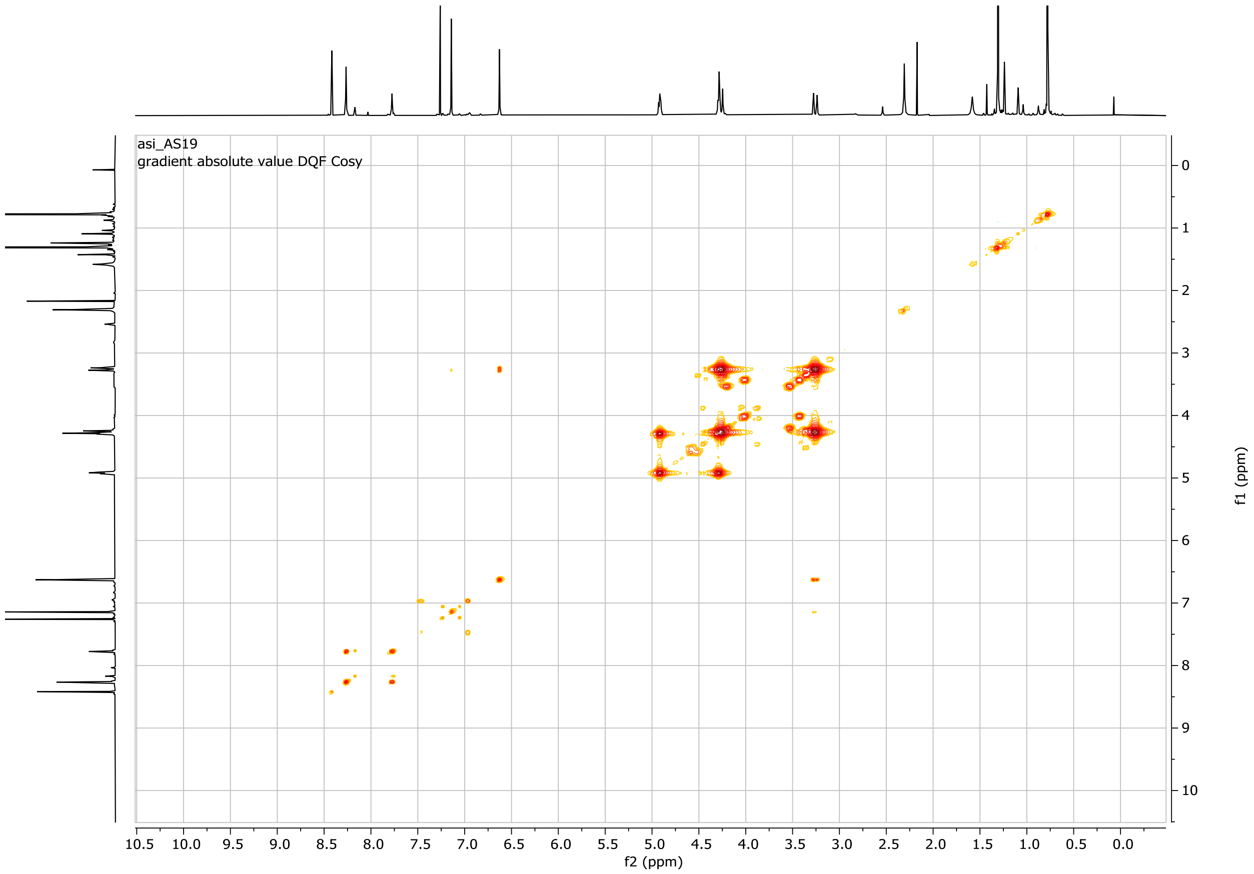

Figure S18 COSY spectrum (400 MHz, 298 K, $CDCl_3$) of calixarene **2**

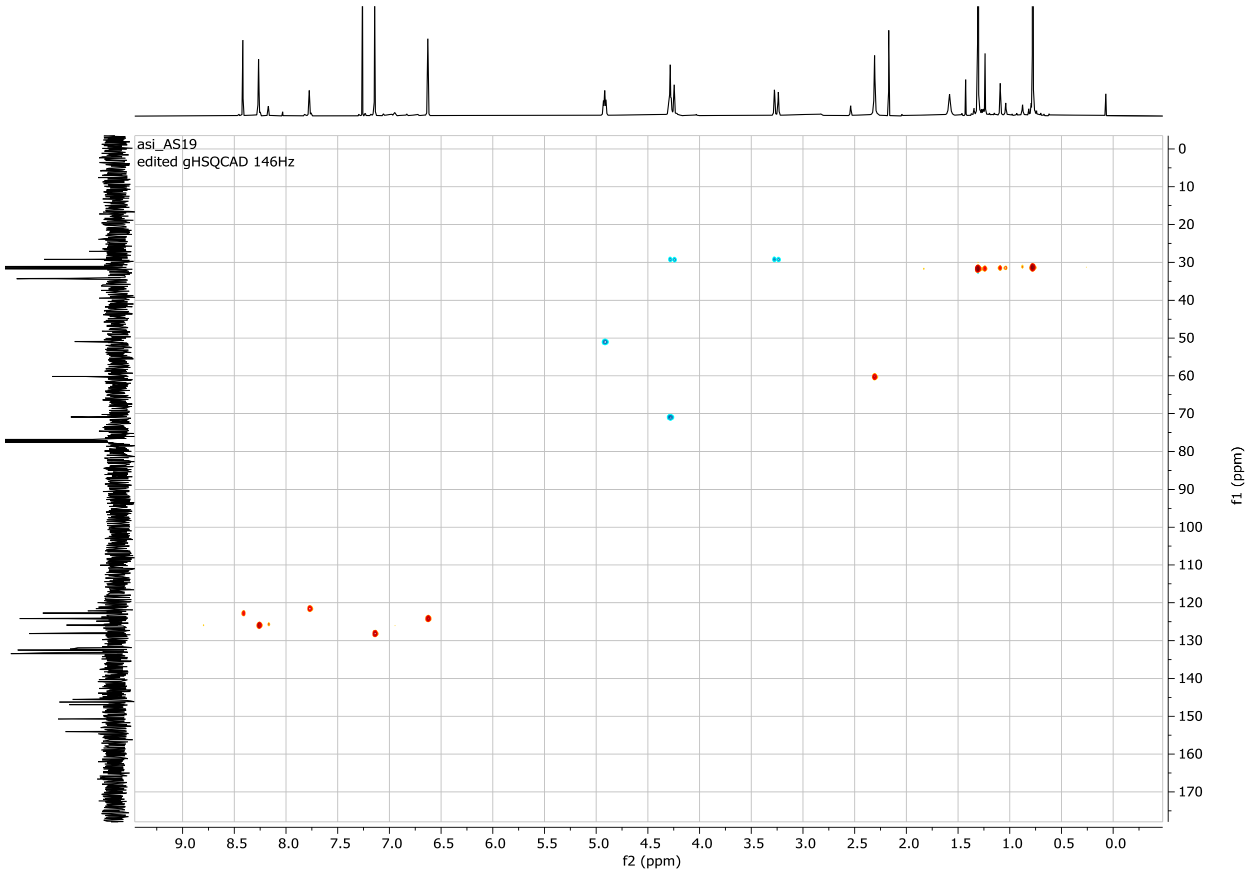

Figure S19 HSQC spectrum (400 MHz, 298 K, $CDCl_3$) of calixarene **2**

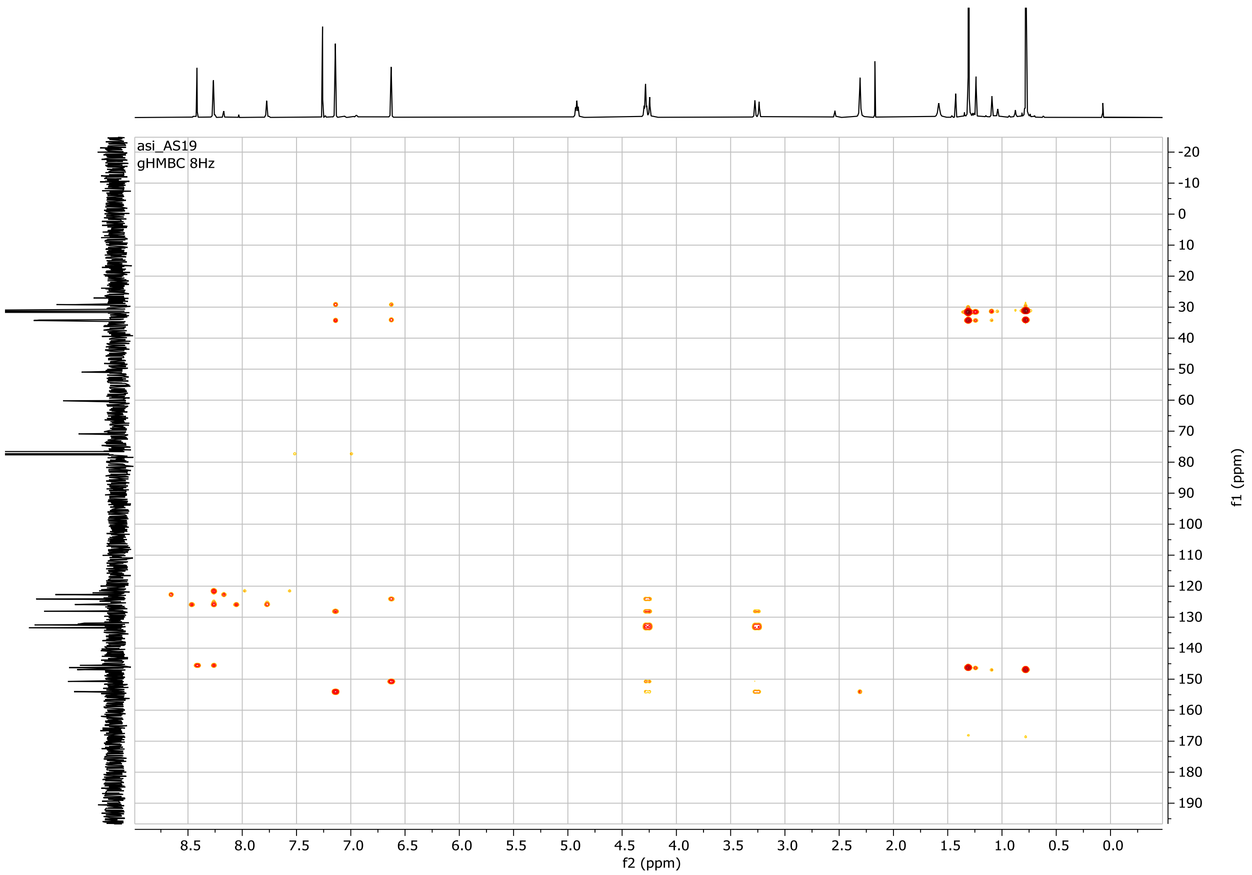

Figure S20 HMBC spectrum (400 MHz, 298 K, $CDCl_3$) of calixarene **2**

Calix[6]arene **3**:

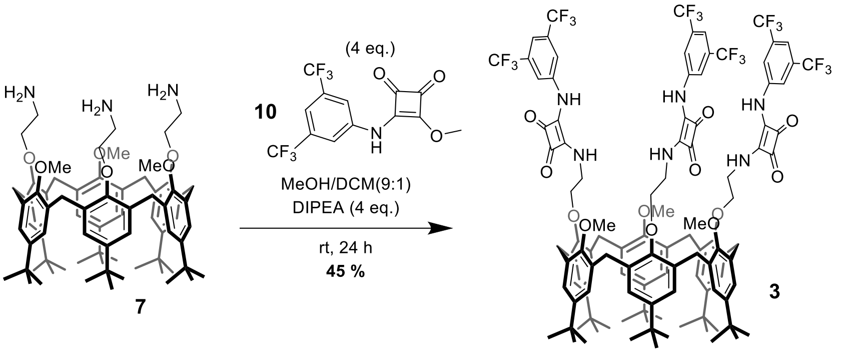

In a 25 mL round bottomed flask, to a solution of calix[6]arene trisamine **7** (100 mg, 87 µmol, 1 eqv), first dissolved in anhydrous DCM (1 mL) and then in anhydrous MeOH (9 mL), was added **10** (118 mg, 349 µmol, 4 eqv) and DIPEA (60 uL, 349 µmol, 4 eqv). The resulting suspension was stirred at room temperature under argon for 24 hours. The reaction mixture was concentrated under reduced pressure and the obtained residue was washed with small volumes of MeOH (1 mL, 700 µL, 500 µL and 500 µL) and centrifuged, yielding **3** as pale-yellow solid (80 mg, 39 µmol, 45%). Mp: (decomposes after 250ºC). ^{1}H NMR (400 MHz, DMSO-d$_6$) δ 0.67 (s, 27H, H$_b$), 1.25 (s, 27H, H$_a$), 1.95 (s, 9H, H$_i$), 3.36 (d, 6H, H$_{d,eq}$), 4.07 (s, 12H, H$_e$ + H$_f$), 4.36 (d, *J* = 15.1 Hz, 6H, H$_{d,ax}$), 6.51 (s, 6H, H$_c$), 7.19 (s, 6H, H$_{c1}$), 7.54 (s, 3H, H$_h$), 7.88 (s, 6H, H$_g$), 8.06[iv] (s, 3H, H$_k$), 10.18[iv] (s, 3H, H$_j$). ^{13}C NMR (101 MHz, DMSO-d$_6$) δ 28.82 (C$_d$), 30.62 (C$_b$), 31.12 (C$_a$), 33.44 (C$_p$), 33.75 (C$_m$), 44.35 (C$_f$), 59.30 (C$_i$), 71.99 (C$_e$), 114.50 (C$_h$), 117.76 (C$_g$), 122.89 (C$_c$),123.09(q, *J* = 274 Hz, C$_y$), 127.78 (C$_{c1}$), 130.70 (q, J = 34 Hz, C$_x$), 132.26 (C$_o$), 133.18 (C$_s$), 140.95 (C$_l$), 145.03 (C$_n$), 145.12 (C$_q$), 150.83 (C$_t$), 153.59 (C$_r$), 170.20 (C$_u$),172.33 (C$_w$), 180.61 (C$_v$), 184.68 (C$_z$). FT-IR (cm^{-1}): 682, 884, 1134, 1184, 1277, 1377, 1456, 1478, 1587, 1796, 2357, 2955. HRMS (ESI) Calculated for $C_{111}H_{115}F_{18}N_6O_{12}^+$ [M+H]$^+$: 2066.8314, found: 2066.8291.

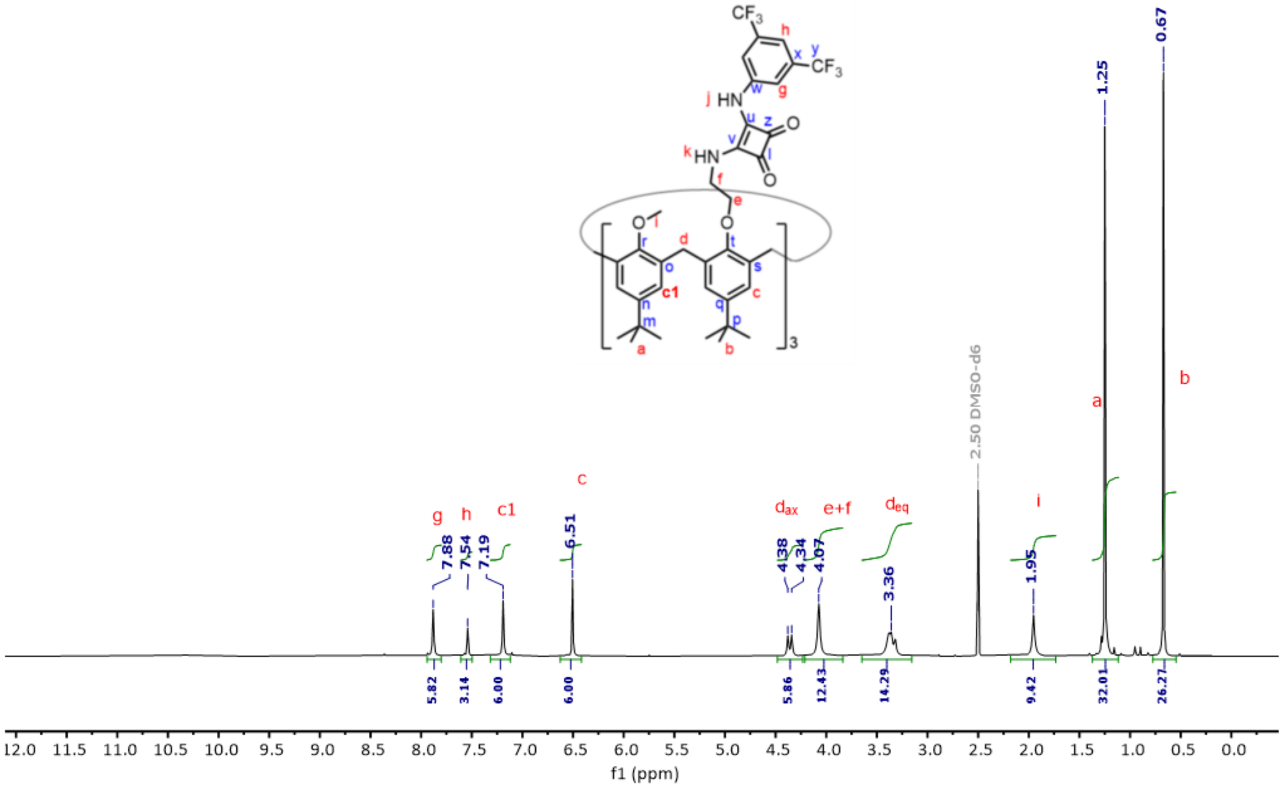

Figure S21 ^{1}H NMR spectrum (400 MHz, 298 K, DMSO-d$_6$) of calixarene **3**

[iv] values taken from the ^{1}H spectrum recorded at 1 mM in DMSO/H$_2$O 199:1, see Figure S54.

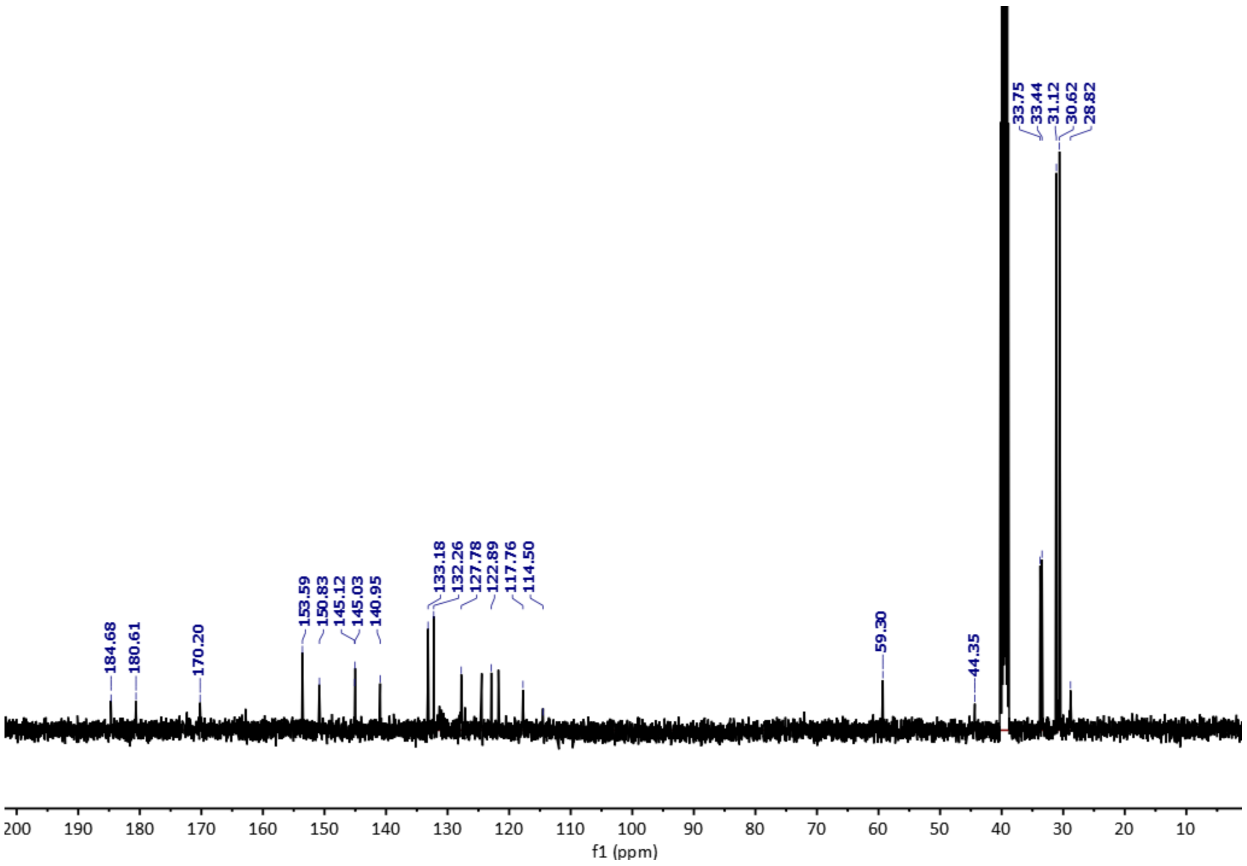

Figure S22 ^{13}C NMR spectrum (100 MHz, 298 K, $CDCl_3$) of calixarene **3**

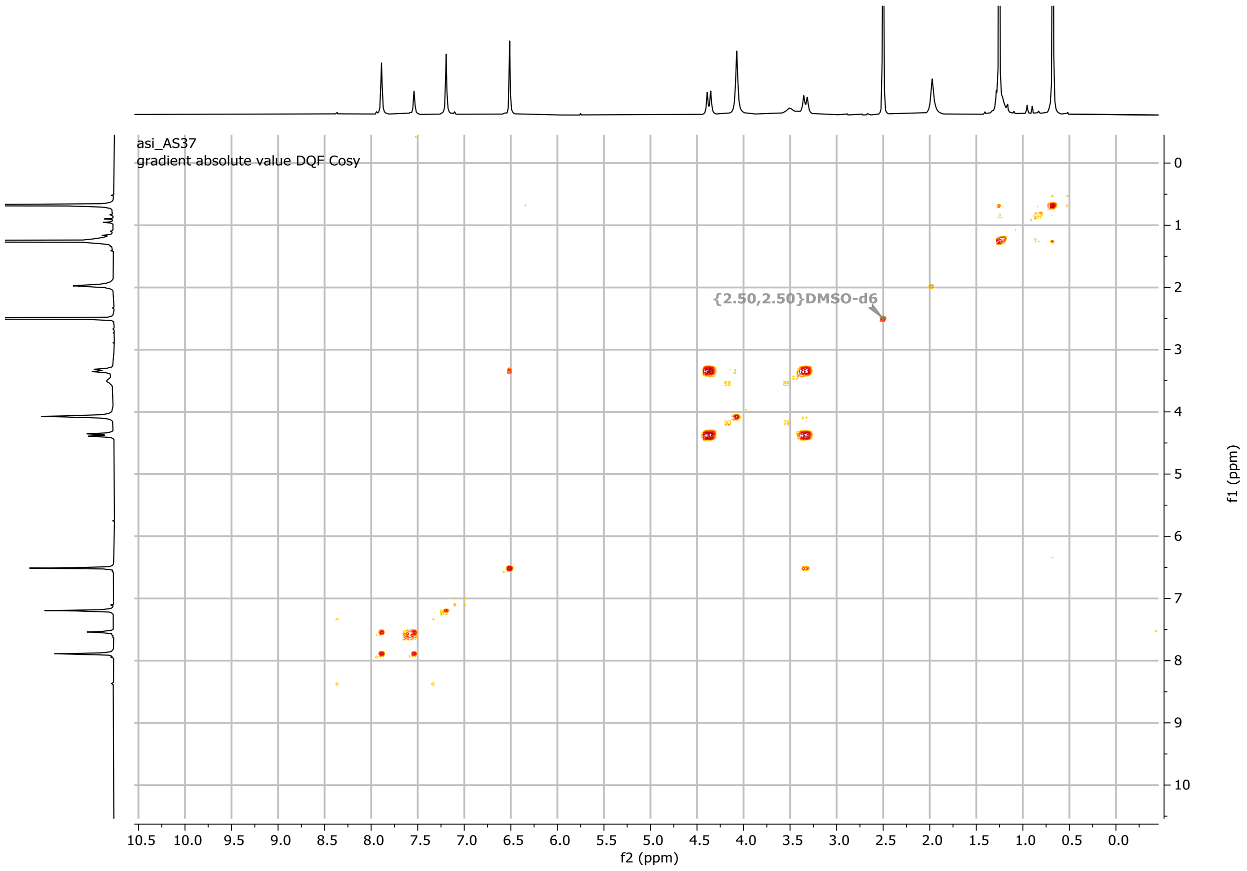

Figure S23 COSY spectrum (400 MHz, 298 K, $CDCl_3$) of calixarene **3**

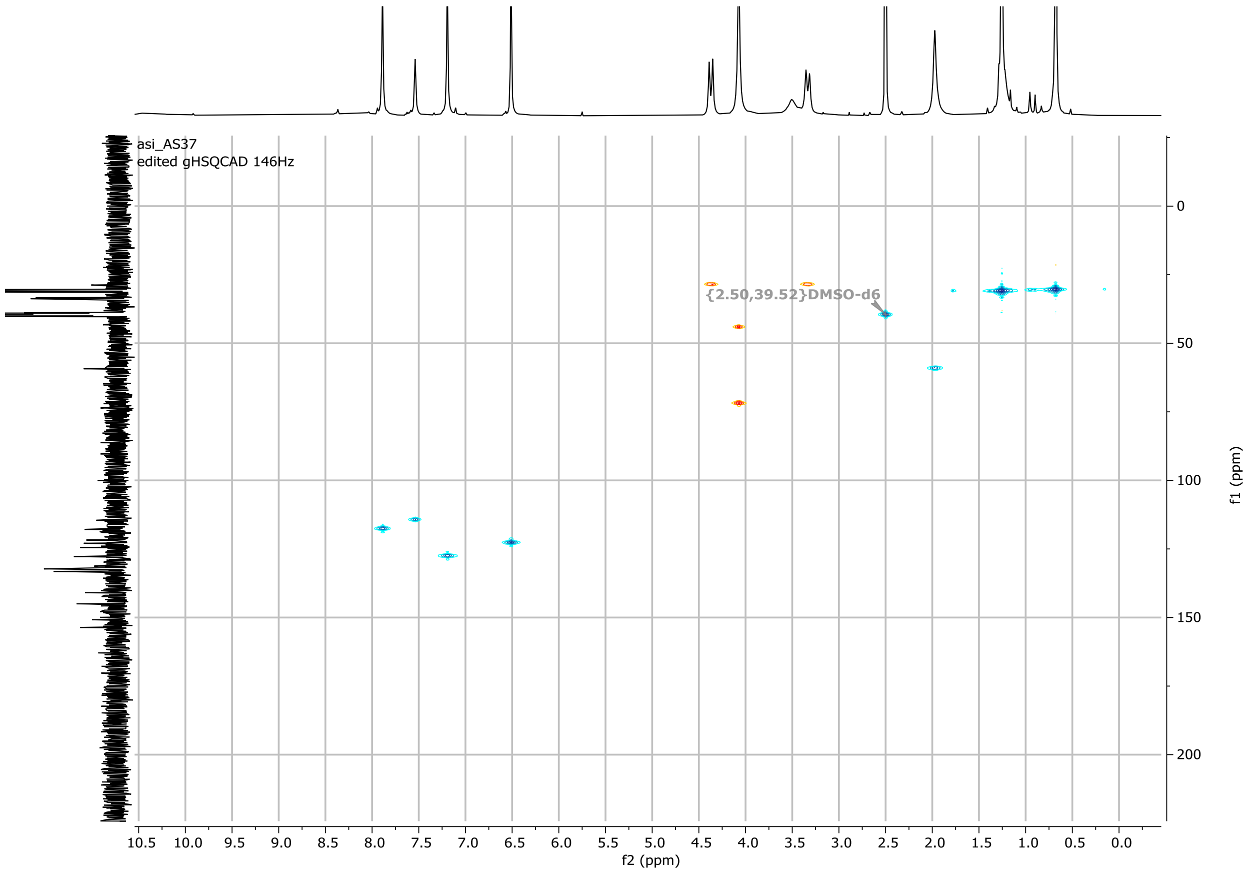

Figure S24 HSQC spectrum (400 MHz, 298 K, $CDCl_3$) of calixarene **3**

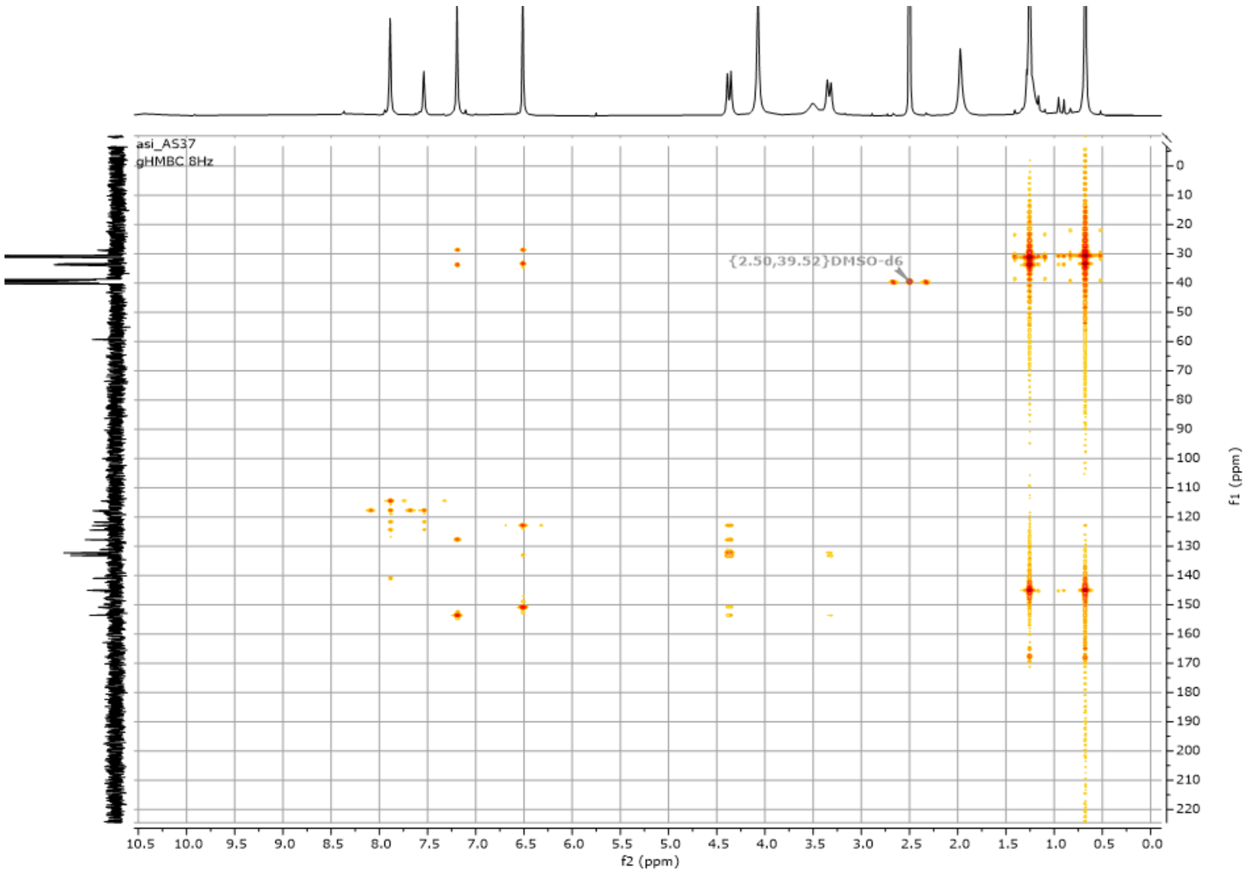

Figure S25 HMBC spectrum (400 MHz, 298 K, $CDCl_3$) of calixarene **3**

3. Anion binding studies

Binding constants were determined using ^{1}H NMR spectroscopy and by titrating tetrabutylammonium (TBA) salts into solutions of different calixarenes in deuterated solvents at 298 K. Solutions of receptors (1 mM, 1.5-2 mL) were prepared in deuterated solvent ($CDCl_3$, or acetone-d_6, or DMSO-d_6). Stock solutions of 0.5 M TBA salts were prepared by dissolving the salt (dried under vacuum) into the different receptor solutions. 500 μL of the solutions of pure receptors were transferred into NMR tubes. Initial spectra were recorded, and aliquots of the guest solutions were then added to the NMR tube and ^{1}H NMR spectra were recorded after each addition of guest.

The shifts of the signals closest to the binding site were determined and the data were fitted to a 1:1 (host:guest) binding model using the Bindfit v0.5 applet (available as freeware from Supramolecular.org).

3.1 Binding studies in acetone

^{1}H NMR titration of calixarene 1a with TBACl in acetone-d_6

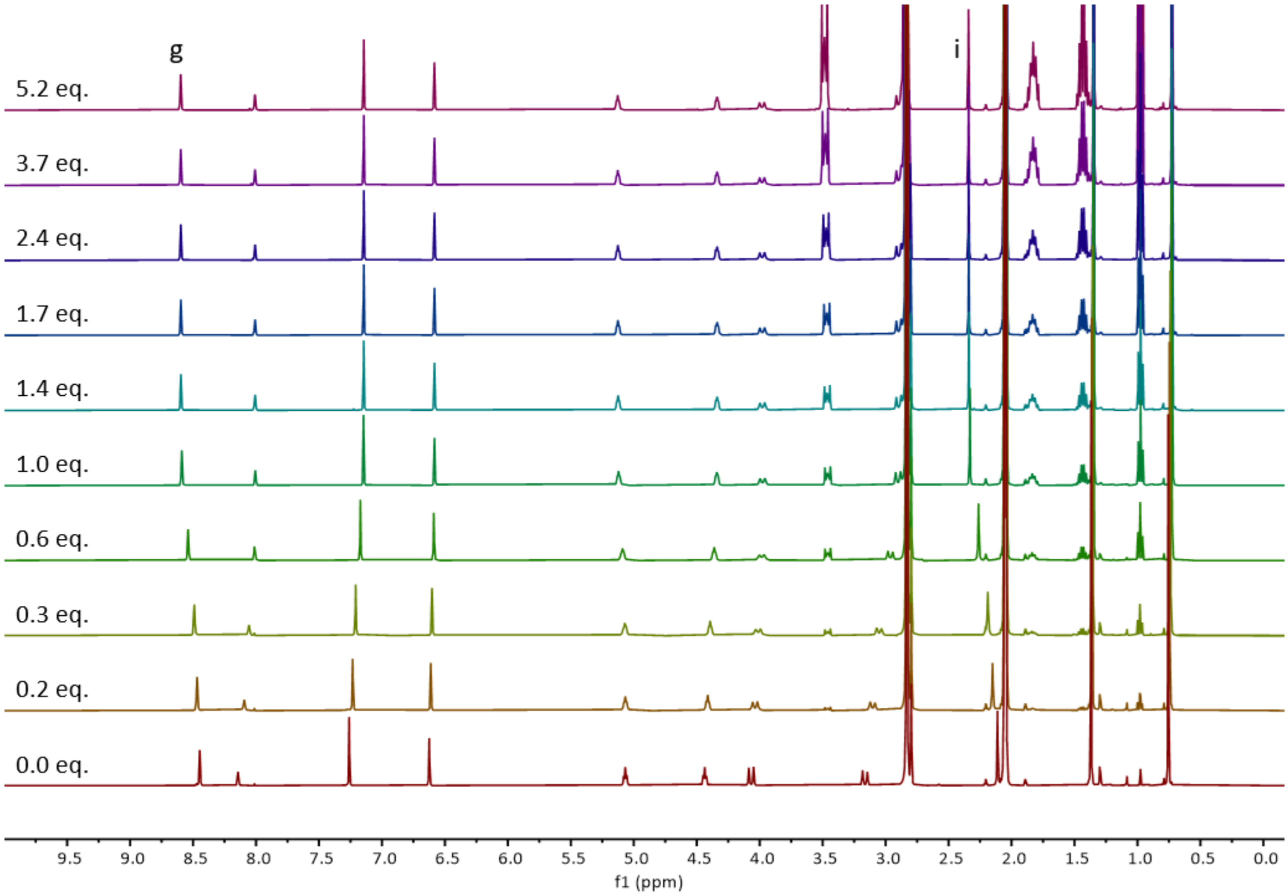

Figure S26 ^{1}H NMR spectra (400 MHz) from the titration of calixarene **1a** (1 mM) with TBACl in acetone-d_6 at 298 K. The number of equivalents of TBACl relative to **1a** is shown.

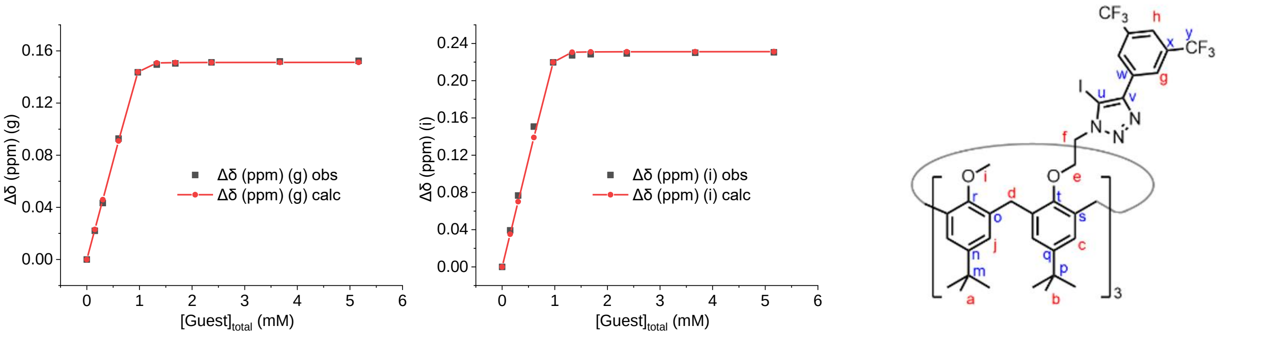

Figure S27 Observed changes in chemical shifts and calculated binding curves for the titration of **1a** (1 mM) with TBACl at 298K in acetone-d_6. The signals of protons ‘g’, ‘i’ were used for fitting to a 1:1 binding model, resulting in a K_a of 938924 M^{-1} (±208%), which is above the limit where the K_a can be accurately determined, thus $K_a > 10^5$ M^{-1}.

^{1}H NMR titration of calixarene 1c with TBACl in acetone-d_6

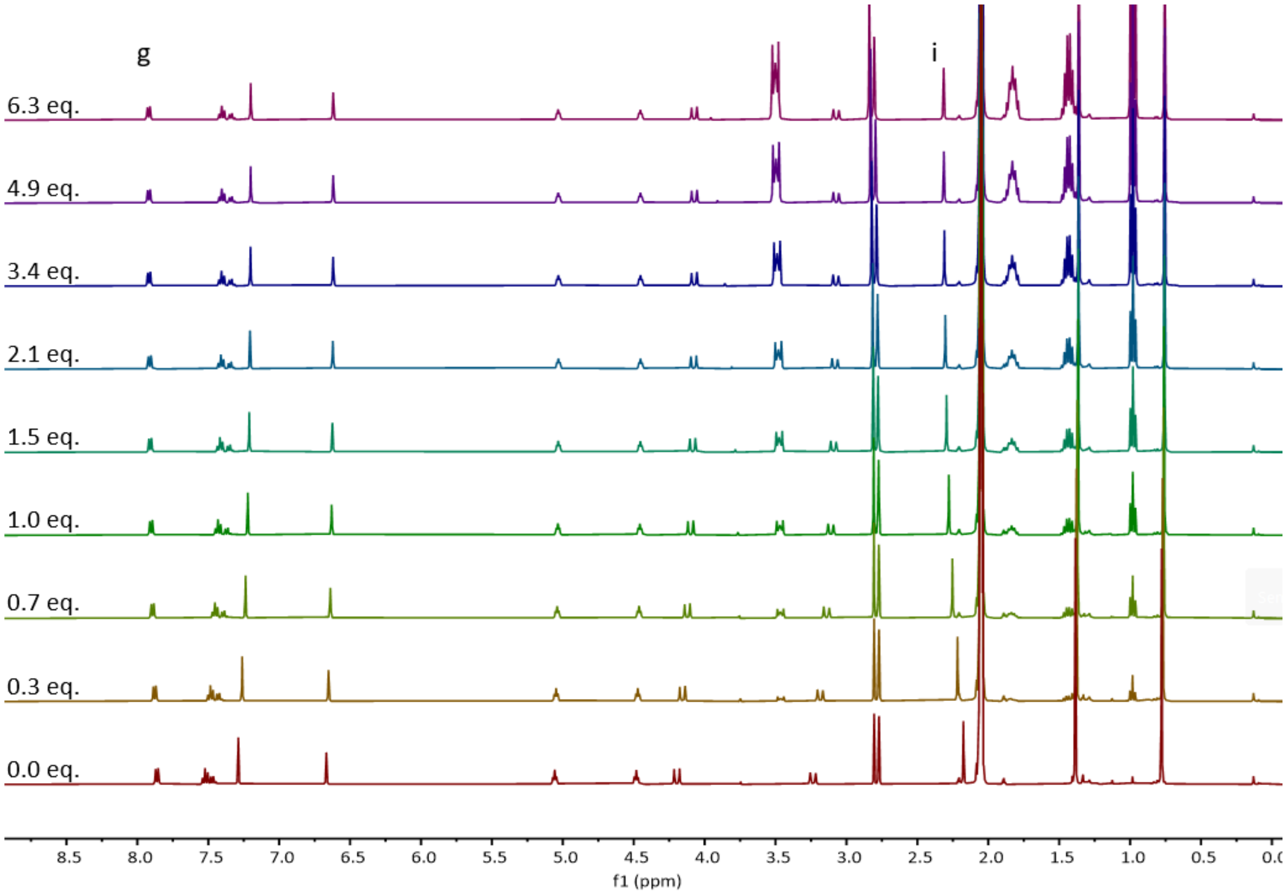

Figure S28 ^{1}H NMR spectra (400 MHz) from the titration of calixarene **1c** (1 mM) with TBACl in acetone-d_6 at 298 K. The number of equivalents of TBACl relative to **1c** is shown.

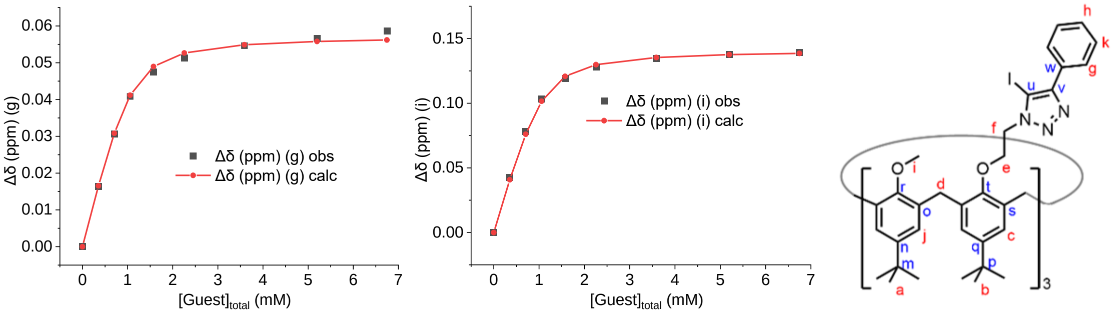

Figure S29 Observed changes in chemical shifts and calculated binding curves for the titration of **1c** (1 mM) with TBACl at 298K in acetone-d_6. The signals of protons 'g', 'i' were used for fitting to a 1:1 binding model, resulting in a K_a of 8936 M^{-1} (±10%).

^{1}H NMR titration of calixarene 2 with TBACl in acetone-d$_6$

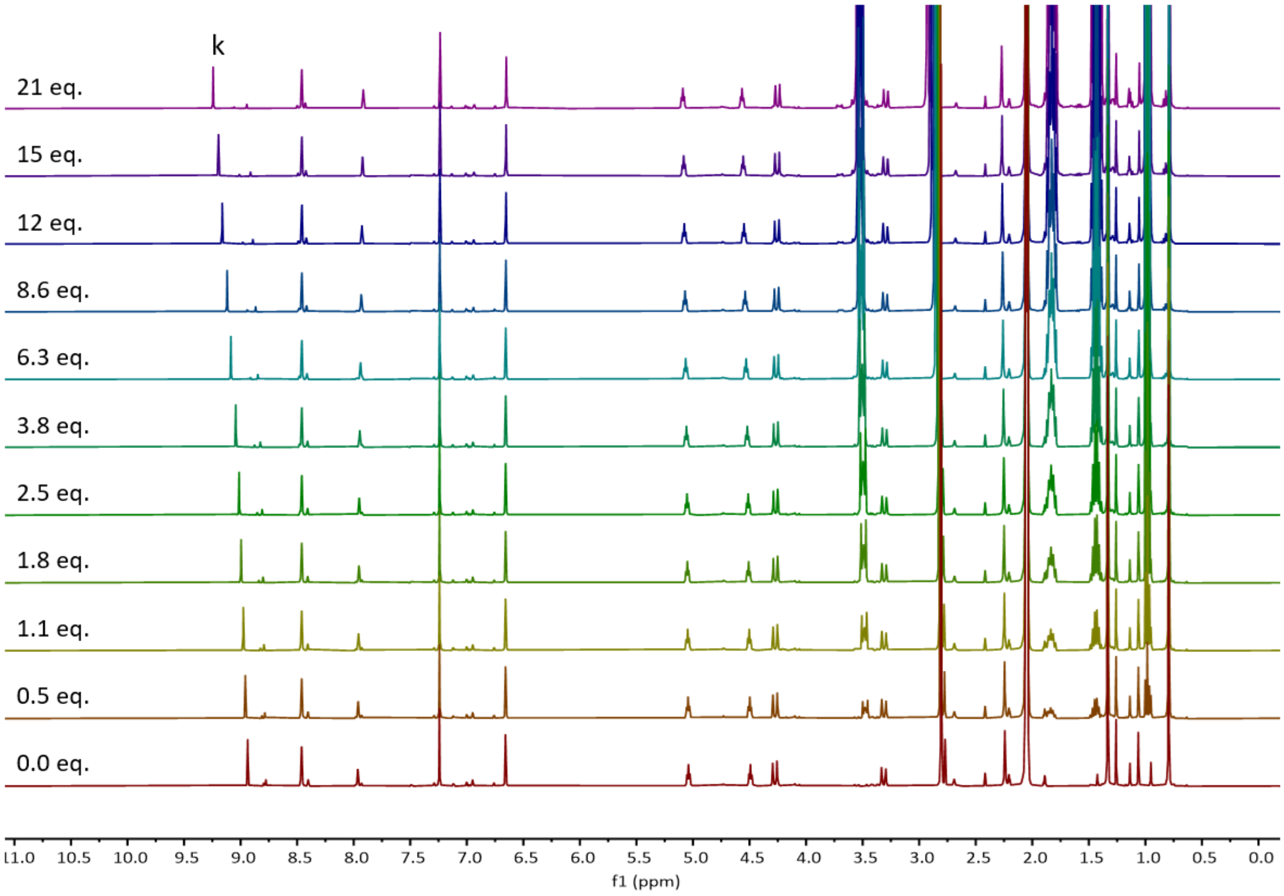

Figure S30 ^{1}H NMR spectra (400 MHz) from the titration of calixarene **2** (1 mM) with TBACl in acetone-d$_6$ at 298 K. The number of equivalents of TBACl relative to **2** is shown.

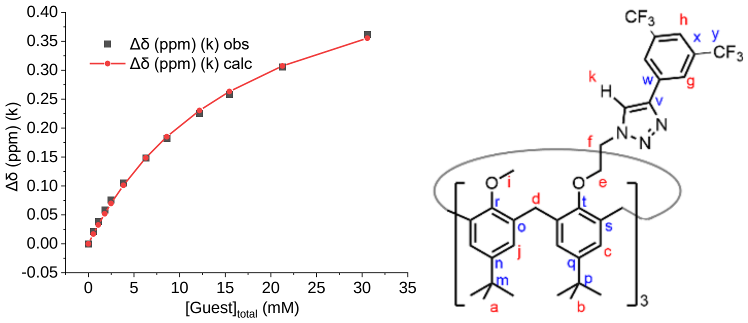

Figure S31 Observed changes in chemical shifts and calculated binding curves for the titration of **2** (1 mM) with TBACl at 298K in acetone-d$_6$. The signal of proton ‘k’ was used for fitting to a 1:1 binding model, resulting in a K_a of 58 M^{-1} (±3.69%).

^{1}H NMR titration of calixarene 3 with TBACl in acetone-d_6

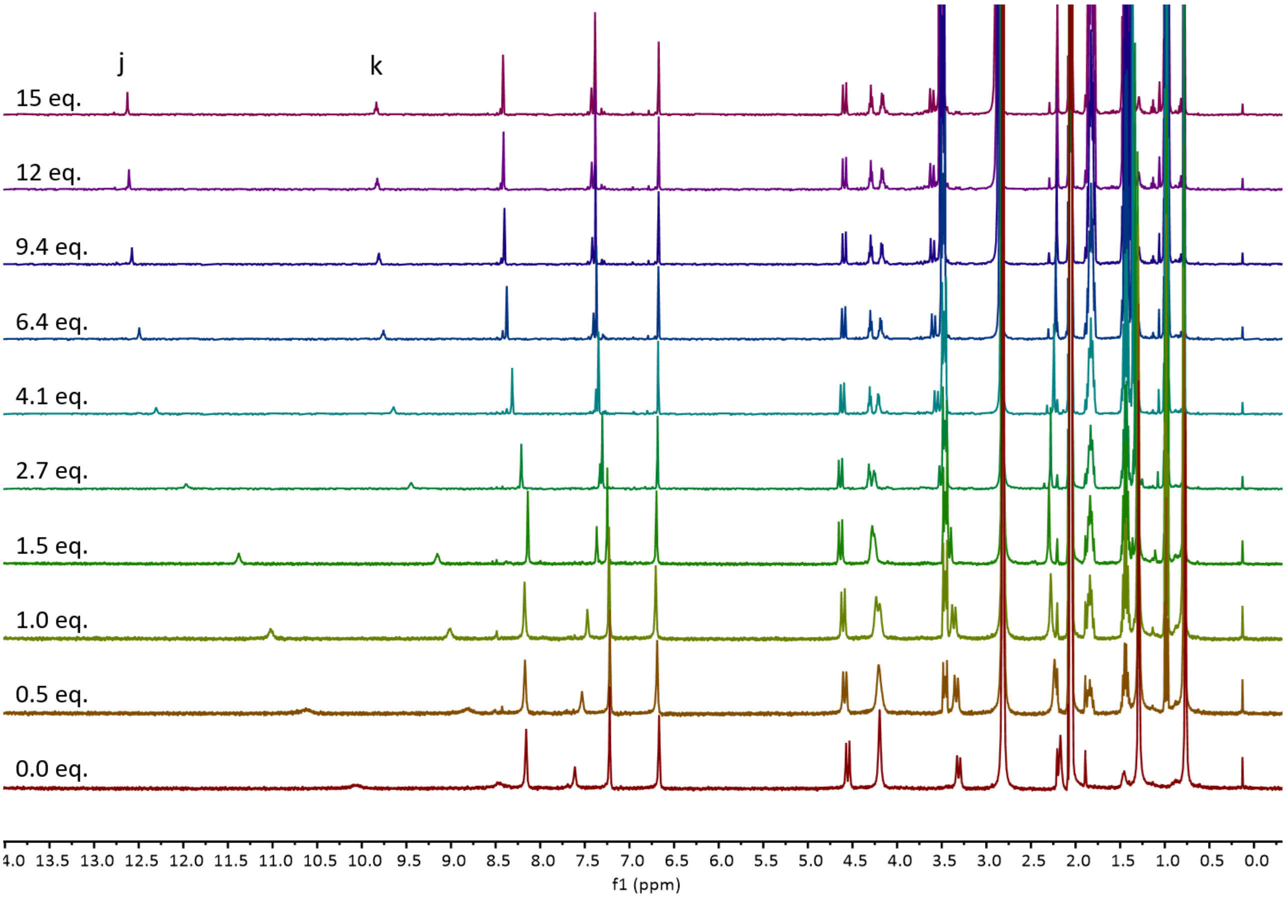

Figure S32 ^{1}H NMR spectra (400 MHz) from the titration of calixarene **3** (1 mM) with TBACl in acetone-d_6 at 298 K. The number of equivalents of TBACl relative to **3** is shown.

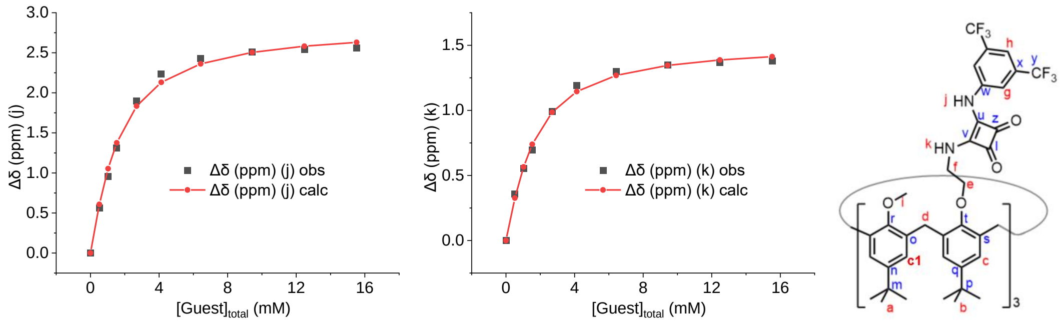

Figure S33 Observed changes in chemical shifts and calculated binding curves for the titration of **3** (1 mM) with TBACl at 298K in acetone-d_6. The signals of protons 'j', 'k' were used for fitting to a 1:1 binding model, resulting in a K_a of 871 M^{-1} (±8.32 %).

^{1}H NMR titration of calixarene 4 with TBACl in acetone-d_6

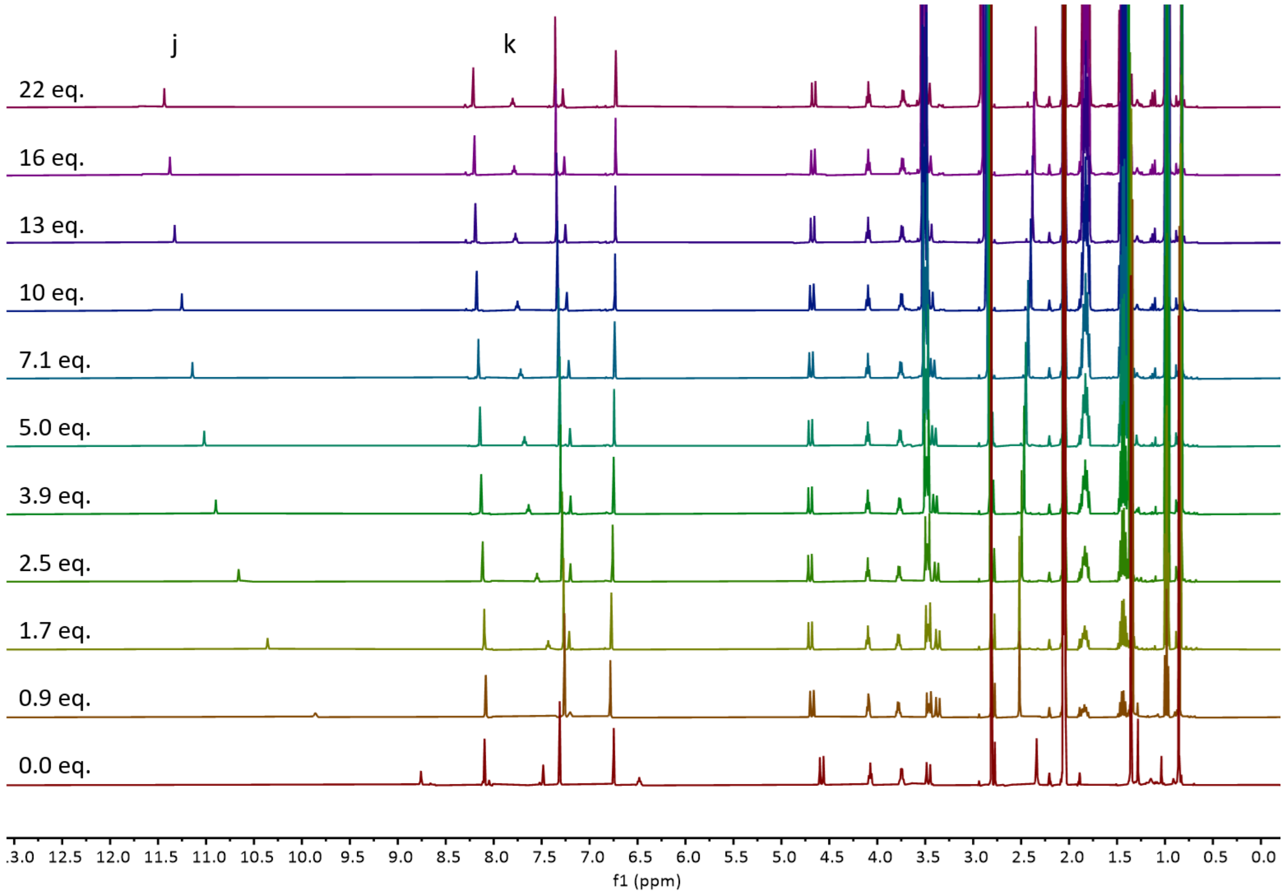

Figure S34 ^{1}H NMR spectra (400 MHz) from the titration of calixarene **4** (1 mM) with TBACl in acetone-d_6 at 298 K. The number of equivalents of TBACl relative to **4** is shown.

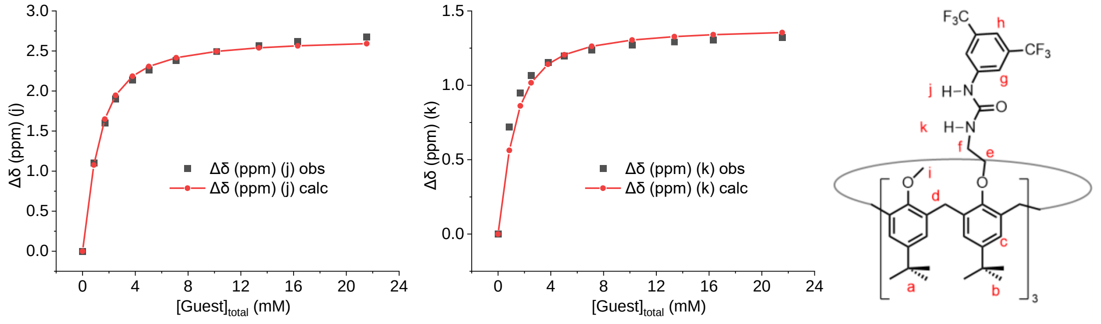

Figure S35 Observed changes in chemical shifts and calculated binding curves for the titration of **4** (1 mM) with TBACl at 298K in acetone-d_6. The signals of protons 'j', 'k' were used for fitting to a 1:1 binding model, resulting in a K_a of 1378 M^{-1} (±10.7 %).

^{1}H NMR titration of calixarene 5 with TBACl in acetone-d_6

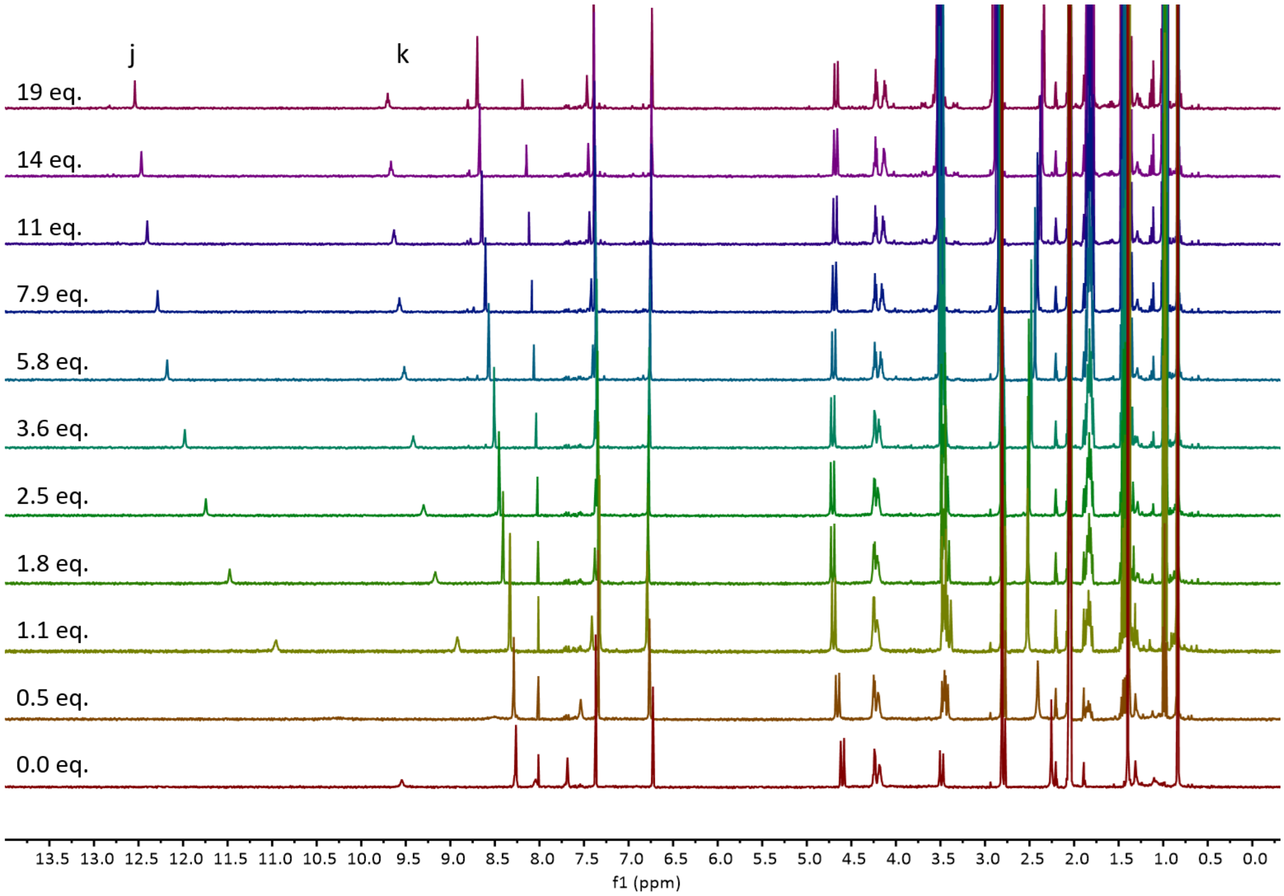

Figure S36 ^{1}H NMR spectra (400 MHz) from the titration of calixarene **5** (1 mM) with TBACl in acetone-d_6 at 298 K. The number of equivalents of TBACl relative to **5** is shown.

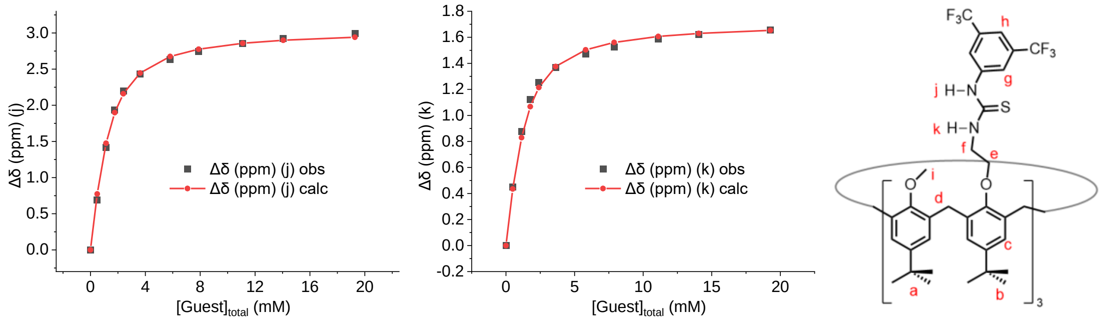

Figure S37 Observed changes in chemical shifts and calculated binding curves for the titration of **5** (1 mM) with TBACl at 298K in acetone-d_6. The signals of protons 'j', 'k' were used for fitting to a 1:1 binding model, resulting in a K_a of 1358 M^{-1} (±5.98%).

3.2 Binding studies in chloroform

^{1}H NMR titration of calixarene 1a with TBACl in $CDCl_3$

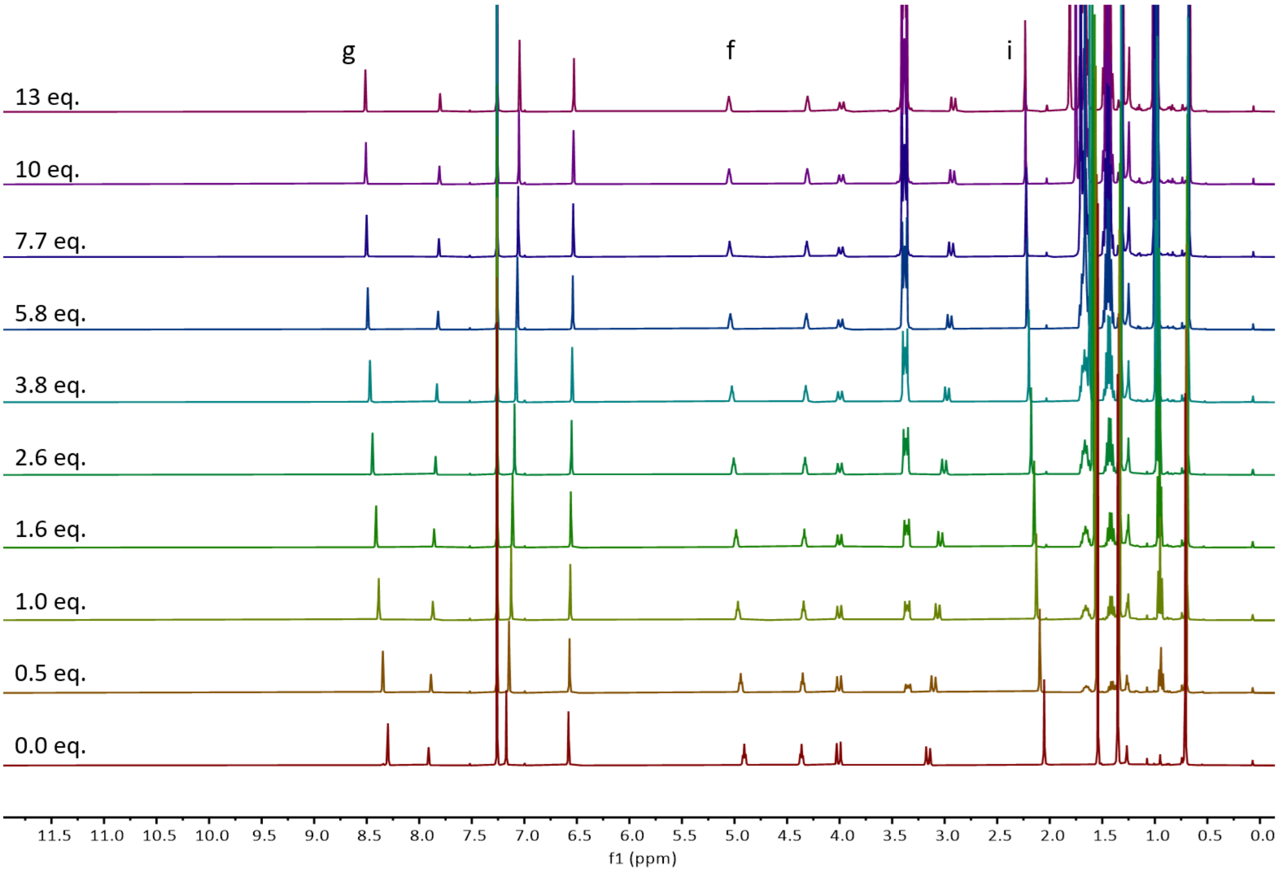

Figure S38 ^{1}H NMR spectra (400 MHz, 298 K) from the titration of calixarene **1a** (1 mM) with TBACl in $CDCl_3$ at 298 K. The number of equivalents of TBACl relative to **1a** is shown.

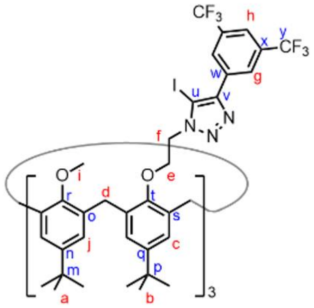

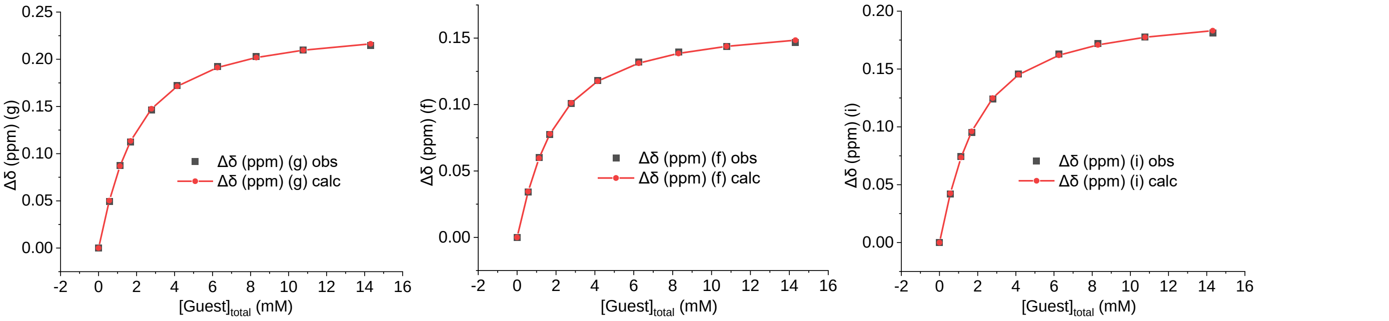

Figure S39 Observed changes in chemical shifts and calculated binding curves for the titration of **1a** (1 mM) with TBACl at 298 K in $CDCl_3$. The signals of protons ‘g’, ‘f’, ‘i’ were used for fitting to a 1:1 binding model, resulting in a K_a of 771 M^{-1} (±1.21%).

^{1}H NMR titration of calixarene 1a with TBABr in $CDCl_3$

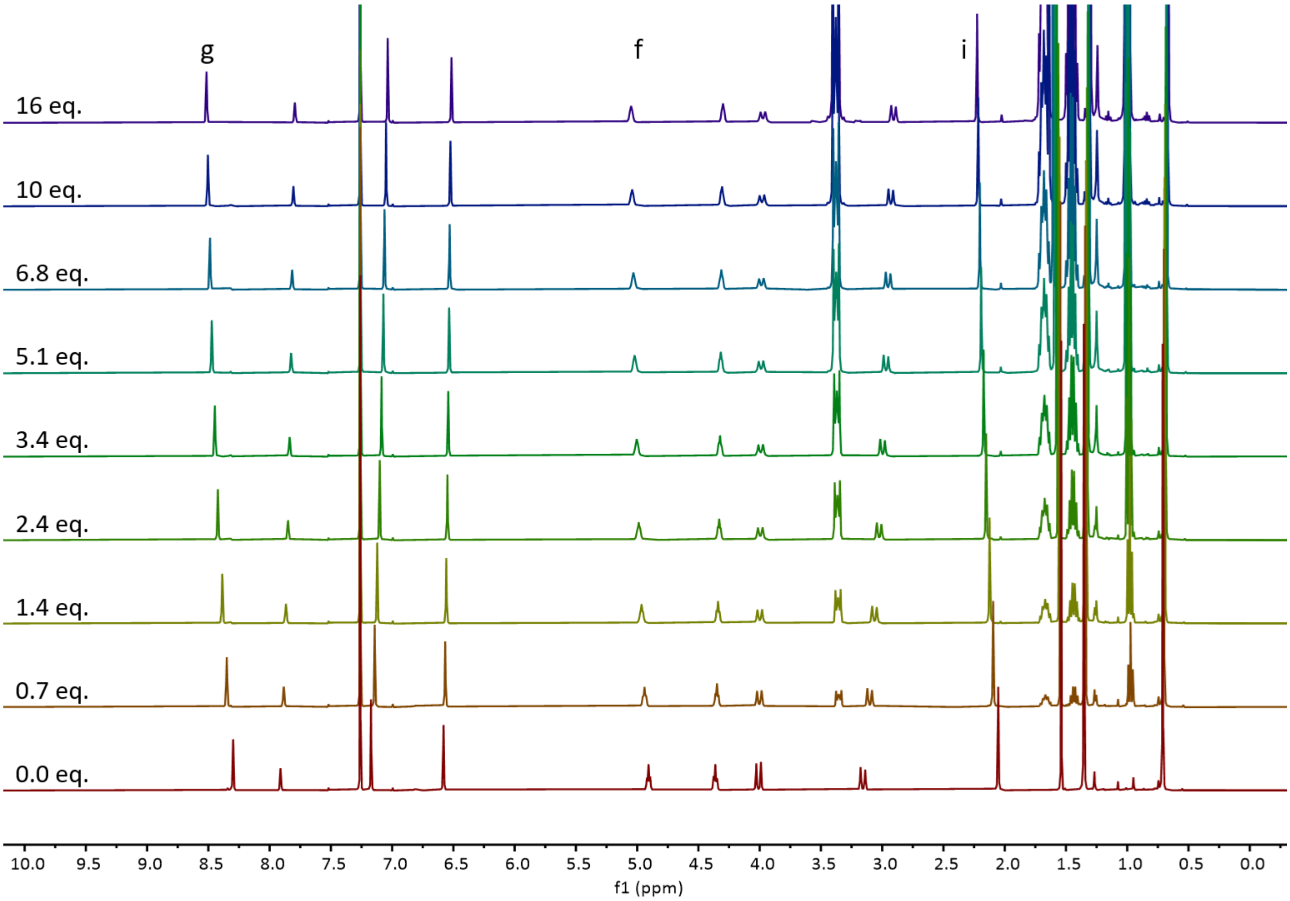

Figure S40 ^{1}H NMR spectra (400 MHz) from the titration of calixarene **1a** (1 mM) with TBABr in $CDCl_3$ at 298 K. The number of equivalents of TBABr relative to **1a** is shown.

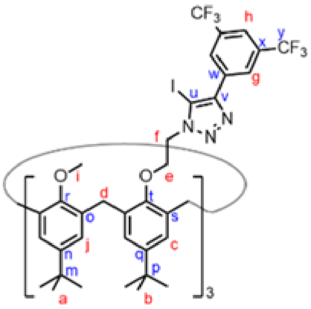

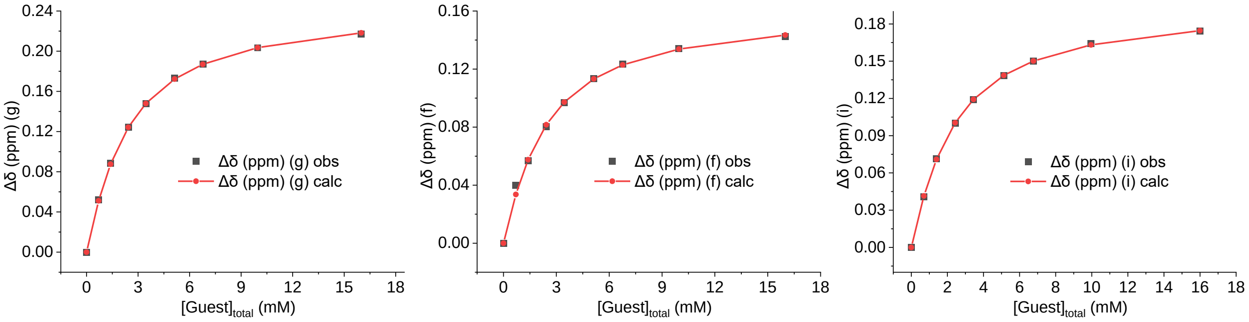

Figure S41 Observed changes in chemical shifts and calculated binding curves for the titration of **1a** (1 mM) with TBABr at 298K in $CDCl_3$. The signals of protons ‘g’, ‘f’, ‘i’ were used for fitting to a 1:1 binding model, resulting in a K_a of 517 M^{-1} (±1.78%).

^{1}H NMR titration of calixarene 1a with $TBAH_2PO_4$ in $CDCl_3$

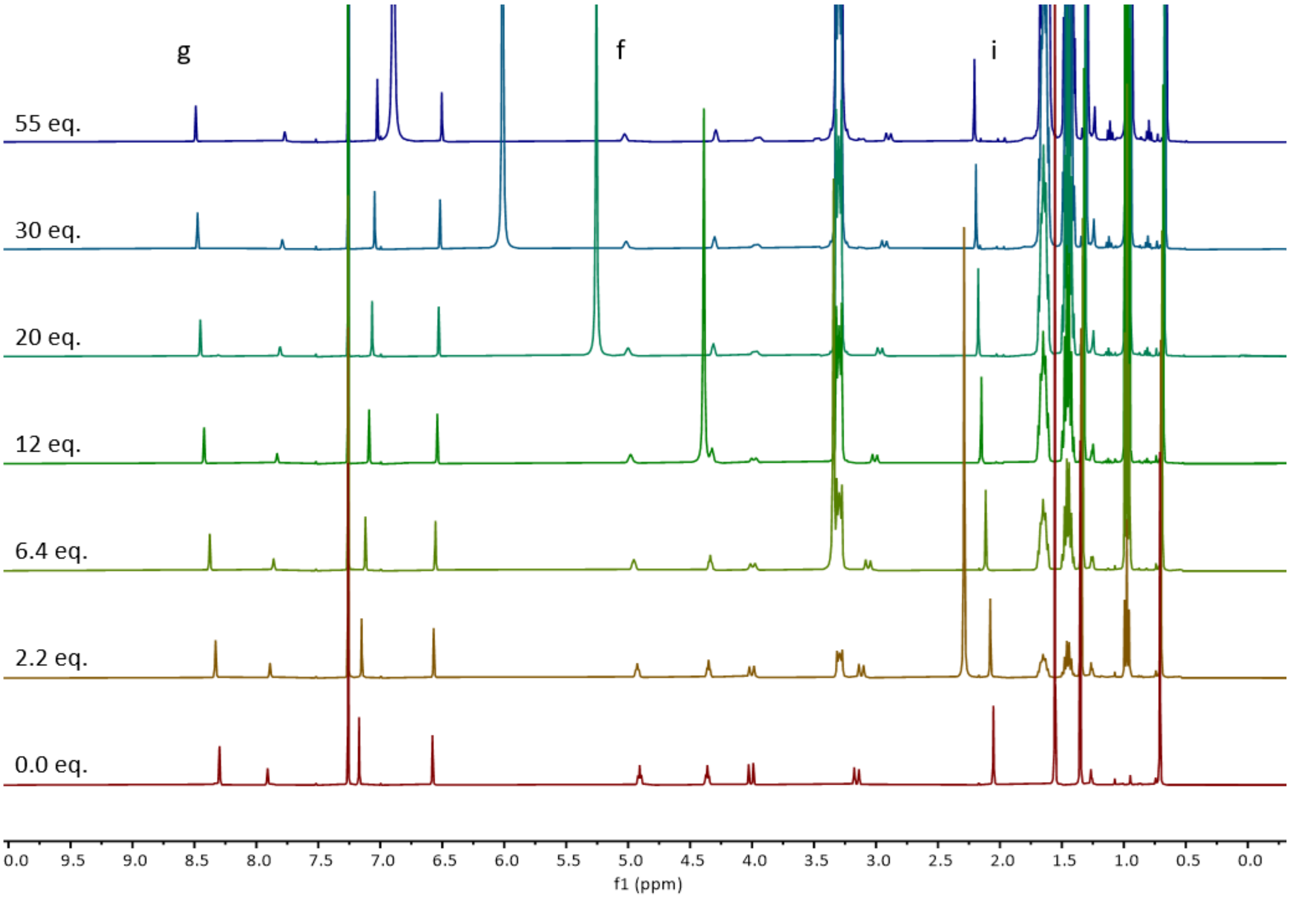

Figure S42 ^{1}H NMR spectra (400 MHz) from the titration of calixarene **1a** (1 mM) with $TBAH_2PO_4$ in $CDCl_3$ at 298 K. The number of equivalents of $TBAH_2PO_4$ relative to **1a** is shown.

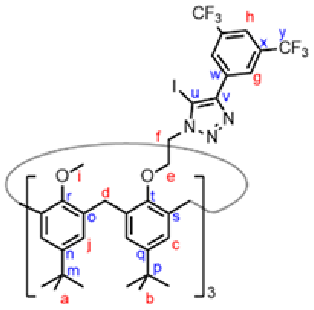

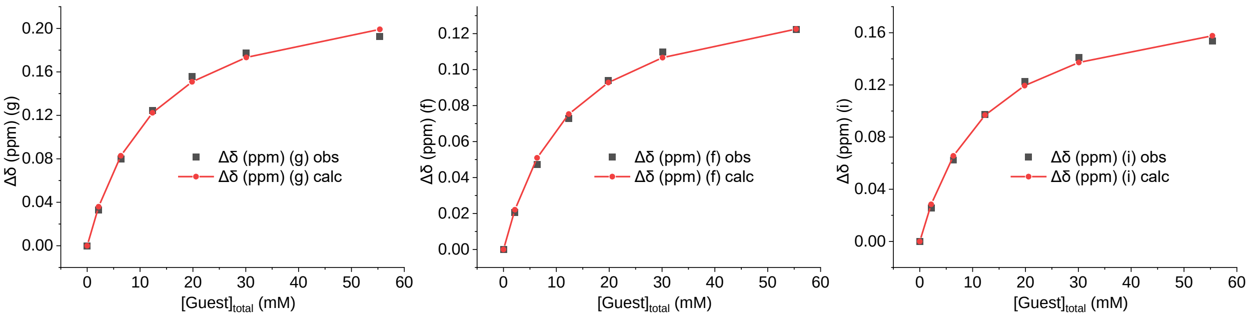

Figure S43 Observed changes in chemical shifts and calculated binding curves for the titration of **1a** (1 mM) with $TBAH_2PO_4$ at 298K in $CDCl_3$. The signals of protons ‘g’, ‘f’, ‘i’ were used for fitting to a 1:1 binding model, resulting in a K_a of 80 M^{-1} (±4.38%).

^{1}H NMR titration of calixarene 1a with $TBANO_3$ in $CDCl_3$

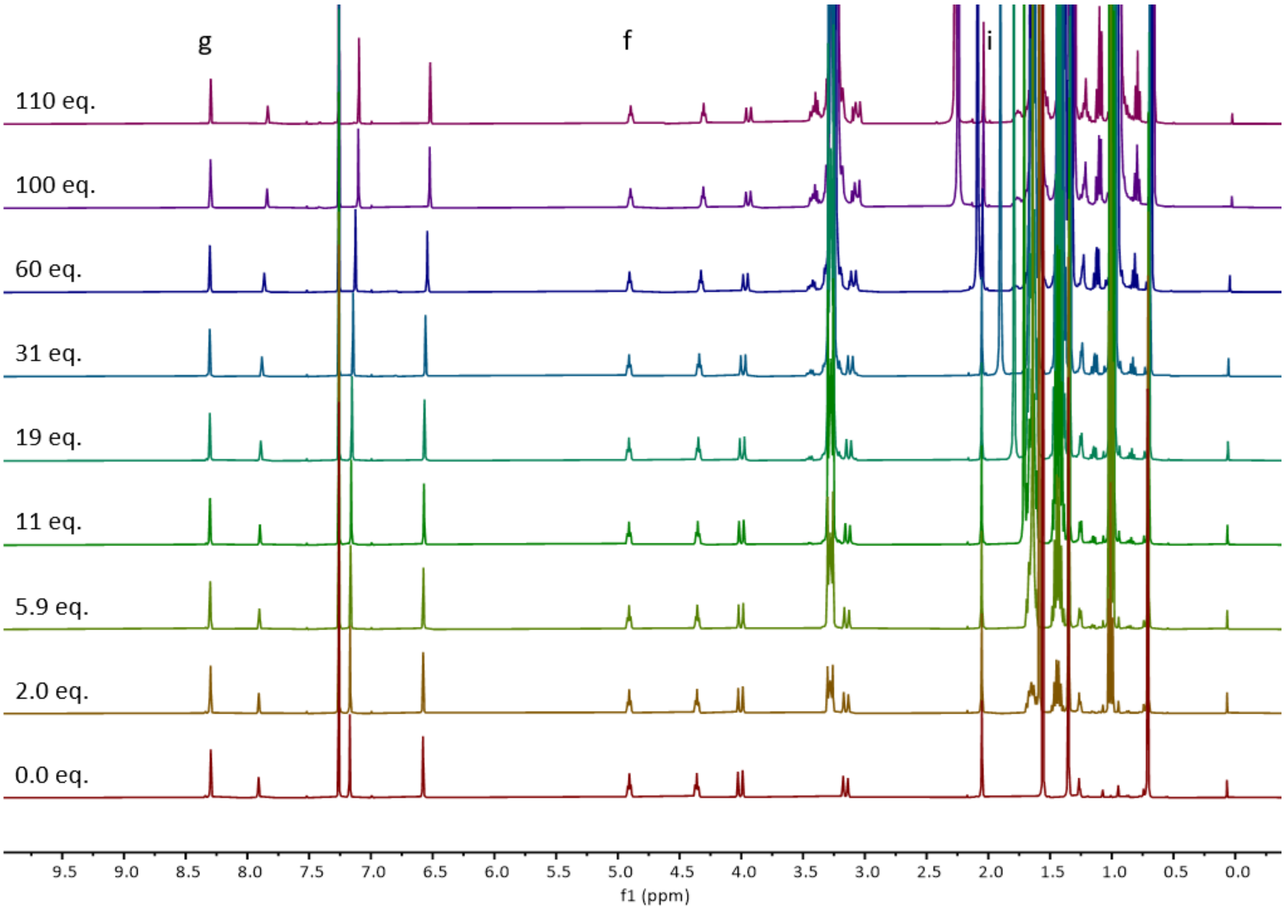

Figure S44 ^{1}H NMR spectra (400 MHz) from the titration of calixarene **1a** (1 mM) with $TBANO_3$ in $CDCl_3$ at 298 K. The number of equivalents of $TBANO_3$ relative to **1a** is shown.

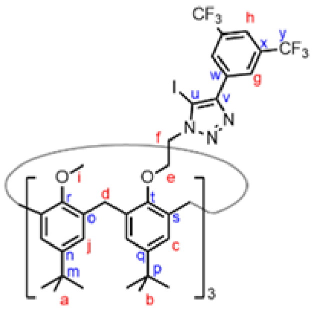

For this titration, no significant change in the chemical shift of the proton signals was observed, and we thus conclude that the K_a (1:1) < 10 M^{-1}.

^{1}H NMR titration of calixarene 1a with TBAOAc in $CDCl_3$

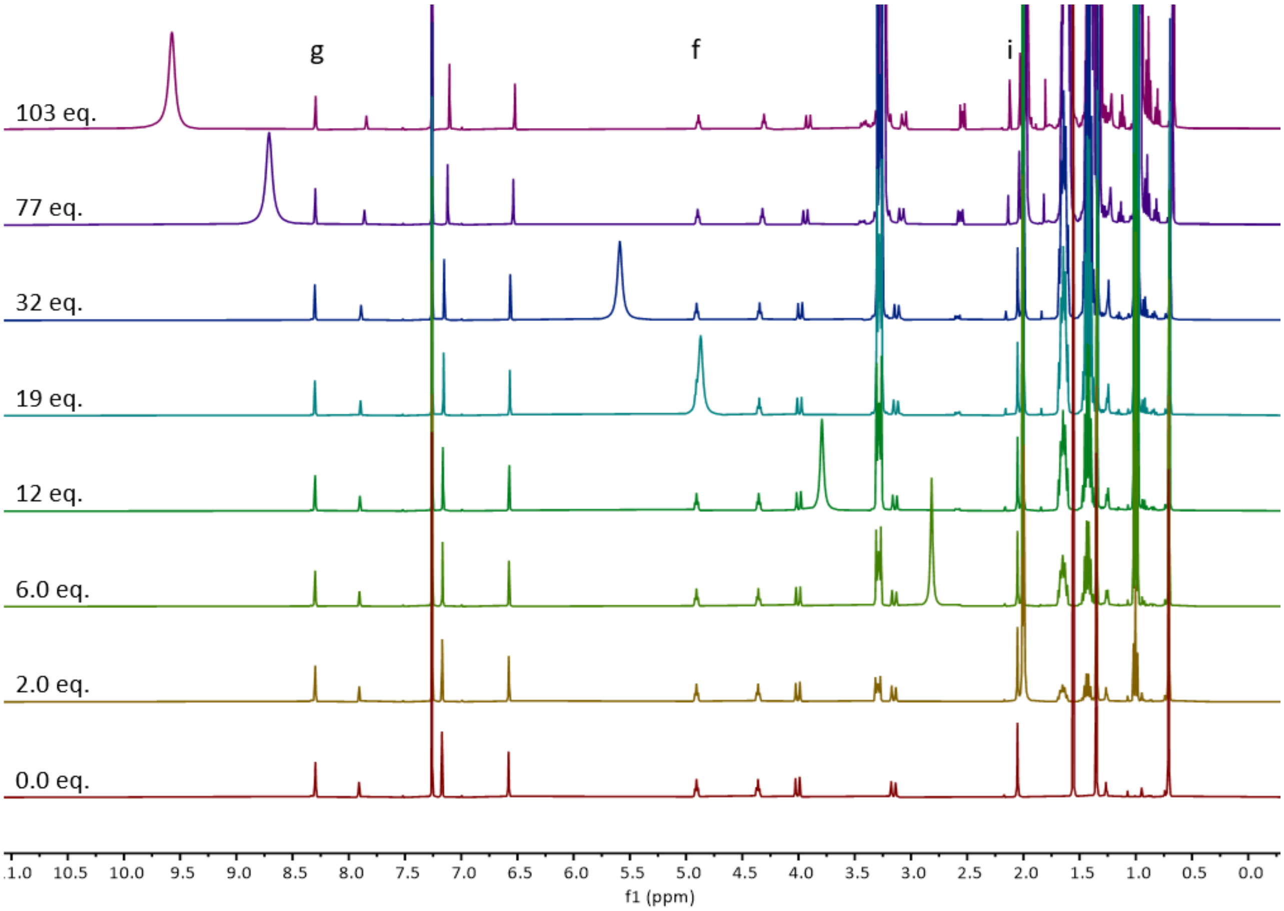

Figure S45 ^{1}H NMR spectra (400 MHz) from the titration of calixarene **1a** (1 mM) with TBAOAc in $CDCl_3$ at 298 K. The number of equivalents of TBAOAc relative to **1a** is shown.

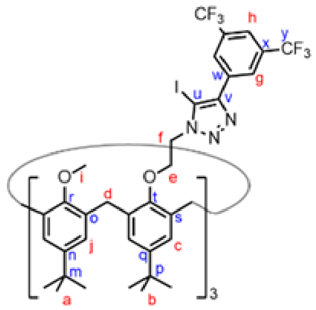

For this titration, no significant change in the chemical shift of the proton signals was observed, and we thus conclude that the K_a (1:1) < 10 M^{-1}.

Table S1 Binding constants of calixarene **1a** in $CDCl_3$ with various anions

**Compound**	**K_a (M^{-1})**				
	**Cl^-**	**Br^-**	**$H_2PO_4^-$**	**NO_3^-**	**OAc^-**
**1a**	770	520	80	<10	<10

^{1}H NMR titration of calixarene 1b with TBACl in $CDCl_3$

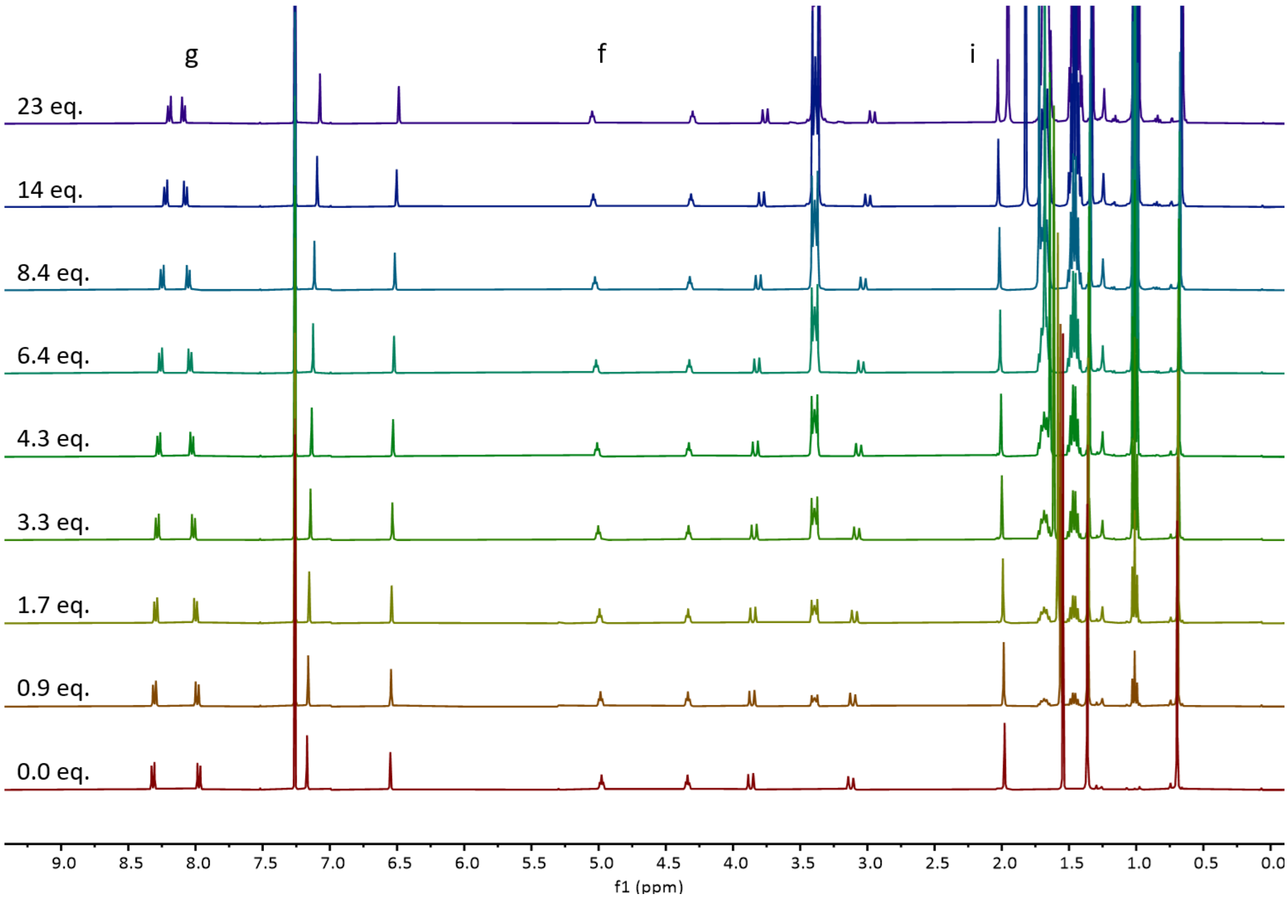

Figure S46 ^{1}H NMR spectra (400 MHz) from the titration of calixarene **1b** (1 mM) with TBACl in $CDCl_3$ at 298 K. The number of equivalents of TBACl relative to **1b** is shown.

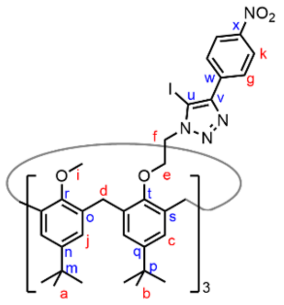

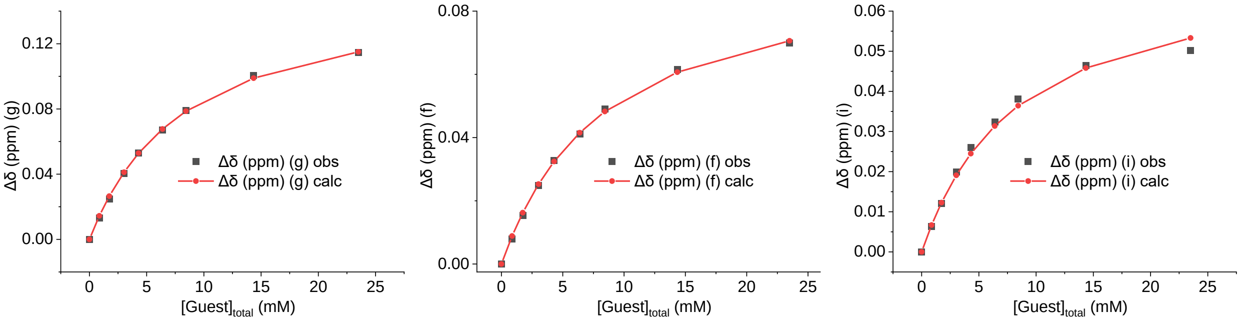

Figure S47 Observed changes in chemical shifts and calculated binding curves for the titration of **1b** (1 mM) with TBACl at 298K in $CDCl_3$. The signals of protons ‘g’, ‘f’, ‘i’ were used for fitting to a 1:1 binding model, resulting in a K_a of 128 M^{-1} (±2.27%).

^{1}H NMR titration of calixarene 1c with TBACl in $CDCl_3$

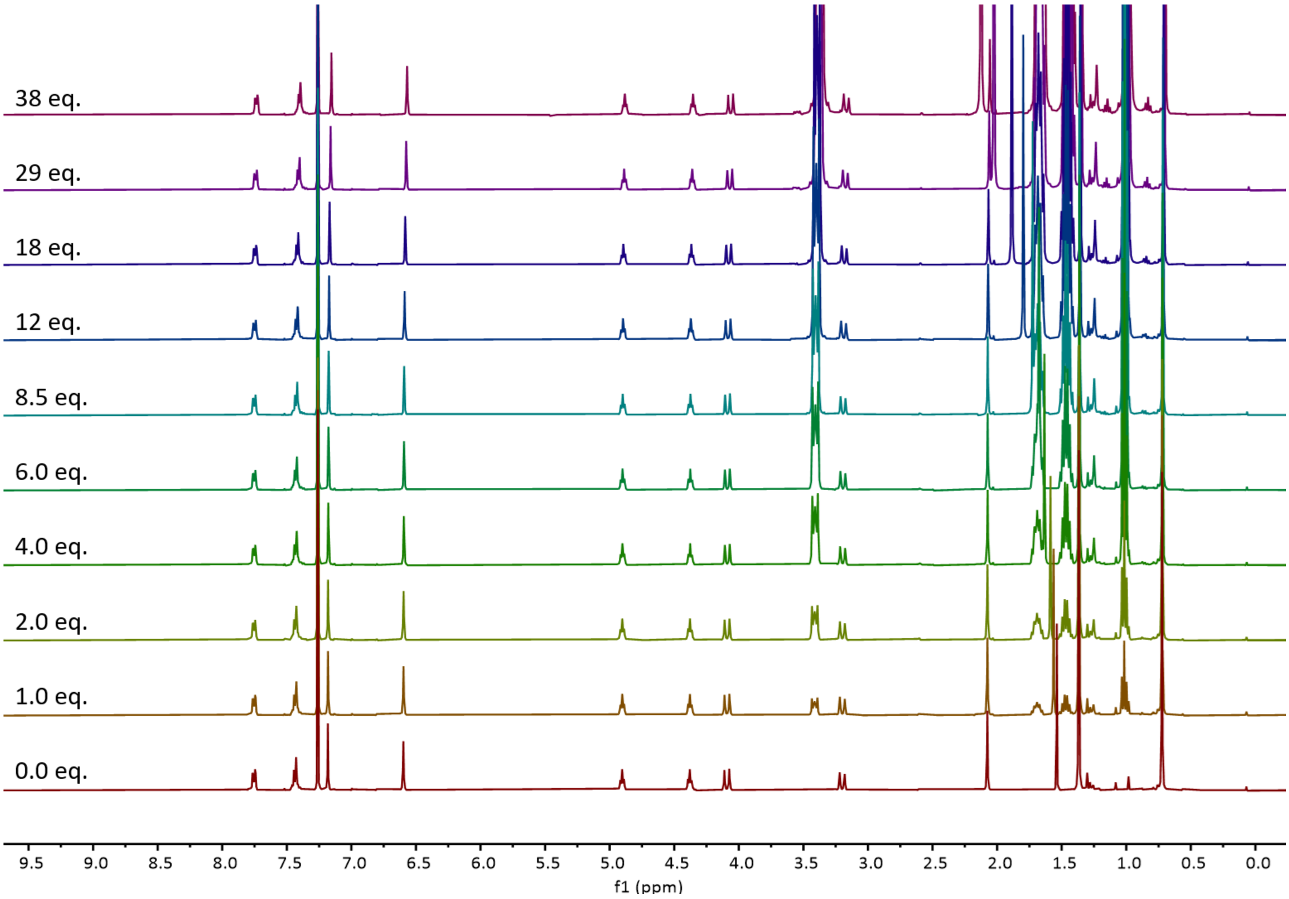

Figure S48 ^{1}H NMR spectra (400 MHz) from the titration of calixarene **1c** (1 mM) with TBACl in $CDCl_3$ at 298 K. The number of equivalents of TBACl relative to **1c** is shown.

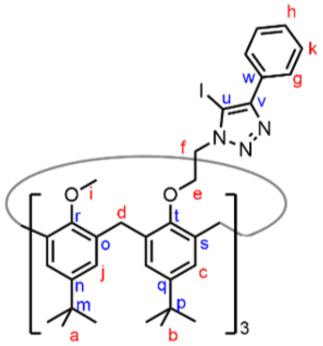

For this titration, no significant change in the chemical shift of the proton signals was observed, and we thus conclude that the K_a (1:1) < 10 M^{-1}.

^{1}H NMR titration of calixarene 2 with TBACl in $CDCl_3$

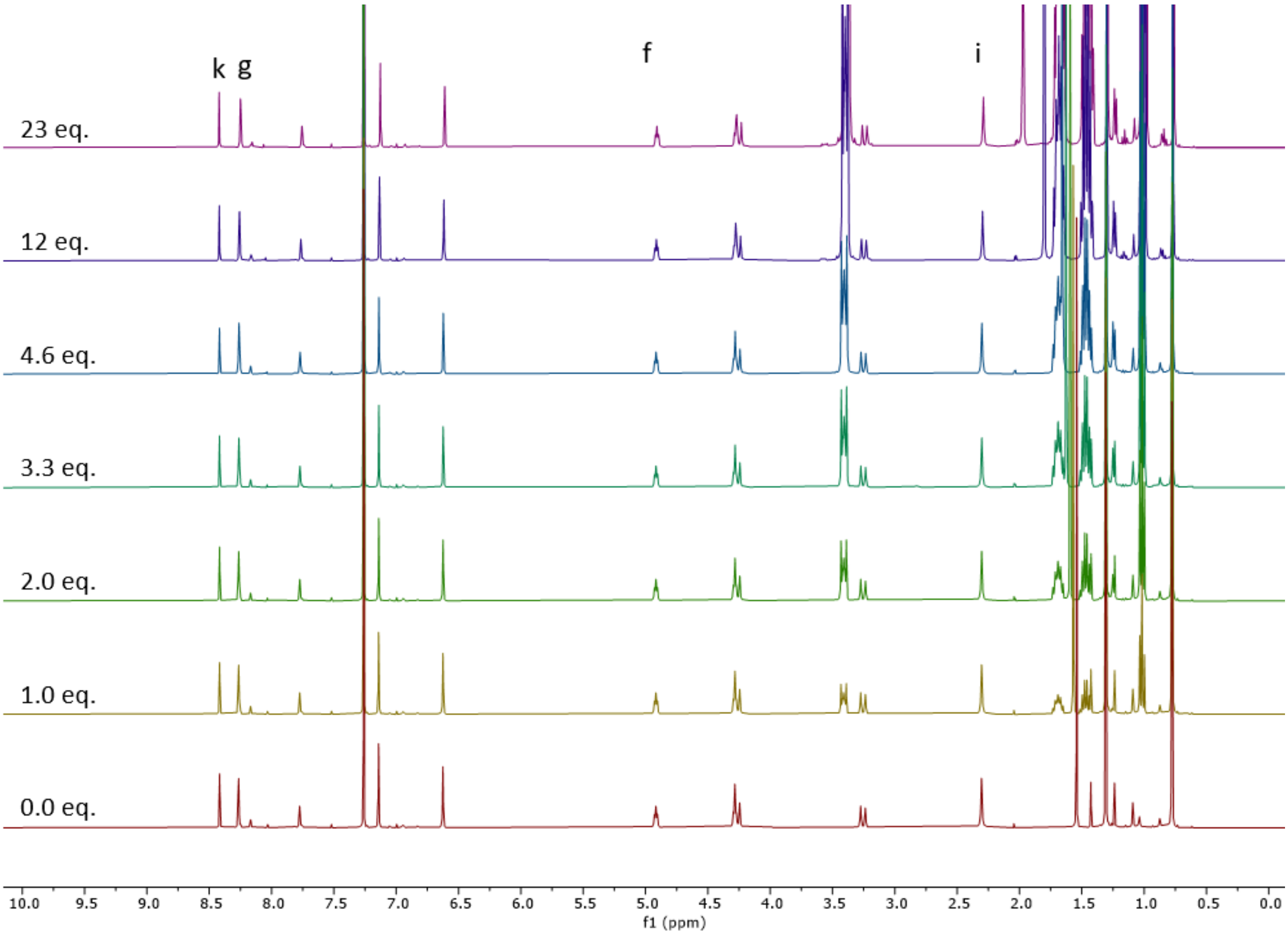

Figure S49 ^{1}H NMR spectra (400 MHz) from the titration of calixarene **2** (1 mM) with TBACl in $CDCl_3$ at 298 K. The number of equivalents of TBACl relative to **2** is shown.

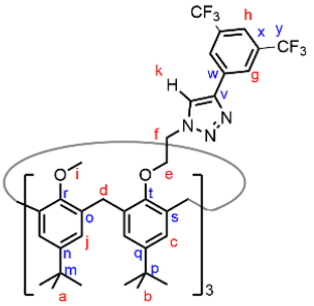

For this titration, no significant change in the chemical shift of the proton signals was observed, and we thus conclude that the K_a (1:1) < 10 M^{-1}.

^{1}H NMR titration of calixarene 4 with TBACl in $CDCl_3$

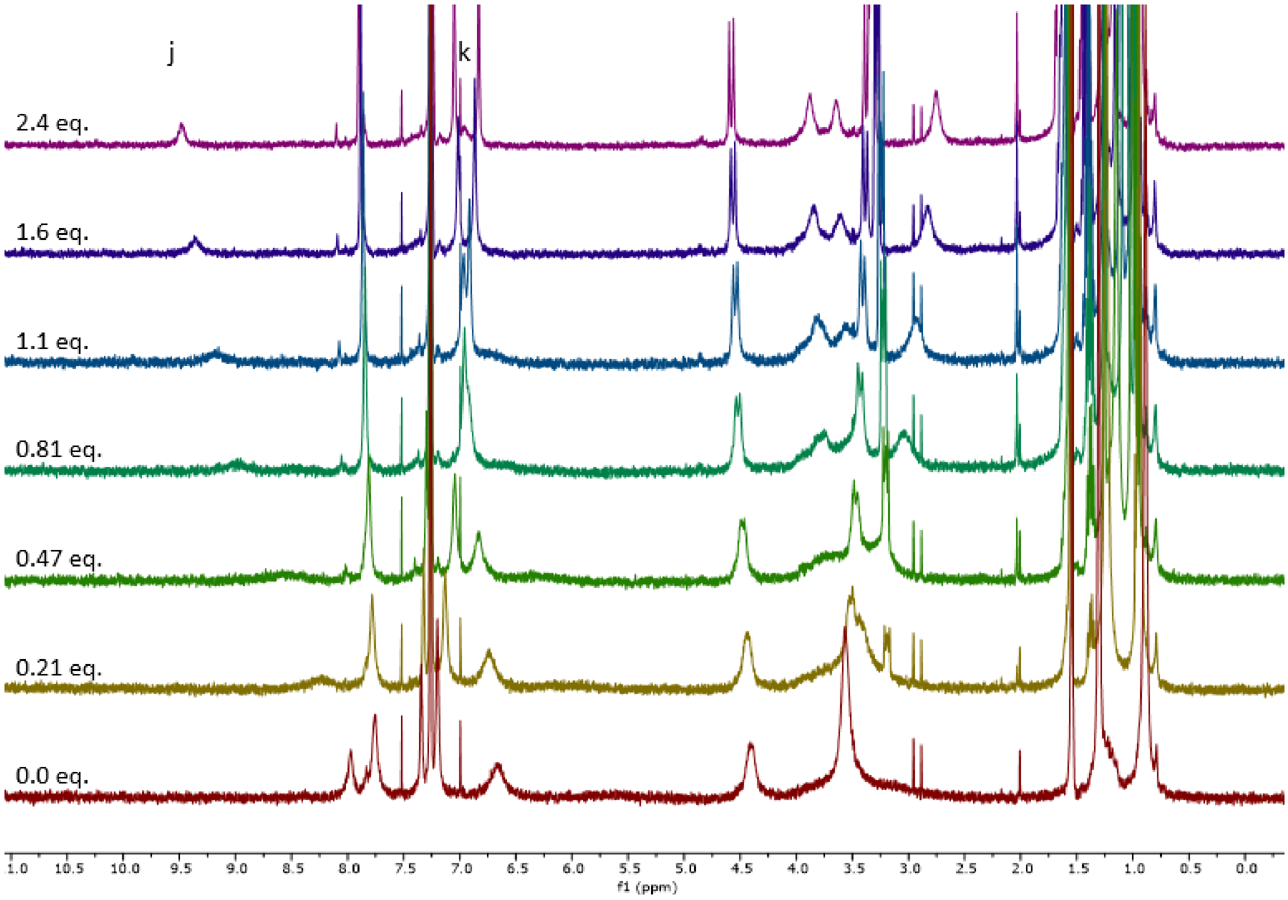

Figure S50 ^{1}H NMR spectra (400 MHz) from the titration of calixarene **4** (0.5 mM) with TBACl in $CDCl_3$ at 298 K. The number of equivalents of TBACl relative to **4** is shown.

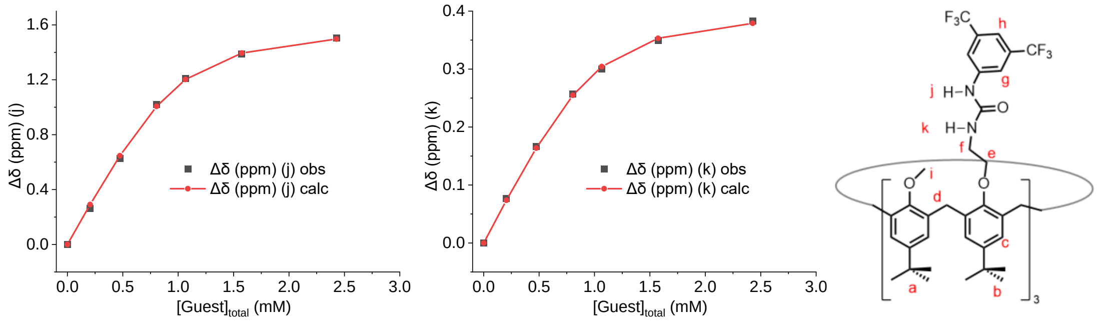

Figure S51 Observed changes in chemical shifts and calculated binding curves for the titration of **4** (0.5 mM) with TBACl at 298K in $CDCl_3$. The signals of protons 'j', 'k', were used for fitting to a 1:1 binding model, resulting in a K_a of 18242 M^{-1} (±8.6%).

^1H NMR titration of calixarene 5 with TBACl in $CDCl_3$

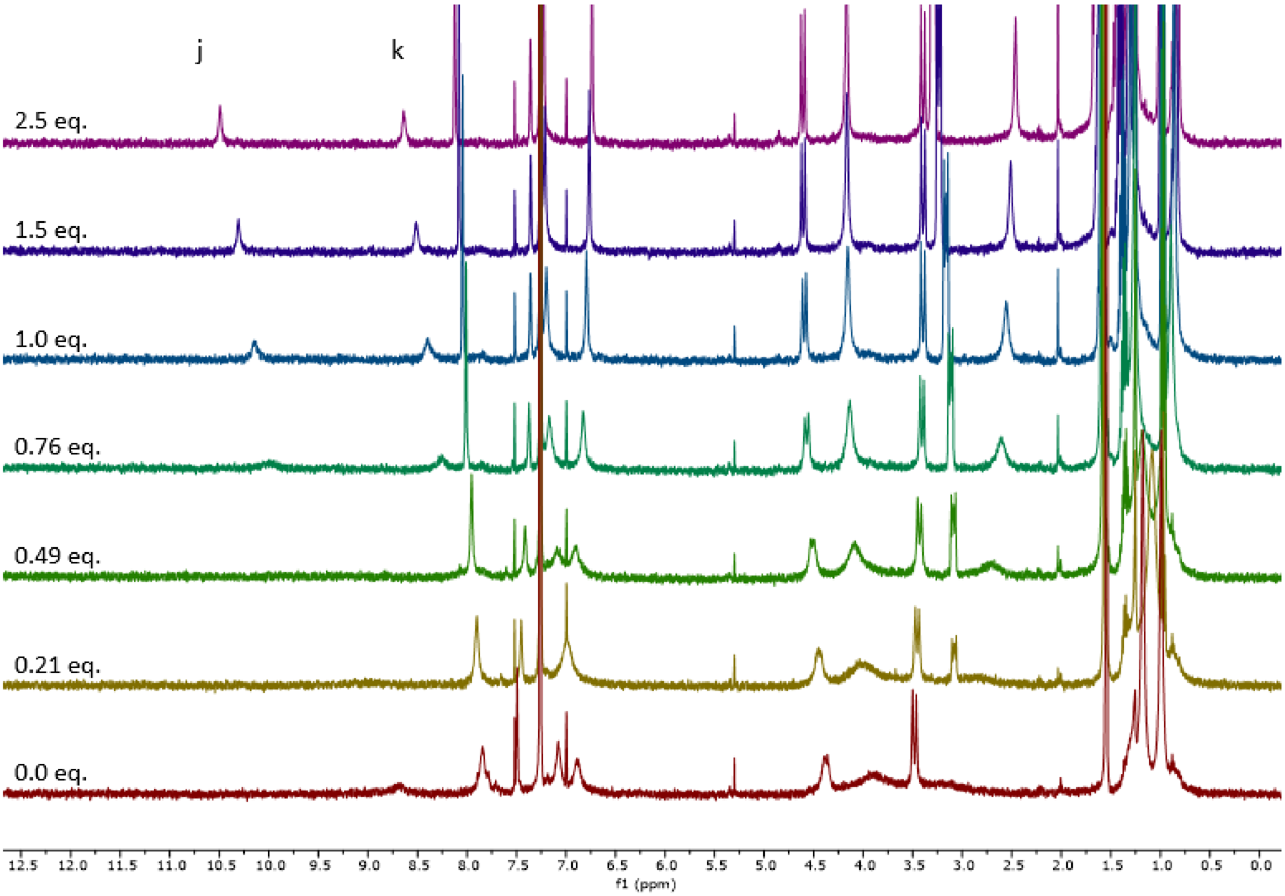

Figure S52 ^1H NMR spectra (400 MHz) from the titration of calixarene **5** (0.5 mM) with TBACl in $CDCl_3$ at 298 K. The number of equivalents of TBACl relative to **5** is shown.

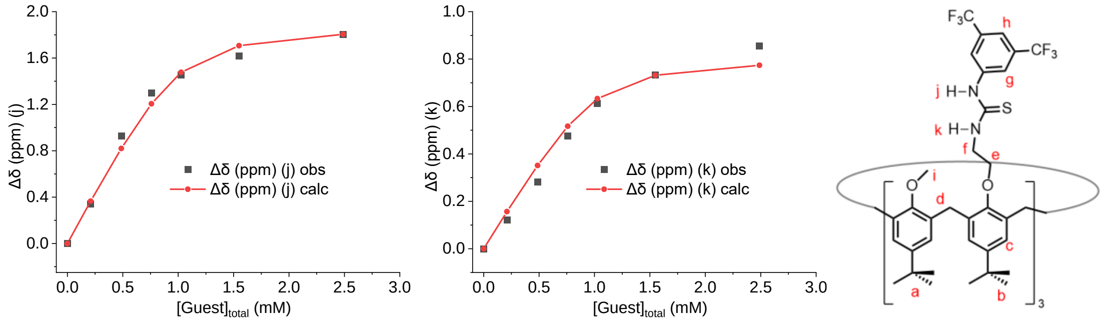

Figure S53 Observed changes in chemical shifts and calculated binding curves for the titration of **5** (0.5 mM) with TBACl at 298K in $CDCl_3$. The signals of protons 'j', 'k', were used for fitting to a 1:1 binding model, resulting in a K_a of 29365 M^{-1} (±44.6%).

3.3 Binding studies in DMSO

^{1}H NMR titration of calixarene 3 with TBACl in DMSO-d_6/0.5% H_2O

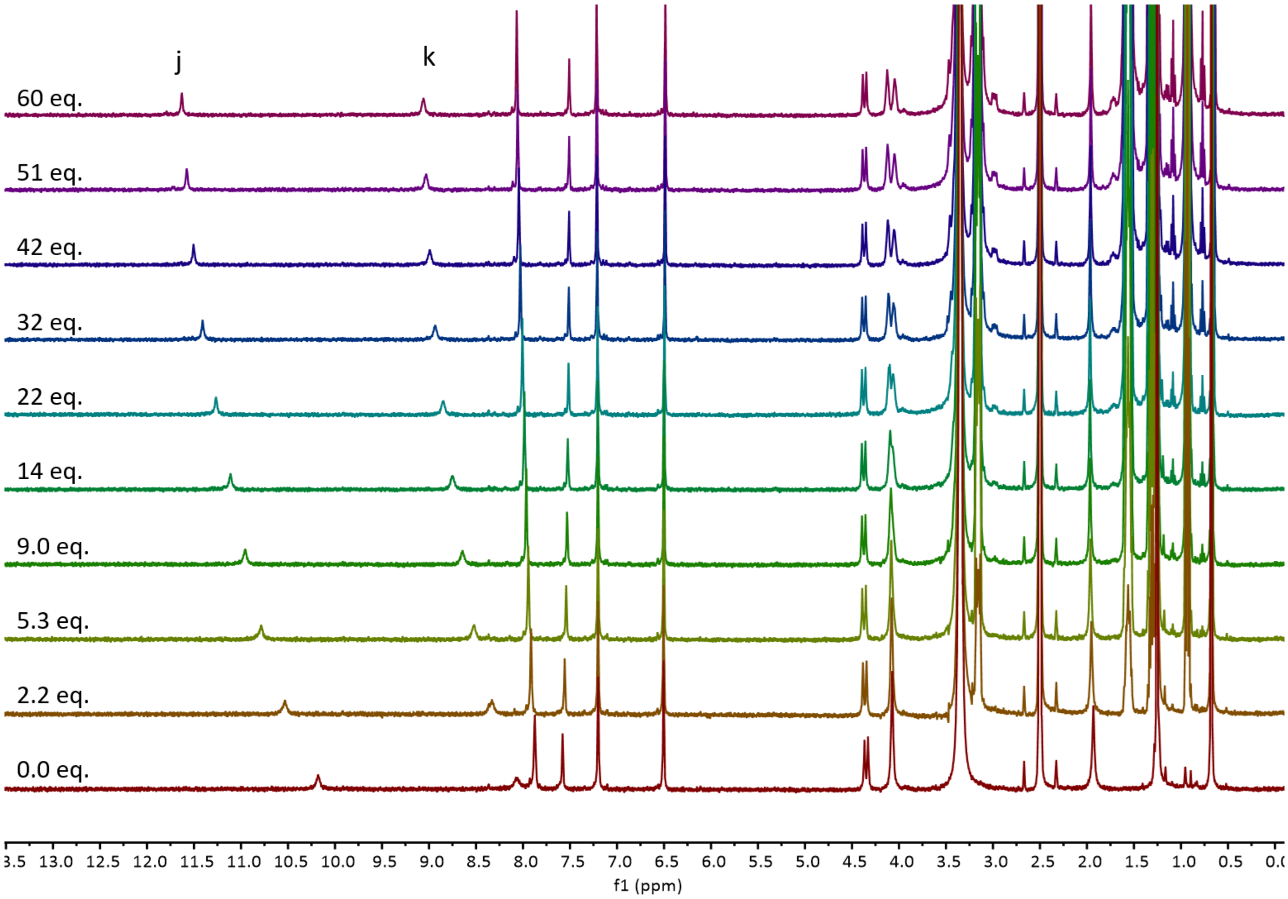

Figure S54 ^{1}H NMR spectra (400 MHz, 298 K, DMSO-d_6) from the titration of calixarene **3** (1 mM) with TBACl in DMSO-d_6/0.5% H_2O at 298 K. The number of equivalents of TBACl relative to **3** is shown.

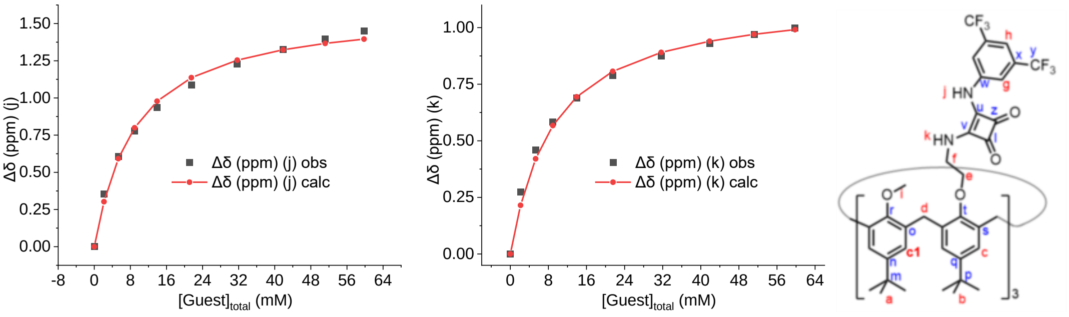

Figure S55 Observed changes in chemical shifts and calculated binding curves for the titration of **3** (1 mM) with TBACl at 298 K in DMSO-d_6/0.5% H_2O. The signals of protons 'j', 'k' were used for fitting to a 1:1 binding model, resulting in a K_a of 118 M^{-1} (±6.66 %).

In acetone-d_6, the affinity of **3** for Cl^- was found to be slightly lower compared to **4** and **5**. However, in DMSO-d_6 with 0.5% H_2O, receptor **3** had the highest affinity of series **3-5**.

Table S2 Chloride binding constants of calixarenes **3-5** in DMSO-d_6

**Compound**		**K_a for Cl^- in DMSO-d_6**
**3**	Squaramide	120
**4**	Urea	28[v]
**5**	Thiourea	38[v]

[v] *Previously reported values:* G. Grauwels, H. Valkenier, A. P. Davis, I. Jabin, K. Bartik, *Angew. Chem. Int. Ed.* **2019**, *58* (21), 6921–6925.

4. Transport studies in vesicles

4.1 Lucigenin assay

Preparation of the vesicles

Liposomes were prepared with sodium salts of various anions (NaA) both inside and outside, where the following aqueous solutions of NaA are used: $NaNO_3$ (225 mM), NaOAc (225 mM), and $NaHCO_3$ (225 mM, adjusted to pH 8 with a small amount of H_2SO_4).

Phospholipid and cholesterol solutions were combined with solutions of transporters in deacidified chloroform or methanol in a 5 mL round-bottomed flask. Volumes were calculated from the lipid concentrations to obtain a final concentration of 0.4 mM in lipids (POPC + cholesterol), with a 7:3 POPC to cholesterol ratio and a 1:1000, 1:5000, or 1:25’000 transporter to lipid ratio. The solvents from the mixture were evaporated under a flow of dry air and the resulting lipid film was dried in vacuum for at least 1 h. The lipid film was then hydrated with 500 μL of an aqueous solution of 10,10'-dimethyl-9,9'-biacridinium nitrate (lucigenin, 0.8 mM) and NaA (225 mM), sonicated for ca. 30 s and stirred for at least 1 h at room temperature. The heterogeneous multilamellar vesicles were broken down into unilamellar vesicles by 10 freeze-thawing cycles, diluted to 1 mL with NaA solution, and extruded 29 times through polycarbonate membranes (200 nm pore size) at room temperature to give homogeneous large unilamellar vesicles. The external lucigenin was removed by passing the vesicle solution through size exclusion columns (Sephadex G-25) eluted with its respective salt solution. The resulting vesicles were further diluted with the same salt solution to obtain a final concentration of 0.4 mM in lipids (calculated from the initial quantities of lipids).

General procedure for transport measurements with lucigenin

Fluorescence measurements were performed on 3 mL of the final vesicle solution in a quartz cell with a stir bar, and the fluorescence intensity of lucigenin (excitation at 430 nm and emission at 505 nm) was recorded over time. The temperature of the cuvette holder was controlled with a water bath at 25 °C. 75 μL of aqueous NaCl (1 M, in the same salt solution as the liposomes, to give an external chloride concentration of 25 mM) was added ca. 30 seconds after starting the experiment and the fluorescence was recorded for an additional 10 minutes before lysing of the vesicles with 50 μL of Triton X-100 (5 wt.%) in water.

Each experiment was repeated at least 3 times and the fluorescence data were averaged after removing the initial drop (due to the quenching of remaining external fluorophore) and normalizing each fluorescence value (F) to the initial value (F_0). Traces of 500 seconds of transport data are plotted.

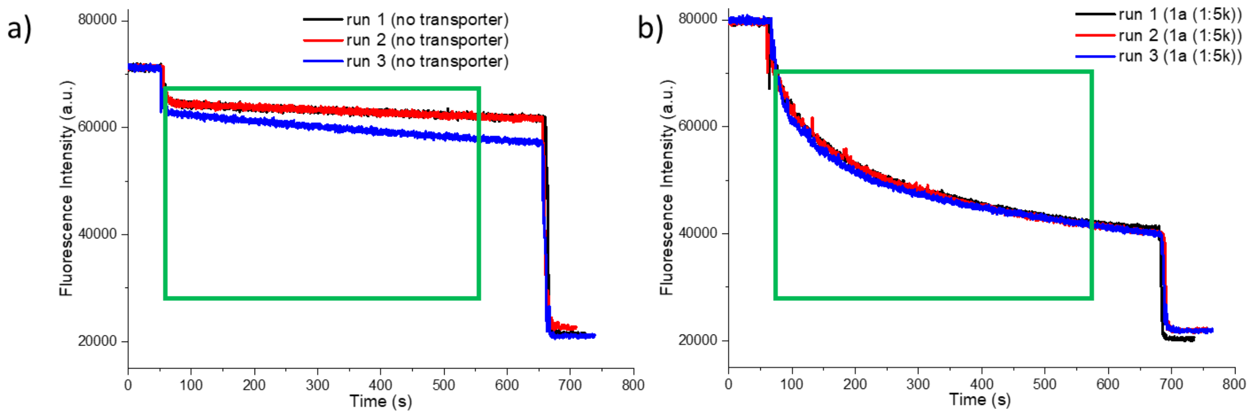

Figure S56 Representative transport curves for the lucigenin assay. a) No transport of Cl^- in the absence of transporter, b) Transport of Cl^- in the presence of **1a** (1:5k transporter to lipid ratio), as monitored by the lucigenin assay in 225 mM $NaNO_3$, upon addition of 25 mM NaCl. The green box indicates the part of the curves that is normalized, averaged, and plotted in Figure 3a and b.

All data recorded in NaCl solution are provided in the main text, while data for Cl^-/HCO_3^- and Cl^-/AcO^- antiport are provided in Figures S57-58.

Quantification of transport rates

The quantification of the transport rates (see Table 1) was performed as described previously.[vi] According to the Stern-Volmer equation, the inverse of the normalized fluorescence intensity (F_0/F) is directly proportional to the concentration of chloride inside the vesicles.

The obtained curve for F_0/F (0-500 s) is fitted to the double exponential function:

$$F_0/F = y - a \cdot e^{-bt} - c \cdot e^{-dt}$$

Differentiating the function with respect to t at t=0 gives

$$I = a \cdot b + c \cdot d$$

Further, the specific initial rate [I] is obtained by dividing the initial rate I by transporter to lipid ratio.

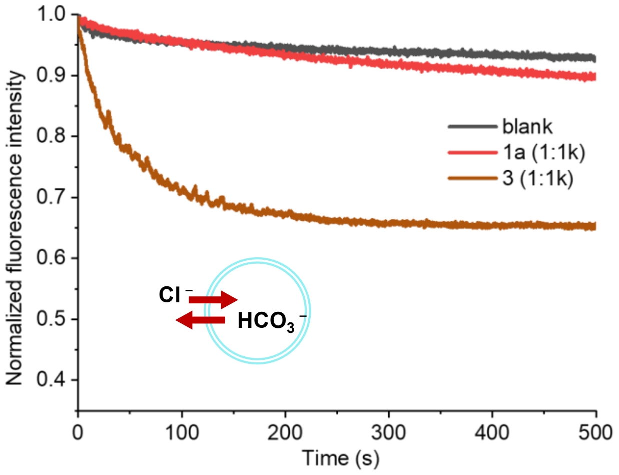

Figure S57 Transport of Cl⁻ by compounds **1a** and **3** (preincorporated in the vesicles at 1:1k transporter to lipid ratio) as monitored by the lucigenin assay in 225 mM $NaHCO_3$, upon addition of 25 mM NaCl.

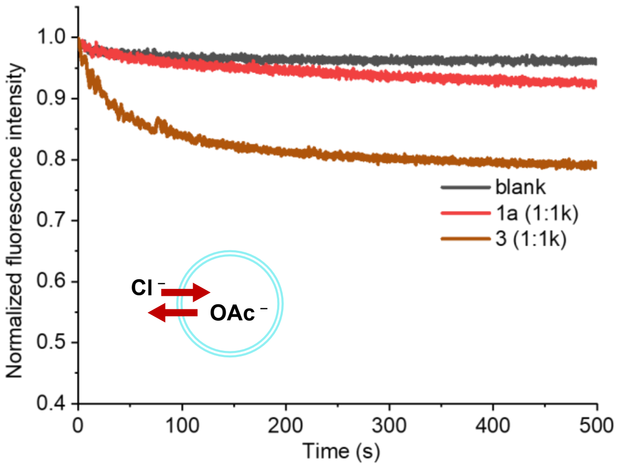

Figure S58 Transport of Cl⁻ by compounds **1a,** and **3** (preincorporated in the vesicles at 1:1k transporter to lipid ratio) as monitored by the lucigenin assay in 225 mM NaOAc, upon addition of 25 mM NaCl.

[vi] H. Valkenier, L. W. Judd, H. Li, S. Hussain, D. N. Sheppard and A. P. Davis, *J. Am. Chem. Soc.*, 2014, **136**, 12507–12512.

4.2 HPTS assay with a NMDG base pulse

A 1.0 M solution of N-methyl-D-glucamine (NMDG) was prepared and 50 mL of this solution was combined with the appropriate amounts of HCl and HEPES to prepare 500 mL aqueous solution of N methyl-D-glucamine hydrochloride ($NMDGH^+Cl^-$, 100 mM) and HEPES (10 mM) at pH 6.8. Similarly, a solution of $NMDGH^+NO_3^-$ (100 mM) and HEPEs (10 mM) at pH 6.8 was prepared by combining and diluting solutions of NMDG, HNO_3, and HEPES. HPTS was dissolved in these $NMDGH^+A^-$ solutions at a concentration of 0.1 mM.

POPC and cholesterol solutions (15-20 mM) in deacidified chloroform were combined with a solution of test receptor (1 mM in chloroform) in a 5 mL round bottom flask to obtain a POPC:cholesterol ratio of 7:3 and ratios of receptors to the total amount of lipids of 1:1000, 1:5000 and 1:25000. The solvents were evaporated under a flow of air and the resulting lipid film was dried under high vacuum for 1 h. The lipid film was then hydrated with 500 µL of a solution of 0.1 mM HPTS in the solution of $NMDGH^+A^-$ at pH 6.8. The resulting mixture was sonicated for 30 s and stirred for 1 h to give heterogeneous vesicles. Multilamellar vesicles were disrupted by 10 freeze-thaw cycles. The mixture was diluted to 1 mL (by adding 0.5 mL of $NMDGH^+A^-$ solution) and carefully extruded 29 times through a polycarbonate membrane with 200 nm pores in a mini-extruder (Avestin LiposoFast-Basic). The external HPTS was removed by passing the liposomes though a pre-packed size exclusion column (containing 8.3 mL Sephadex G-25 medium), eluted with $NMDGH^+A^-$ solution. The collected large unilamellar vesicles were further diluted with $NMDGH^+A^-$ solution to obtain a solution with 0.1 mM total lipid concentration at pH 6.8.

General procedure for transport measurements with HPTS

3.00 mL of this liposome solution was placed in a quartz cuvette with a small stir bar and the temperature was allowed to stabilize at 25 ˚C for 3-5 minutes inside the sample compartment of a Fluoromax-4 spectrometer. The fluorescence intensities (excitation at 403 and 455 nm, emission at 511 nm) were measured over time at 25 ˚C while stirring the solution, and 30 µL of NMDG (0.5 M in water) was added 30 seconds after the start of the measurement to increase the external pH to 7.7. The dissipation of this pH gradient was monitored by following the fluorescence for 200 seconds, after which the liposomes were lysed by addition of 60 µL of Triton X-100 (5% w/w in water). These experiments were repeated with carbonyl cyanide 3-chlorophenylhydrazone (CCCP, 5 µL of a 60 µM solution in methanol; 1:1k CCCP to lipid ratio) added to the liposomes (3 minutes before the start of the measurement), serving as proton transporter. The fluorescence intensity with excitation at 455 nm (for deprotonated HPTS) was divided by the fluorescence intensity with excitation at 403 nm (for protonated HPTS). The ratios of intensities over time were normalized from 0 (before addition of base) to 1 (plateau upon lysing of the liposomes) according to the formula below.

$$Normalized\ curves = \frac{\left(I_{455}/I_{403}\right)_t - \left(I_{455}/I_{403}\right)_{t0}}{\left(I_{455}/I_{403}\right)_{t\infty} - \left(I_{455}/I_{403}\right)_{t0}}$$

Determination of the EC_{50} value

As concentrations higher than 1:1000 transporters per lipid could not be pre-incorporated into the LUVs reliably, no plateau of transport could be reached, and the data could not be fitted to the Hill equation. Therefore, the EC_{50} value for the transport response by **1a** (in presence of CCCP, at 220s) was estimated from a logarithmic fit as 50% of the response from the normalized intensity ratio in absence of transporter (at 220 s, 0%) to the lysis level (100%).

HPTS assay in NMDGH$^+$Cl$^-$

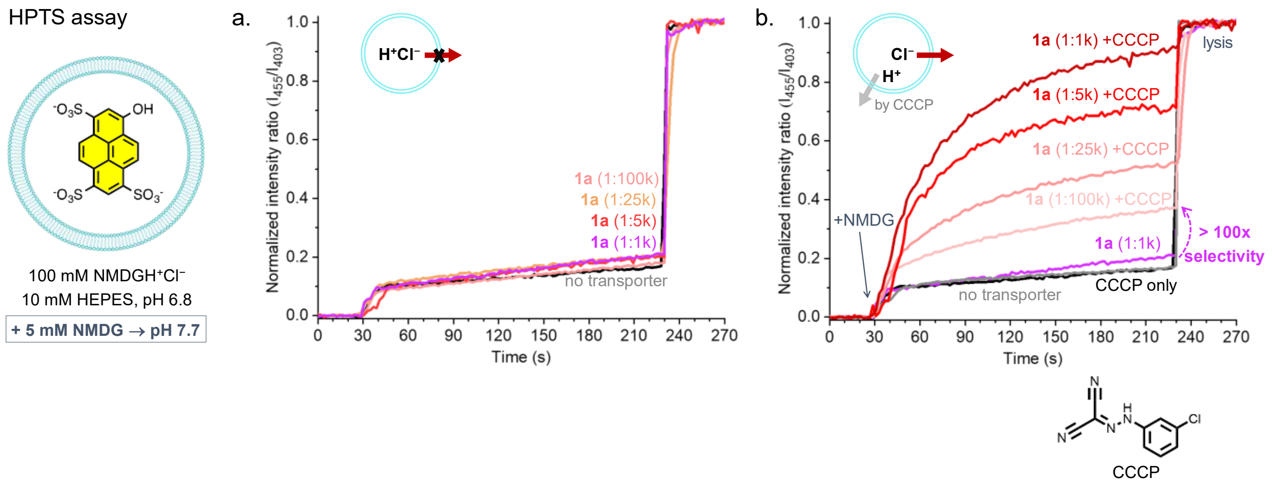

Figure S59 Dissipation of a pH gradient, generated by addition of NMDG at 30 s, by **1a** as monitored by changes in the fluorescence emission of HPTS (at 513 nm) upon excitation at 455 nm and at 403 nm; a. in absence of CCCP, and b. protonophore CCCP (1:1000) was added prior to the start of the experiments. The LUVs were lysed at 230 s.

HPTS assay in NMDGH$^+$NO$_3^-$

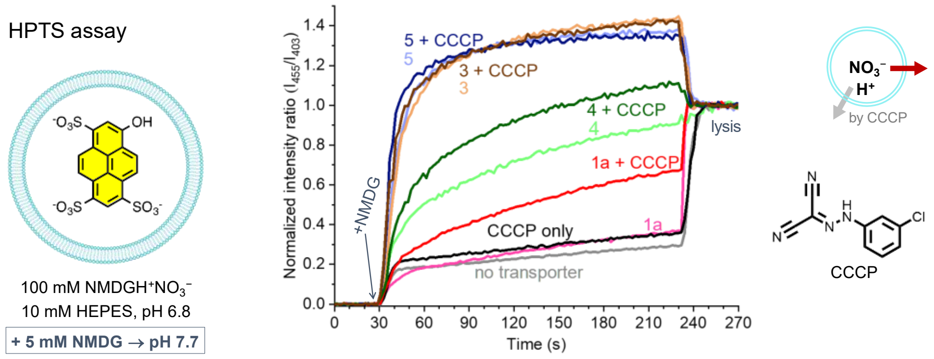

Figure S60 Dissipation of a pH gradient, generated by addition of NMDG at 30 s, by compounds **1a**, **3**, **4**, and **5** (1:5k) as, monitored by the ratio of the fluorescence emission of HPTS (at 513 nm) upon excitation at 455 nm and at 403 nm in absence of CCCP, and with CCCP (1:1k) added to the liposomes. The LUVs were lysed at 230 s.

These studies on the rate of Cl$^-$ and NO$_3^-$ uniport in HPTS-based assays show higher transport rates for **1a** (1:1k) in NMDGHCl than in NMDGHNO$_3$ (Figure S59 and S60), indicating that Cl$^-$ is transported faster than NO$_3^-$. The absence of Cl$^-$/HCO$_3^-$ and Cl$^-$/AcO$^-$ in lucigenin assays (Figure S57 and S58) and of Cl$^-$/OH$^-$ in the HPTS assay led to the conclusions that HCO$_3^-$, AcO$^-$, OH$^-$ are not transported by **1a**. Thus, the anion transport selectivity of **1a** follows the order: Cl$^-$ > NO$_3^-$ >>> HCO$_3^-$, AcO$^-$, OH$^-$.

4.3 HPTS assay with a TBAOH base pulse

A 100 mM solution of sodium gluconate was buffered at pH 7.0 with 10 mM HEPES. HPTS was dissolved in this 100 mM sodium gluconate solution at a concentration of 0.1 mM.

POPC and cholesterol solutions (15-20 mM) in deacidified chloroform were combined with a solution of receptor (1 mM in chloroform) in a 5 mL round bottom flask to obtain a POPC:cholesterol ratio of 7:3 and ratios of receptors to the total amount of lipids of 1:5k. The solvents were evaporated under a flow of air and the resulting lipid film was dried under high vacuum for 1 h. The lipid film was then hydrated with 500 µL of a solution of 0.1 mM HPTS in the solution of 100 mM sodium gluconate buffered at pH 7.0 with 10 mM HEPES. The resulting mixture was sonicated for 30 s and stirred for 1 h to give heterogeneous vesicles. Multilamellar vesicles were disrupted by 10 freeze-thaw cycles. The mixture was diluted to 1 mL (by adding 0.5 mL of buffered sodium gluconate solution) and carefully extruded 29 times through a polycarbonate membrane with 200 nm pores in a mini-extruder (Avestin LiposoFast-Basic). The external HPTS was removed by passing the liposomes though a pre-packed size exclusion column (containing 8.3 mL Sephadex G-25 medium), eluted with buffered sodium gluconate solution. The collected large unilamellar vesicles were further diluted with sodium gluconate solution to obtain a solution with 0.1 mM total lipid concentration at pH 7.0.

The transport experiments were performed in the same way as described for those with the HPTS assay and adding a NMDG base pulse. The only difference is that here 30 µL of 0.5 M tetrabutylammonium hydroxide (TBAOH, in the solution of 100 mM sodium gluconate) was added 30 seconds after the start of the measurement to increase the external pH to 7.6-7.7.

In this assay, the movement of charge associated with the transport of protons or hydroxide by the receptors can be balanced by the free diffusion of the tetrabutylammonium cation through the membrane, while gluconate anions are too polar to allow their transport.[vii] Also in this assay, **1a** does not show any transport activity, indicating that this compound cannot transport H^+ nor OH^-.

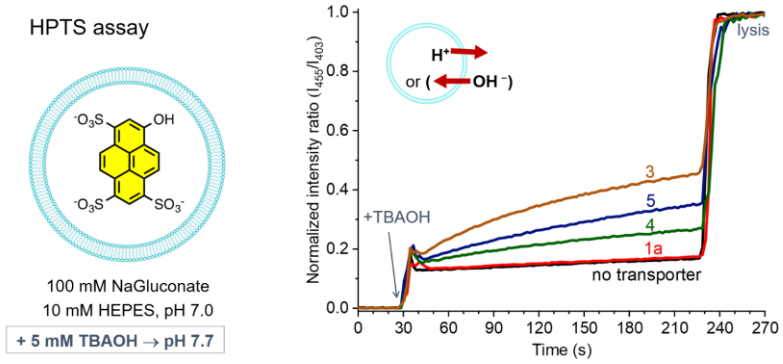

Figure S61 Dissipation of a pH gradient, generated by addition of TBAOH at 30 s, by compounds **1a**, **3**, **4**, and **5** (1:5k), as monitored by the ratio of the fluorescence emission of HPTS (at 511 nm) upon excitation at 455 nm and at 403 nm. The LUVs were lysed at 230 s.

[vii] X. Wu, L. W. Judd, E. N. W. Howe, A. M. Withecombe, V. Soto-Cerrato, H. Li, N. Busschaert, H. Valkenier, R. Pérez-Tomás, D. N. Sheppard, Y.-B. Jiang, A. P. Davis and P. A. Gale, *Chem*, 2016, **1**, 127–146.

5. Single-crystal X-ray diffraction analysis

Crystals suitable for single-crystal X-ray diffraction analysis were obtained by the slow evaporation of **1a** in dichloromethane at room temperature. Diffraction data were collected using the Oxford Diffraction Gemini R Ultra diffractometer (Cu Kα, multilayer mirror, Ruby CCD area detector) at 120(2) K. Intensity measurements were performed on a rapidly cooled crystal (0.35 x 0.30 x 0.22 mm^3) in the range 2.55° ≤ θ ≤ 67.14°. Data collection, unit cells determination and data reduction were carried out using CrysAlis PRO software package[viii] using Olex2[ix] and shelXle[xa], the structure was solved with the SHELXT 2015[xb] structure solution program by Intrinsic Phasing methods and refined by full-matrix least squares on $|F|^2$ using SHELXL-2018/3[xb]

Non-hydrogen atoms were refined anisotropically, while C–H hydrogen atoms were placed in geometrically calculated positions using a riding model. In the asymmetric unit, the 2-{4-[3,5-bis(trifluoromethyl)phenyl]-5-iodo-1H-1,2,3-triazol-1-yl}ethan-1-oxy- group is disordered over three positions in such way that in two position the iodine atom orientated towards the cavity and one orientated in the opposite direction). When the iodine orientated towards the cavity it is coordinated to water molecule. Occupancies of two first positions are equal to occupancy of the water molecule. Disordered hydrogen atoms of water were omitted from the refinement. Besides, the asymmetric unit also contains one molecule of dichloromethane disordered over 3-fold axis. CCDC 2125602 contains the supplementary crystallographic data for this paper. Copies of the data can be obtained free of charge via http:/www.ccdc.cam.ac.uk/const/retrieving.html or from the Cambridge Crystallographic Data Centre, 12 Union Road, Cambridge CB2 1EZ, UK; fax: (+44)1223-336-033; e-mail: deposit@ccdc.cam.ac.uk). The most relevant crystallographic data are summarized in Table S3.

Table S3: Selected crystallographic data for compounds **1a**.

Crystallographic data	**1a**		
chemical formula	$C_{105}H_{108}F_{18}I_3N_9O_6 \cdot 0.723(CH_2Cl_2) \cdot 0.443(H_2O)$		
fw (g·mol^{-1})	2382.62		
crystal system	Trigonal		
space group	$R\bar{3}c$		
a/Å	23.1570(3)		
b/Å	23.1570(3)		
c/Å	68.8908(12)		
α/°	90		
β/°	90		
γ/°	120		
V/Å^3	31993.1(10)		
Z	12		
T/K	120(2)		
λ/Å	1.54184		
μ/mm^{-1}	7.995		
ρ_{calcd} /g·cm^{-3}	1.484		
F(000)	14442.0		
crystal size/mm^3	0.35x0.30x0.22		
θ Range/°	2.549 to 67.14		
Limiting indices	-19 ≤ h ≤ 26	-27 ≤ k ≤ 27	-69 ≤ l ≤ 81
Reflections collected	6343		
Reflections unique (R_{int})	5880 (0.036)		
No. of data / restraints / parameters	6343/780/840		
Goodness of fit on $\|F\|^2$	1.110		
R_1,[a] wR_2[b] ($I > 2\sigma(I)$)	0.0724, 0.1766		
R_1,[a] wR_2[b] (all data)	0.0758, 0.1786		
Residual electron density/e·Å^{-3}	0.748/-0.845		
CCDC deposition number	2125602		

[viii] Rigaku Oxford Diffraction, (2020), CrysAlisPro Software system, version 1.171.40.82a, Rigaku Corporation, Wroclaw, Poland.
[ix] O. V. Dolomanov, L. J. Bourhis, R. J. Gildea, J. A. K. Howard, H. Puschmann, *J. Appl. Crystallogr.* **2009**, *42* (2), 339–341.
[x] a) C. B. Hübschle, G. M.; Sheldrick, B. Dittrich, *J. Appl. Crystallogr.* **2011**, *44* (6), 1281–1284. b) G. M. Sheldrick, *Acta Crystallogr. Sect. A Found. Crystallogr.* **2015**, *71* (1), 3–8.

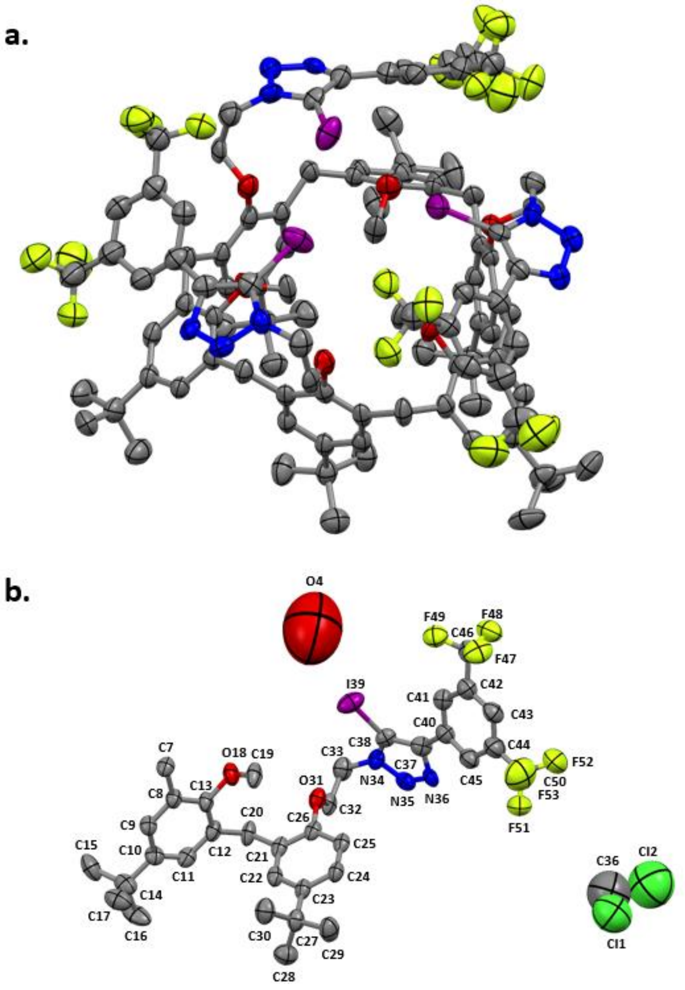

Figure S62 a) Molecular structure and b) asymmetric unit of **1a** with thermal ellipsoids at 50 % probability level. Hydrogen atoms and the minor components of the disordered 2-{4-[3,5-bis(trifluoromethyl)phenyl]-5-iodo-1H-1,2,3-triazol-1-yl}ethan-1-oxy- component are omitted for the sake of clarity.

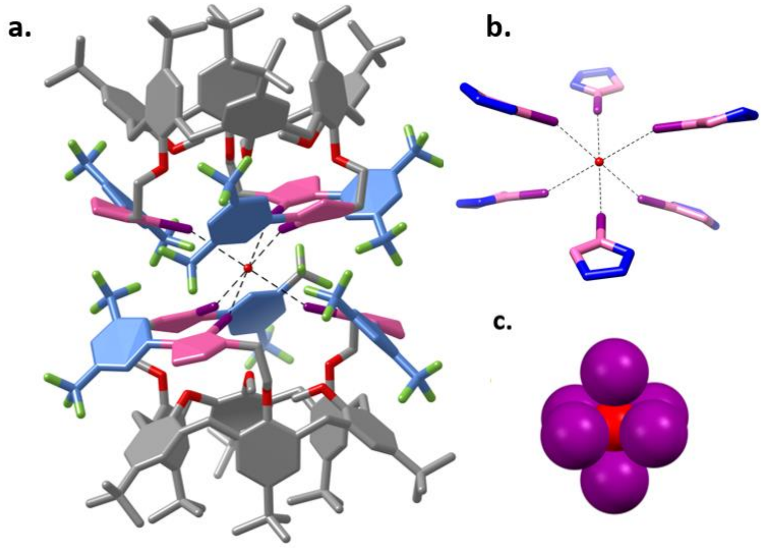

Figure S63 a) Fragment of the crystal lattice showing the interaction of one water molecule with two molecules of **1a**. b) O···I interaction between six iodotriazole groups with the solvent. c) Spacefill model showing the geometry of coordination around the oxygen atom from the water molecule. Hydrogen atoms and minor parts of the disorder are omitted for the sake of clarity.
